# Antiparasitic Behavior of Trifluoromethylated Pyrazole 2-Amino-1,3,4-thiadiazole Hybrids and Their Analogues: Synthesis and Structure-Activity Relationship

**DOI:** 10.3389/fphar.2020.591570

**Published:** 2020-10-07

**Authors:** Jeniffer do Nascimento Ascencio Camargo, Karlos Eduardo Pianoski, Mariellen Guilherme dos Santos, Danielle Lazarin-Bidóia, Hélito Volpato, Sidnei Moura, Celso Vataru Nakamura, Fernanda Andreia Rosa

**Affiliations:** ^1^ Departamento de Química, Universidade Estadual de Maringá (UEM), Maringá, Brazil; ^2^ Laboratório de Inovação Tecnológica no Desenvolvimento de Fármacos e Cosméticos, Departamento de Ciências Básicas da Saúde, Universidade Estadual de Maringá (UEM), Maringá, Brazil; ^3^ Instituto de Biotecnologia, Universidade de Caxias do Sul (UCS), Caxias do Sul, Brazil

**Keywords:** trifluoromethylated pyrazoles, thiadiazole, thiosemicarbazone, antileishmanial, antitrypanosomal****

## Abstract

A series of trifluoromethylated pyrazole thiosemicarbazone, trifluromethylated pyrazole isothiosemicarbazone, and trifluoromethylated pyrazole 2-amino-1,3,4-thiadiazole hybrids were synthesized and evaluated *in vitro* against the promastigote form of *Leishmania amazonensis* and the epimastigote form of *Trypanosoma cruzi*, the pathogens causing the neglected tropical diseases leishmaniasis and Chagas disease, respectively. The results show the potential of these compounds regarding their antiparasitic properties. Studies on the structure-activity relationship demonstrated that compounds containing a bulky group at the *para* position of the phenyl ring attached to the 5-position of the pyrazole core had better antiparasitic effects. Among the substituents attached at the 3-position of the pyrazole ring, the insertion of the 2-amino-1,3,4-thiadiazole nucleus led to the most potent compounds compared to the thiosemicarbazone derivative.

## Introduction

Every year, more than 700,000 deaths occur due to infectious diseases caused by parasites (Habercom, 2016). Among them, neglected tropical diseases (NTD) are responsible for about 530,000 deaths (WHO). Leishmaniasis is a NTD caused by *Leishmania* species and transmitted by the phlebotomine sandfly vector that can be found throughout the world, but mostly in developing countries, putting at risk more than 350 million lives (Habercom, 2016; Gradoni et al., 2017).

The drugs that are used nowadays for the treatment of leishmaniasis include pentavalent antimonials as first-line drugs, such as meglumine antimoniate. In case of resistance, other drugs are used like amphotericin B, paromomycin, miltefosine, and pentamidine (Alvar et al., 2012; Sangshetti et al., 2015; Hurrell et al., 2016). However, all these drugs present high toxicity and several collateral effects, beyond resistance of the protozoan to the drugs (Sangshetti et al., 2015).

Furthermore, Chagas disease (CD), also known as American trypanosomiasis, is another NTD that also occurs in developing countries, caused by the vector-borne flagellate protozoan parasite *Trypanosoma* *cruzi.* Chagas disease has infected over 20 million people in Central and South America and is responsible for around 20,000 deaths per year. Current treatments for Chagas disease are based on two old drugs, benznidazole (BZN) and nifurtimox (NFX) (Goupil and McKerrow, 2014; Chain et al., 2019). They are more effective in the acute phase of the disease, show several side effects, and require prolonged treatment. Therefore, there is remarkable urgency for the development of effective, inexpensive, and safe drugs for the treatment of NTD such as leishmaniasis and Chagas disease.

**Figure 1:**
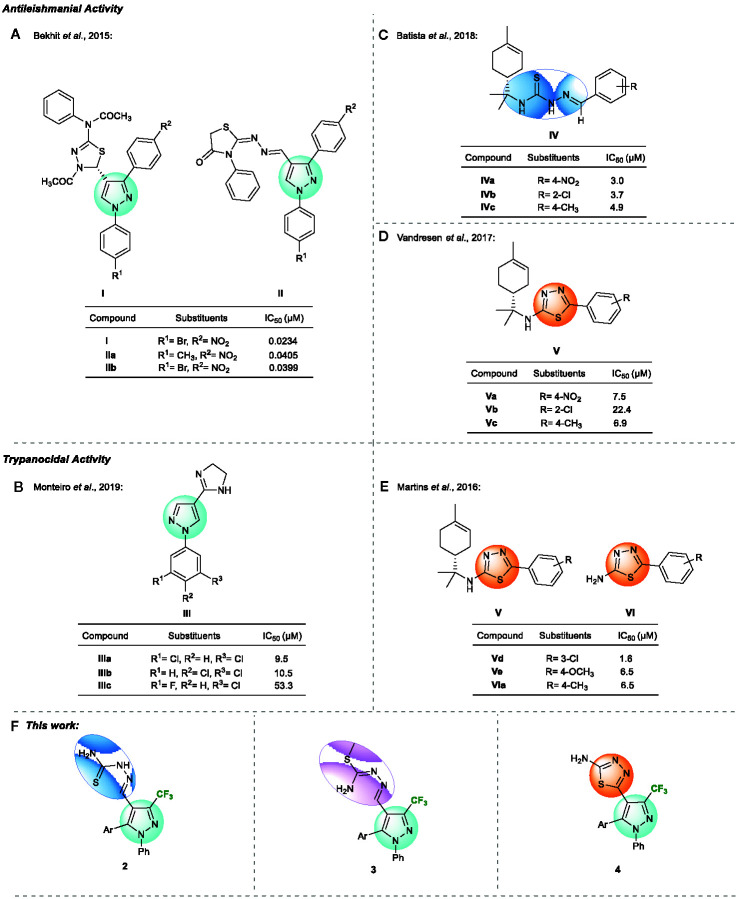
**(A–E)** Previews works that related structures with antileishmanial and trypanocidal activity. **(F)** Structures proposed in this work.

Several studies have shown the potential pharmacological effects of nitrogen-containing five-membered heterocycles. Pyrazole-based compounds have been reported as important small molecules in drug discovery for NTDs (Bekhit et al., 2015; Monteiro et al., 2019). For instance, Bekhit and coworkers in 2015 reported the antileishmanial activity of heterocycle hybrids containing the pyrazole moiety on *Leishmania aethiopica* (**Figure 1A**), whereas Monteiro and coworkers in 2019 reported the trypanocidal activity of pyrazole derivatives on *T. cruzi* (**Figure 1B**).

Moreover, 1,3,4-thiadiazole derivatives have exhibited a broad spectrum of pharmacological properties. They have a mesoionic characteristic that allow strong interactions with biomolecules. Also, the sulfur atom in this heterocycle imparts improved liposolubility. More specifically, 2-amino-1,3,4-thiadiazole series (Jain et al., 2013; Martins et al., 2016; Vandresen et al., 2017; Serban, 2019; Freitas et al., 2020) and their thiosemicarbazone analogue (De Melos et al., 2015; Batista et al., 2019) have been studied for potential antileishmanial and trypanocidal activity (**Figures 1C–E**). Comparing the results reported for compounds **IV** and **V**, the 2-amino-1,3,4-thiadiazole derivatives were less active than the thiosemicarbazone derivatives (**Figures 1C, D**).

Additionally, the presence of fluorine atoms in organic compounds affect the chemical, biological, and physical properties of the molecules. Therefore, a trifluoromethyl group (-CF_3_) can improve the pharmacological activities of compounds bearing this moiety (Petrov, 2009).

With the purpose of developing potent antiprotozoal compounds, that are less toxic and more selective, in this study, we adopted the molecular hybridization strategy for molecules design and synthesized a new series of trifluoromethylated pyrazoles and thiosemicarbazone/*S*-methylated thiosemicarbazone/2-amino 1,3,4-thiadiazoles hybrids (**Figure 1F**), and evaluated their antiprotozoal activity against the promastigote form of *Leishmania amazonensis* and the epimastigote form of *Trypanosoma* *cruzi*.

## Experimental Section

### General Synthetic Procedure and Spectra Data

#### Synthesis of 5-aryl-4-[(2-Carbamothioyl-Hydrazinylidene)Methyl]-3-Trifluoromethyl-1-Phenyl-*1H*-Pyrazole (2a–f)

##### General Method

The cyclocondensation reaction was performed according to the methodology described by Pianoski et al. (2020). The trifluoromethylated *β*-enamino diketone **1** (**1a**: 0.229 g; **1b**: 0.317 g; **1c**: 0.334 g; **1d**: 0.378 g; **1e**: 0.334 g; **1f**: 0.329, 1.0 mmol, 1.0 equiv.) was solubilized in MeCN (10.0 ml), then added phenylhydrazine (0.108 g, 1.0 mmol, 1.0 equiv) and boron trifluoride diethyl etherate solution 46.5% (0.400 ml, 1.5 mmol, 1.5 equiv.). The mixture was stirred under reflux for 7 h. In sequence, the reaction mixture was cooled to room temperature, added thiosemicarbazide (0.276 g, 3.0 mmol, 3.0 equiv.) and stirred for 30 min. Then, the solvent was evaporated under vacuum and the residue was washed with a solution of 3% of K_2_CO_3_ (25 ml), extracted with dichloromethane (3 × 20 ml), and dried with anhydrous sodium sulfate. The solvent was evaporated under reduced pressure and the obtained residue was dissolved in hot ethyl ether (5 ml) and cooled to 0°C which induced crystallization. The solid was filtered, washed with cold ethyl ether (20 ml), and dried under vacuum.

**4-[(2-carbamothioyl-hydrazinylidene)methyl]-3-trifluoromethyl-5-(4-nitrophenyl)-1-phenyl-*1H-*pyrazole** (**2a**): Yellow solid; 94% yield (0.408 g); mp 226.5 °C; **^1^H NMR** (300.06 MHz, DMSO-*d_6_*) δ (ppm) 6.49 (*s*, 1H, CSNH_2_), 7.34–7.42 (*m*, 5H, C_6_H_5_), 7.69 (*d*, 2H, 4-NO_2_-C_6_H_4_, *J* = 8.9 Hz), 8.01 (*s*, 1H, CH), 8.25 (*d*, 2H, 4-NO_2_-C_6_H_4_, *J* = 8.9 Hz), 8.31 (*s*, 1H, CSNH_2_), 11.50 (*s*, 1H, NH); **^13^C NMR** (75.45 MHz, DMSO-*d_6_*) δ (ppm) 115.2 (C^4^), 121.5 (*q*, CF_3_, ^1^ *J*_C-F_ *=* 269.5* Hz*), 123.8 (4-NO_2_-C_6_H_4_), 126.0, 129.5, 129.5 (C_6_H_5_), 132.3 (4-NO_2_-C_6_H_4_), 132.6 (CH), 134.3 (4-NO_2_-C_6_H_4_), 137.8 (C_6_H_5_), 138.7 (*q*, C^3^, ^2^ *J*_C-F_ *=* 37.4* Hz*), 142.7 (C^5^), 148.1 (4-NO_2_-C_6_H_4_), 178.2 (C=S); **HRMS** (ESI+): calcd for C_18_H_14_F_3_N_6_O_2_S^+^, [M+H]^+^: 435.0846, found 435.0864.

**4-[(2-carbamothioyl-hydrazinylidene)methyl]-3-trifluoromethyl-5-(4-fluorophenyl)-1-phenyl-*1H-*pyrazole** (**2b**): White solid; 63% yield (0.256 g); mp 221.18°C; **^1^H NMR** (500.13 MHz, CDCl_3_) δ (ppm) 6.23 (*s*, 1H, CSNH_2_), 6.87 (*ls*, 1H, CSNH_2_), 7.09–7.15 (*m*, 2H, 4-F-C_6_H_4_), 7.20-7.24 (*m*, 4H, 4-F-C_6_H_4_ and C_6_H_5_), 7.33–7.38 (*m*, 3H, C_6_H_5_), 7.70 (*s*, 1H, CH), 9.61 (*s*, 1H, NH); **^13^C NMR** (125.76 MHz, CDCl_3_) δ (ppm) 114.1 (C^4^), 116.5 (*d*, 4-F-C_6_H_4_, ^2^ *J*_C-F_ *=* 22.0* Hz*), 121.2 (*q*, CF_3_, ^1^ *J*_C-F_ *=* 269.9* Hz*), 123.7 (*d*, 4-F-C_6_H_4_, ^4^ *J*_C-F_ *=* 3.6* Hz*), 125.4, 129.0, 129.4 (C_6_H_5_), 132.3 (*d*, 4-F-C_6_H_4_, ^3^ *J*_C-F_ *=* 8.5* Hz*), 133.7 (CH), 138.2 (C_6_H_5_), 140.3 (*q*, C^3^, ^2^ *J*_C-F_ *=* 38.2* Hz*), 144.1 (C^5^), 163.5 (*d*, 4-F-C_6_H_4_, ^1^ *J*_C-F_ *=* 252.1* Hz*), 178.2 (C=S); **HRMS** (ESI+): calcd for C_18_H_14_F_4_N_5_S^+^, [M+H]^+^: 408.0901, found 408.0918.

**4-[(2-carbamothioyl-hydrazinylidene)methyl]-5-(4-chlorophenyl)-3-trifluoromethyl-1-phenyl*-1H-*pyrazole** (**2c**): White solid; 78% yield (0.330 g); mp 225.37°C; **^1^H NMR** (500.13 MHz, DMSO-*d_6_*) δ (ppm) 6.64 (*s*, 1H, CSNH_2_), 7.32–7.44 (*m*, 7H, 4-Cl-C_6_H_4_ and C_6_H_5_), 7.52 (*d*, 2H, 4-Cl-C_6_H_4_, *J* = 8.6 Hz), 7.97 (*s*, 1H, CH), 8.43 (*s*, 1H, CSNH_2_), 11.51 (*s*, 1H, NH); **^13^C NMR** (125.76 MHz, DMSO-*d_6_*) δ (ppm) 114.6 (C^4^), 121.4 (*q*, CF_3_, ^1^ *J*_C-F_ *=* 269.2* Hz*), 125.9 (C_6_H_5_), 126.3, 128.8 (4-Cl-C_6_H_4_), 129.2, 129.3 (C_6_H_5_), 132.4, 134.8 (4-Cl-C_6_H_4_), 133.0 (CH), 137.9 (C_6_H_5_), 138.2 (*q*, C^3^, ^2^ *J*_C-F_ *=* 37.3* Hz*), 143.9 (C^5^), 178.0 (C=S); **HRMS** (ESI+): calcd for C_18_H_14_ClF_3_N_5_S^+^, [M+H]^+^: 424.0605, found 424.0625.

**5-(4-bromophenyl)-4-[(2-carbamothioyl-hydrazinylidene)methyl]-3-trifluoromethyl-1-phenyl*-1H-*pyrazole** (**2d**): White solid; 73% yield (0.342 g); mp 226.12°C; **^1^H NMR** (500.13 MHz, DMSO-*d_6_*) δ (ppm) 6.65 (*s*, 1H, CSNH_2_), 7.32-7–36 (*m*, 4H, C_6_H_5_ and 4-Br-C_6_H_4_), 7.42–7.45 (*m*, 3H, C_6_H_5_), 7.65 (*d*, 2H, 4-Br-C_6_H_4_, *J* = 8.5 Hz), 7.97 (*s*, 1H, CH), 8.43 (*s*, 1H, CSNH_2_), 11.50 (*s*, 1H, NH); **^13^C NMR** (125.76 MHz, DMSO-*d_6_*) δ (ppm) 114.6 (C^4^), 121.4 (*q*, CF_3_, ^1^ *J*_C-F_ *=* 269.3* Hz*), 125.8, 126.6 (4-Br-C_6_H_4_), 129.2, 129.3 (C_6_H_5_), 129.3, 131.7 (4-Br-C_6_H_4_), 132.6 (C_6_H_5_), 131.9 (4-Br-C_6_H_4_), 133.0 (CH), 137.9 (C_6_H_5_), 138.2 (*q*, C^3^, ^2^ *J*_C-F_ *=* 37.3* Hz*), 143.9 (C^5^), 178.0 (C=S); **HRMS** (ESI+): calcd for C_18_H_14_BrF_3_N_5_S^+^, [M+H]^+^: 468.0100, found 468.0102.

**4-[(2-carbamothioyl-hydrazinylidene)methyl]-3-trifluoromethyl-1,5-diphenyl-*1H-*pyrazole** (**2e**): White solid; 70% yield (0.272 g); mp 211.27°C; **^1^H NMR** (500.13 MHz, DMSO-*d_6_*) δ (ppm) 6.44 (*s*, 1H, CSNH_2_), 6.84 (*s*, 1H, CSNH_2_), 7.19–7.41 (*m*, 10H, C_6_H_5_ A and B), 7.79 (*s*, 1H, CH), 10.24 (*s*, 1H, NH); **^13^C NMR** (125.76 MHz, DMSO-*d_6_*) δ (ppm) 114.0 (C^4^), 121.3 (*q*, CF_3_, ^1^ *J*_C-F_ *=* 270.0* Hz*), 127.8, 128.8, 129.1, 129.2, 130.1, 130.2, 138.4 (C_6_H_5_ – A and B), 133.9 (CH), 140.3 (*q*, C^3^, ^2^ *J*_C-F_ *=* 38.4* Hz*), 145.1 (C^5^), 178.0 (C=S); **HRMS** (ESI+): calcd for C_18_H_15_F_3_N_5_S^+^, [M+H]^+^: 390.0995, found 390.1002.

**4-[(2-carbamothioyl-hydrazinylidene)methyl]-3-trifluoromethyl-5-(4-methoxyphenyl)-1-phenyl-*1H-*pyrazole** (**2f**): White solid; 63% yield (0.272 g); mp 212.65°C; **^1^H NMR** (500.13 MHz, CDCl_3_) δ (ppm) 3.83 (*s*, 3H, 4-OCH_3_-C_6_H_4_), 6.39 (*s*, 1H, CSNH_2_), 6.91 (*d*, 2H, 4-OCH_3_-C_6_H_4_ *, J* = 8.8 Hz), 6.95 (*s*, 1H, CSNH_2_), 7.12 (*d*, 2H, 4-OCH_3_-C_6_H_4_ *, J* = 8.8 Hz), 7.23–7.34 (*m*, 5H, C_6_H_5_), 7.76 (*s*, 1H, CH), 9.97 (*s*, 1H, NH); **^13^C NMR** (125.76 MHz, CDCl_3_) δ (ppm) 55.5 (4-OCH_3_-C_6_H_4_), 113.7 (C^4^), 114.6, 119.6 (4-OCH_3_-C_6_H_4_), 121.4 (*q*, CF_3_, ^1^ *J*_C-F_ *=* 269.7* Hz*), 125.3, 128.7, 129.3 (C_6_H_5_), 131.7 (4-OCH_3_-C_6_H_4_), 134.0 (CH), 138.5 (C_6_H_5_), 140.2 (*q*, C^3^, ^2^ *J*_C-F_ *=* 38.2* Hz*), 145.3 (C^5^), 160.8 (4-OCH_3_-C_6_H_4_), 178.0 (C=S); **HRMS** (ESI+): calcd for C_19_H_17_F_3_N_5_OS^+^, [M+H]^+^: 420.1100, found 420.1121.

#### Synthesis of 5-Aryl-4-[(2-(*S*-Methyl-Carbonimidothioyl-Hydrazinylidene)Methyl]-3-Trifluoromethyl-1-Phenyl-*1H*-Pyrazole (3a–f)

##### General Method

In a solution of thiosemicarbazone derivatives **2** (**2a**: 0.434 g; **2b**: 0.407 g; **2c**: 0.423 g; **2d**: 0.468 g; **2e**: 0.389 g; **2f**: 0.419, 1.0 mmol, 1.0 equiv.) in DMSO (5.0 ml) was added sodium carbonate (0.127 g, 1.2 mmol, 1.2 equiv.) and iodomethane solution 99% (0.063 ml, 1.0 mmol, 1.0 equiv.). The mixture was stirred under room temperature for 5 min. Then, the product was washed with distilled water (5 × 15.0 ml) and filtered under vacuum. The obtained residue was dissolved in a mixture of hexane/ethyl acetate (4:1) and cooled to 0°C, which induced crystallization. The solid was filtered, washed with cold hexane (20 ml) and dried under vacuum.

**4-[(2-(*S*-methyl-carbonimidothioyl-hydrazinylidene)methyl]-3-trifluoromethyl-5-(4-nitrophenyl)-1-phenyl-*1H-*pyrazole** (**3a**): Yellow solid; 85% yield (0.367 g); mp 190.7°C; **^1^H NMR** (300.06 MHz, CDCl_3_) δ (ppm) 2.45 (*s*, 3H, SCH_3_), 5.22 (*s*, 2H, NH_2_), 7.21–7.24 (*m*, 2H, C_6_H_5_), 7.34-7.37 (*m*, 3H, C_6_H_5_), 7.48 (*d*, 2H, 4-NO_2_-C_6_H_4_, *J* = 8.9 Hz), 8.21-8.24 (*m*, 3H, 4-NO_2_-C_6_H_4_ and CH); **^13^C NMR** (75.45 MHz, CDCl_3_) δ (ppm) 12.8 (SCH_3_), 116.8 (C^4^), 121.3 (*q*, CF_3_, ^1^ *J*_C-F_ *=* 269.8* Hz*), 123.9 (4-NO_2_-C_6_H_4_), 125.5, 129.2, 129.5 (C_6_H_5_), 131.6, 135.2 (4-NO_2_-C_6_H_4_), 138.1 (C_6_H_5_), 141.2 (*q*, C^3^, ^2^ *J*_C-F_ *=* 38.1* Hz*), 141.8 (C^5^), 142.7 (CH), 148.2 (4-NO_2_-C_6_H_4_), 163.7 (C-S); **HRMS** (ESI+): calcd for C_19_H_16_F_3_N_6_O_2_S^+^, [M+H]^+^: 449.1002, found 449.0994.

**4-[(2-(*S*-methyl-carbonimidothioyl-hydrazinylidene)methyl]-3-trifluoromethyl-5-(4-fluorophenyl)-1-phenyl-*1H-*pyrazole** (**3b**): Beige solid; 78% yield (0.316 g); mp 145.5°C; **^1^H NMR** (300.06 MHz, CDCl_3_) δ (ppm) 2.45 (*s*, 3H, SCH_3_), 5.28 (*s*, 2H, NH_2_), 7.04–7.10 (*m*, 2H, 4-F-C_6_H_4_), 7.22-7.28 (*m*, 5H, 4-F-C_6_H_4_ and C_6_H_5_), 7.31–7.34 (*m*, 3H, C_6_H_5_), 8.22 (*s*, 1H, CH); **^13^C NMR** (75.45 MHz, CDCl_3_) δ (ppm) 12.8 (SCH_3_), 116.2 (C^4^), 116.2 (*d*, 4-F-C_6_H_4_, ^2^ *J*_C-F_ *=* 22.0* Hz*), 121.6 (*q*, CF_3_, ^1^ *J*_C-F_ *=* 269.7* Hz*), 124.7 (*d*, 4-F-C_6_H_4_, ^4^ *J*_C-F_ *=* 3.6* Hz*), 125.4, 128.7, 129.3 (C_6_H_5_), 132.5 (*d*, 4-F-C_6_H_4_, ^3^ *J*_C-F_ *=* 8.5* Hz*), 138.5 (C_6_H_5_), 140.8 (*q*, C^3^, ^2^ *J*_C-F_ *=* 38.0* Hz*), 143.5 (CH), 143.7 (C^5^), 163.2 (C-S), 163.3 (*d*, 4-F-C_6_H_4_, ^1^ *J*_C-F_ *=* 251.0* Hz*); **HRMS** (ESI+): calcd for C_19_H_16_F_4_N_5_S^+^, [M+H]^+^: 422.1057, found 422.1053.

**5-(4-chlorophenyl)-4-[(2-(*S*-methyl-carbonimidothioyl-hydrazinylidene)methyl]-3-trifluoromethyl-1-phenyl*-1H-*pyrazole** (**3c**): Yellow solid; 61% yield (0.257 g); mp 188.5°C; **^1^H NMR** (300.06 MHz, CDCl_3_) δ (ppm) 2.45 (*s*, 3H, SCH_3_), 5.22 (*s*, 2H, NH_2_), 7.21–7.24 (*m*, 2H, C_6_H_5_), 7.34–7.37 (*m*, 3H, C_6_H_5_), 7.48 (*d*, 2H, 4-Cl-C_6_H_4_, *J* = 8.9 Hz), 8.23 (*d*, 2H, 4-Cl-C_6_H_4_, *J* = 8.9 Hz), 8.25 (*s*, 1H, CH); **^13^C NMR** (75.45 MHz, DMSO-*d_6_*) δ (ppm) 12.8 (SCH_3_), 116.8 (C^4^), 121.4 (*q*, CF_3_, ^1^ *J*_C-F_ *=* 269.8* Hz*), 125.9 (C_6_H_5_), 126.3, 128.8 (4-Cl-C_6_H_4_), 129.2, 129.3 (C_6_H_5_), 132.4, 134.8 (4-Cl-C_6_H_4_), 133.0 (CH), 137.9 (C_6_H_5_), 138.2 (*q*, C^3^, ^2^ *J*_C-F_ *=* 37.3* Hz*), 143.9 (C^5^), 178.0 (C-S); **HRMS** (ESI+): calcd for C_19_H_16_ClF_3_N_5_S^+^, [M+H]^+^: 438.0762, found 438.0752.

**5-(4-Bromophenyl)-4-[(2-(*S*-methyl-carbonimidothioyl-hydrazinylidene)methyl]-3-trifluoromethyl-1-phenyl*-1H-*pyrazole** (**3d**): White solid; 80% yield (0.373 g); mp 137.4°C; **^1^H NMR** (300.06 MHz, CDCl_3_) δ (ppm) 2.45 (*s*, 3H, SCH_3_), 5.26 (*s*, 2H, NH_2_), 7.14 (*d*, 2H, 4-Br-C_6_H_4_, *J* = 8.6 Hz), 7.22–7.25 (*m*, 2H, C_6_H_5_), 7.32–7.36 (*m*, 3H, C_6_H_5_), 7.51 (*d*, 2H, 4-Br-C_6_H_4_, *J* = 8.6 Hz), 8.22 (*s*, 1H, CH); **^13^C NMR** (75.45 MHz, CDCl_3_) δ (ppm) 12.8 (SCH_3_), 116.2 (C^4^), 121.5 (*q*, CF_3_, ^1^ *J*_C-F_ *=* 269.6* Hz*), 124.1, 127.6 (4-Br-C_6_H_4_), 125.4, 128.7, 129.3 (C_6_H_5_), 132.0, 132.1 (4-Br-C_6_H_4_), 138.4 (C_6_H_5_), 140.8 (*q*, C^3^, ^2^ *J*_C-F_ *=* 37.9* Hz*), 143.3 (CH), 143.4 (C^5^), 163.3 (C-S); **HRMS** (ESI+): calcd for C_19_H_16_BrF_3_N_5_S^+^, [M+H]^+^: 482.0256, found 482.0242.

**4-[(2-(*S*-methyl-carbonimidothioyl-hydrazinylidene)methyl]-3-trifluoromethyl-1,5-diphenyl-*1H-*pyrazole** (**3e**): Beige solid; 73% yield (0.283 g); mp 165.65°C; **^1^H NMR** (300.06 MHz, CDCl_3_) δ (ppm) 2.44 (*s*, 3H, SCH_3_), 5.25 (*s*, 2H, NH_2_), 7.23–7.39 (*m*, 10H, C_6_H_5_ A and B), 8.24 (*s*, 1H, CH); **^13^C NMR** (75.45 MHz, CDCl_3_) δ (ppm) 12.8 (SCH_3_), 116.0 (C^4^), 121.6 (*q*, CF_3_, ^1^ *J*_C-F_ *=* 269.6* Hz*), 125.3, 128.5, 128.7, 128.8, 129.1, 129.5, 130.5, 138.7 (C_6_H_5_ – A and B), 140.6 (*q*, C^3^, ^2^ *J*_C-F_ *=* 37.9* Hz*), 143.7 (CH), 144.8 (C^5^), 163.1 (C-S); **HRMS** (ESI+): calcd for C_19_H_17_F_3_N_5_S^+^, [M+H]^+^: 404.1151, found 404.1140.

**4-[(2-(*S*-methyl-carbonimidothioyl-hydrazinylidene)methyl]-3-trifluoromethyl-5-(4-methoxyphenyl)-1-phenyl-*1H-*pyrazole** (**3f**): White solid; 68% yield (0.284 g); mp 126.2°C; **^1^H NMR** (300.06 MHz, CDCl_3_) δ (ppm) 2.45 (*s*, 3H, SCH_3_), 3.82 (*s*, 3H, 4-OCH_3_-C_6_H_4_), 5.31 (*s*, 2H, NH_2_), 6.88 (*d*, 2H, 4-OCH_3_-C_6_H_4_ *, J* = 8.2 Hz), 7.17 (*d*, 2H, 4-OCH_3_-C_6_H_4_ *, J* = 8.8 Hz), 7.24–7.33 (*m*, 5H, C_6_H_5_), 8.22 (*s*, 1H, CH); **^13^C NMR** (75.45 MHz, CDCl_3_) δ (ppm) 12.8 (SCH_3_), 55.4 (4-OCH_3_-C_6_H_4_), 114.3 (4-OCH_3_-C_6_H_4_), 115.8 (C^4^), 120.5 (4-OCH_3_-C_6_H_4_), 121.6 (*q*, CF_3_, ^1^ *J*_C-F_ *=* 269.6* Hz*), 125.4, 128.4, 129.1 (C_6_H_5_), 131.8 (4-OCH_3_-C_6_H_4_), 138.8 (C_6_H_5_), 140.4 (*q*, C^3^, ^2^ *J*_C-F_ *=* 37.8* Hz*), 144.0 (CH), 144.9 (C^5^), 160.5 (4-OCH_3_-C_6_H_4_), 162.9 (C-S); **HRMS** (ESI+): calcd for C_20_H_19_F_3_N_5_OS^+^, [M+H]^+^: 434.1257, found 434.1254.

#### Synthesis of 4-[(2-Amino)-1,3,4-Thiadiazol-5-yl]-5-Aryl-3-Trifluoromethyl-1-Phenyl-*1H*-Pyrazole (4a–f)

##### General Method

In a solution of thiosemicarbazone derivatives **2** (**2a**: 0.434 g; **2b**: 0.407 g; **2c**: 0.423 g; **2d**: 0.468 g; **2e**: 0.389 g; **2f**: 0.419, 1.0 mmol, 1.0 equiv.) in dioxane (5.0 ml) was added sodium carbonate (0.318 g, 3.0 mmol, 3.0 equiv.) and iodine (0.304 g, 1.2 mmol, 1.2 equiv.). The mixture was stirred under reflux for 4 h. Then, the solvent was evaporated under vacuum and the residue was washed with a solution of 6% of Na_2_S_2_O_3_ (25 ml), extracted with ethyl acetate (3 × 20 ml) and dried with anhydrous sodium sulfate. The obtained residue was dissolved in a mixture of hexane/ethyl acetate (4:1) and cooled to 0°C, which induced crystallization. The solid was filtered, washed with cold hexane (20 ml), and dried under vacuum.

**4-[(2-amino)-1,3,4-thiadiazo-5-yl]-3-trifluoromethyl-5-(4-nitrophenyl)-1-phenyl-*1H-*pyrazole** (**4a**): White solid; 56% yield (0.242 g); mp 236.7°C; **^1^H NMR** (500.13 MHz, DMSO-*d*_6_) δ (ppm) 7.34 (*s*, 2H, NH_2_), 7.42–7.45 (*m*, 5H, C_6_H_5_), 7.67 (*d*, 2H, 4-NO_2_-C_6_H_4_, *J* = 8.8 Hz), 8.24 (*d*, 2H, 4-NO_2_-C_6_H_4_, *J* = 8.8 Hz); **^13^C NMR** (125.76 MHz, DMSO-*d*_6_) δ (ppm) 111.7 (C^4^), 121.0 (*q*, CF_3_, ^1^ *J*_C-F_ *=* 269.7* Hz*), 123.6 (4-NO_2_-C_6_H_4_), 126.1, 129.3, 129.4 (C_6_H_5_), 132.4,133.8 (4-NO_2_-C_6_H_4_), 137.9 (C_6_H_5_), 139.1 (*q*, C^3^, ^2^ *J*_C-F_ *=* 37.0* Hz*), 142.5 (C^5^), 143.9 (C=N), 148.2 (4-NO_2_-C_6_H_4_), 169.8 (C-NH_2_); **HRMS** (ESI+): calcd for C_18_H_12_F_3_N_6_O_2_S^+^, [M+H]^+^: 433.0689, found 433.0704.

**4-[(2-amino)-1,3,4-thiadiazo-5-yl]-3-trifluoromethyl-5-(4-fluorophenyl)-1-phenyl-*1H-*pyrazole** (**4b**): White solid; 53% yield (0.245 g); mp 243.3°C; **^1^H NMR** (300.06 MHz, DMSO-*d*_6_) δ (ppm) 7.23–7.29 (*m*, 2H, 4-F-C_6_H_4_), 7.36–7.46 (*m*, 9H, 4-F-C_6_H_4_, C_6_H_5_ and NH_2_); **^13^C NMR** (75.45 MHz, DMSO-*d*_6_) δ (ppm) 112.2 (C^4^), 116.6 (*d*, 4-F-C_6_H_4_, ^2^ *J*_C-F_ *=* 21.9* Hz*), 121.7 (*q*, CF_3_, ^1^ *J*_C-F_ *=* 269.1* Hz*), 124.3 (*d*, 4-F-C_6_H_4_, ^4^ *J*_C-F_ *=* 3.1* Hz*), 126.6, 129.8, 129.8 (C_6_H_5_), 133.9 (*d*, 4-F-C_6_H_4_, ^3^ *J*_C-F_ *=* 8.5* Hz*), 138.7 (C_6_H_5_), 137.4 (*q*, C^3^, ^2^ *J*_C-F_ *=* 38.4* Hz*), 144.3 (C^5^), 163.6 (*d*, 4-F-C_6_H_4_, ^1^ *J*_C-F_ *=* 248.2* Hz*); **HRMS** (ESI+): calcd for C_18_H_12_F_4_N_5_S^+^, [M+H]^+^: 406.0744, found 406.0736.

**4-[(2-amino)-1,3,4-thiadiazo-5-yl]-5-(4-chlorophenyl)-3-trifluoromethyl-1-phenyl*-1H-*pyrazole** (**4c**): White solid; 54% yield (0.229 g); mp 224.9°C; **^1^H NMR** (300.06 MHz, DMSO-*d*_6_) δ (ppm) 7.33 (*s*, 2H, NH_2_), 7.39–7.44 (*m*, 7H, C_6_H_5_ and 4-Cl-C_6_H_4_), 7.49 (*d*, 2H, 4-Cl-C_6_H_4_, *J* = 8.5 Hz); **^13^C NMR** (75.45 MHz, DMSO-*d_6_*) δ (ppm) 111.4 (C^4^), 121.1 (*q*, CF_3_, ^1^ *J*_C-F_ *=* 269.6* Hz*), 126.0 (C_6_H_5_), 126.1 (4-Cl-C_6_H_4_), 128.9, 129.2 (C_6_H_5_), 129.2, 132.6, 135.1 (4-Cl-C_6_H_4_), 138.0 (C_6_H_5_), 138.7 (*q*, C^3^, ^2^ *J*_C-F_ *=* 36.8* Hz*), 143.3 (C^5^), 169.6 (C-NH_2_); **HRMS** (ESI+): calcd for C_18_H_12_ClF_3_N_5_S^+^, [M+H]^+^: 422.0449, found 422.0471.

**4-[(2-amino)-1,3,4-thiadiazo-5-yl]-5-(4-Bromophenyl)-3-trifluoromethyl-1-phenyl*-1H-*pyrazole** (**4d**): White solid; 54% yield (0.251 g); mp 239.2°C; **^1^H NMR** (300.06 MHz, DMSO-*d*_6_) δ (ppm) 7.31–7.33 (*m*, 4H, 4-Br-C_6_H_4_ and NH_2_), 7.37–7.45 (*m*, 5H, C_6_H_5_), 7.62 (*d*, 2H, 4-Br-C_6_H_4_, *J* = 8.4 Hz); **^13^C NMR** (75.45 MHz, DMSO-*d*_6_) δ (ppm) 111.4 (C^4^), 121.1 (*q*, CF_3_, ^1^ *J*_C-F_ *=* 269.7* Hz*), 124.0, 126.4 (4-Br-C_6_H_4_), 126.0, 126.4, 129.2 (C_6_H_5_), 131.8, 132.8 (4-Br-C_6_H_4_), 138.0 (C_6_H_5_), 138.8 (*q*, C^3^, ^2^ *J*_C-F_ *=* 37.0* Hz*), 143.3 (C^5^); **HRMS** (ESI+): calcd for C_18_H_12_BrF_3_N_5_S^+^, [M+H]^+^: 465.9943, found 465.9974.

**4-[(2-amino)-1,3,4-thiadiazo-5-yl]-3-trifluoromethyl-1,5-diphenyl-*1H-*pyrazole** (**4e**): White solid; 52% yield (0.201 g); mp 275.5; **^1^H NMR** (300.06 MHz, DMSO-*d*_6_) δ (ppm) 7.27 (*s*, 2H, NH_2_), 7.34–7.44 (*m*, 10H, C_6_H_5_ A and B); **^13^C NMR** (75.45 MHz, DMSO-*d*_6_) δ (ppm) 111.3 (C^4^), 121.1 (*q*, CF_3_, ^1^ *J*_C-F_ *=* 269.8* Hz*), 125.9, 127.1, 128.7, 129.1, 129.1, 130.1, 130.7, 138.2 (C_6_H_5_ – A and B), 138.7 (*q*, C^3^, ^2^ *J*_C-F_ *=* 36.9* Hz*), 144.5 (C^5^); **HRMS** (ESI+): calcd for C_18_H_13_F_3_N_5_S^+^, [M+H]^+^: 388.0838, found 388.0860.

**4-[(2-amino)-1,3,4-thiadiazo-5-yl]-3-trifluoromethyl-5-(4-methoxyphenyl)-1-phenyl-*1H-*pyrazole** (**4f**): White solid; 50% yield (0.208 g); mp 239.1°C; **^1^H NMR** (300.06 MHz, DMSO-*d*_6_) δ (ppm) 2.45 (*s*, 3H, SCH_3_), 3.75 (*s*, 3H, 4-OCH_3_-C_6_H_4_), 6.95 (*d*, 2H, 4-OCH_3_-C_6_H_4_ *, J* = 8.8 Hz), 7.25–7.28 (*m*, 4H, 4-OCH_3_-C_6_H_4_ and NH_2_), 7.35–7.43 (*m*, 5H, C_6_H_5_); **^13^C NMR** (75.45 MHz, DMSO-*d*_6_) δ (ppm) 55.2 (4-OCH_3_-C_6_H_4_), 111.2 (C^4^), 114.3 (4-OCH_3_-C_6_H_4_), 119.0 (4-OCH_3_-C_6_H_4_), 121.2 (*q*, CF_3_, ^1^ *J*_C-F_ *=* 269.6* Hz*), 125.9, 129.0, 129.2 (C_6_H_5_), 132.2 (4-OCH_3_-C_6_H_4_), 138.4 (C_6_H_5_), 139.1 (*q*, C^3^, ^2^ *J*_C-F_ *=* 36.5* Hz*), 144.5 (C^5^), 160.4 (4-OCH_3_-C_6_H_4_); **HRMS** (ESI+): calcd for C_19_H_15_F_3_N_5_OS^+^, [M+H]^+^: 418.0944, found 418.0934.

#### Synthesis of 5-Aryl–3-Trifluoromethyl-4-Formyl-1-Phenyl-*1H*-Pyrazole (5a)

##### General Method

The TBED **1a** (0.344** g**, 1.0 mmol, 1.0 equiv.) was solubilized in MeCN (8 ml), then added phenylhydrazine (0.108 g, 1.0 mmol, 1.0 equiv.) and boron trifluoride diethyl etherate solution 46.5% (0.400 ml, 1.5 mmol, 1.5 equiv.). The mixture was stirred under reflux for 7 h. Next, reaction mixture was cooled to room temperature and the solvent was evaporated under vacuum. Then, the residue was washed with distilled water (25 ml) extracted with dichloromethane (3 × 20 ml) and dried with anhydrous sodium sulfate. The solvent was evaporated under reduced pressure and the product was isolated on a silica gel chromatography column using a 95:5 mixture of hexane: ethyl acetate as the eluent.

**3-trifluoromethyl-4-formyl-5-(4-nitrophenyl)-1-phenyl-*1H-*pyrazole** (**5a**): Orange solid; 84% yield (0.311 g); mp 155.2–156.1°C; **^1^H NMR** (300.06 MHz, CDCl_3_) δ (ppm) 7.21–7.23 (*m*, 2H, C_6_H_5_) 7.38–7.44 (*m*, 3H, C_6_H_5_), 7.51 (*d*, 2H, 4-NO_2_-C_6_H_4_, *J* = 8.8 Hz), 8.25 (*d*, 2H, 4-NO_2_-C_6_H_4_, *J* = 8.8 Hz), 10.03 (*s*, 1H, CHO); **^13^C NMR** (75.45 MHz, CDCl_3_) δ (ppm) 119.0 (C^4^), 120.6 (*q*, CF_3_, ^1^ *J*_C-F_ *=* 270.6* Hz*), 123.9 (4-NO_2_-C_6_H_4_), 125.6, 129.7, 129.9, (C_6_H_5_), 131.8, 133.2 (4-NO_2_-C_6_H_4_), 137.4 (C_6_H_5_), 143.7 (*q*, C^3^, ^2^ *J*_C-F_ *=* 39.4* Hz*), 145.2 (C^5^), 148.8 (4-NO_2_-C_6_H_4_), 182.7 (CHO); **HRMS** (ESI+): calcd for C_17_H_11_F_3_N_3_O_3_^+^, [M+H]^+^: 362.0747, found 362.0755.

## Results and Discussion

**Figure 2:**
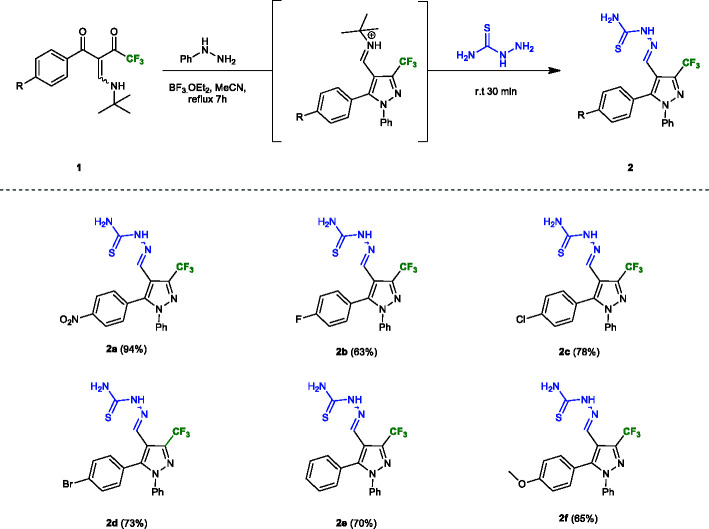
Substrate scope of thiosemicarbazones **2a–f**. Reaction conditions: TBED **1a–f** (1.0 mmol), phenylhydrazine (1.0 mmol), BF_3_OEt_2_ (1.5 mmol), MeCN (10.0 ml), thiosemicarbazide (3.0 mmol). Isolated yield after recrystallization in ethyl ether.

A one-pot synthesis of thiosemicarbazone derivatives **2a–f** starting from trifluoromethylated β-enamino diketone (TBED) **1a–f** was developed based on a methodology previously reported by our research group (Pianoski et al., 2020) (**Figure 2**). This reaction exhibited high regioselectivity, good scope, and efficiency, providing six new thiosemicarbazone derivatives (**2a–f**) in moderate to excellent yields (63–94%). The electronic effects of aryl group in the TBED displayed an important role, since aryl groups with electron withdrawing group had the best yields, while neutral and electron donors had the worst.

**Figure 3:**
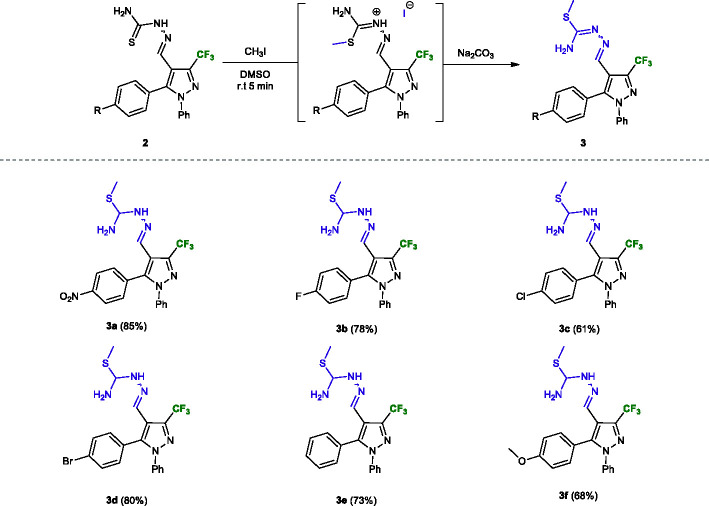
Substrate scope of thiosemicarbazones **3a–f**. Reaction conditions: Thiosemicarbazone derivatives **2a–f** (1.0 mmol), Na_2_CO_3_ (1.2 mmol), iodomethane (1.0 mmol), DMSO (5.0 ml). Isolated yield after recrystallization in hexane/ethyl acetate (4:1).

Next, we synthesized a series of *S*-methylated thiosemicarbazone derivatives **3a–f** from the methylation reaction of the sulfur atom of their respective thiosemicarbazones **2a–f**. For the synthesis of these derivatives, we performed some tests where the optimal condition was found when the reaction was carried out in DMSO, at room temperature, for 5 min, using 3.0 equiv. of iodomethane and 1.2 equivalents of Na_2_CO_3_. Satisfactorily, the methodology was simple and effective, and the products **3a–f** were obtained with moderate to good yields (61–85%) (**Figure 3**).

The syntheses of the 1,3,4-thiadiazole-pyrazole hybrids 4a–f were conducted from the oxidative cyclisation of their respective thiosemicarbazones 2a–f. Various methods have been reported for the synthesis of this core on literature (Hu et al., 2014; Haider et al., 2015), so we used two different oxidant agents and conditions for the cyclisation (**Table 1**). Initially, the compound 2a was subject to the oxidative cyclisation reaction using 1.2 equiv. of molecular iodine, 3.0 equivalents of Na_2_CO_3_, in THF, at room temperature. However, most of the starting material was recovered, even after prolonged reaction time (Entry 1, **Table 1**). On the other hand, when the reaction was carried out at reflux, the desired product 4a was obtained with a yield of 44% (Entry 2, **Table 1**). The yield of the product 4a was slightly improved when employed 1,4-dioxane reflux (Entry 3, **Table 1**). We also test ferric chloride as an oxidant. Nevertheless, a longer time was necessary for the total conversion to the desired product (Entries 4–6, **Table 1**). Thus, the best condition found for the oxidative cyclisation was using molecular iodine as oxidant with 3.0 equivalents of Na_2_CO_3_ under 1,4-dioxane reflux for 4** h** (Entry 3, **Table 1**).

**Table 1:** Optimization of the one-pot reaction to the synthesis of 4.

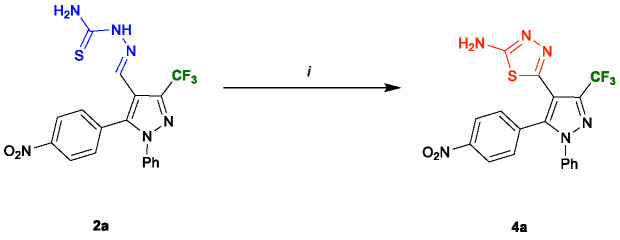								
Entry	Solvent	T °C	Na_2_CO_3_ (Eqv.)	Oxidant		Time (hour)	Conversion (%)^[a]^	Yield (%)^[b]^
				Oxidant	Eqv			
**1**	THF	r.t	3.0	I_2_	1.2	48	21.8	**–**
**2**	THF	Reflux	3.0	I_2_	1.2	4	100	44
**3**	1,4-dioxane	Reflux	3.0	I_2_	1.2	4	100	56
**4**	EtOH	Reflux	–	FeCl_3_	1.0	4	36	–
**5**	H_2_O/EtOH	Reflux	–	FeCl_3_	1.0	4	14.5	**–**
**6**	EtOH	Reflux	–	FeCl_3_	1.0	12	100	52

^[a]^Conversion determined from the ^1^H NMR spectrum of the crude product ^[b]^The product was dissolved in a mixture of hexane/ethyl acetate (4:1) and cooled to 0°C, which induced crystallization.

**Figure 4:**
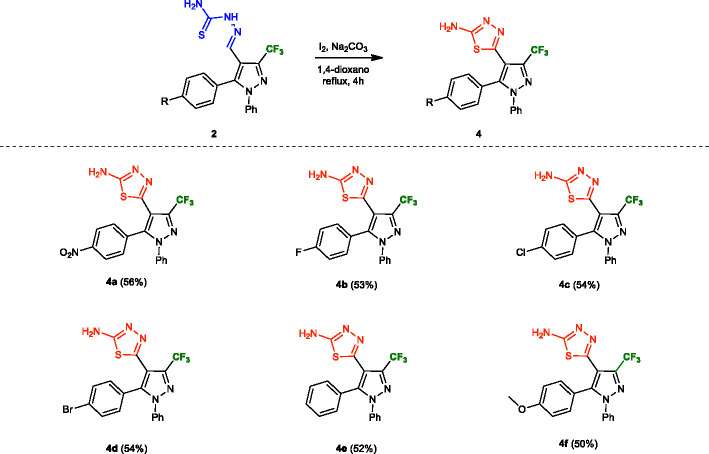
Substrate scope of thiosemicarbazones **4a–f**. Reaction conditions: Thiosemicarbazone derivatives **2a–f** (1.0 mmol), Na_2_CO_3_ (3.0 mmol), iodine (1.0 mmol), dioxane (5.0 ml). Isolated yield after recrystallization in hexane/ethyl acetate (4:1).

The efficacy of the methodology was demonstrated when applied for the other thiosemicarbazones 2b–f containing R groups with distinct electronic properties. In general, the properties of these groups did not affect the reaction, and the 2-amino-1,3,4-thiadiazole pyrazole hybrids 4a–f were obtained with yields from 50 to 56% (**Figure 4**).

Finally, all the structures of the synthesized compounds were elucidated based on ^1^H and ^13^C supplemented with 2D NMR measurements.

### Antiproliferative and Cytotoxic Activities

All the novel trifluoromethylated pyrazoles derivatives **2a–f**, **3a–f**, and **4a–f** were evaluated against the promastigote form of *L. amazonensis* and the epimastigote form of *T. cruzi*. The results are reported as the concentration causing a 50% inhibition in cell growth (IC_50_). Additionally, the toxicity of compounds was evaluated against two different cell lines: epithelial cell LLCMK_2_ and macrophages J774A1. The antiproliferative and cytotoxic activities are shown in **Table 2**, as well as the calculated selectivity index (SI). In general, 18 compounds showed antiproliferative activity against *L*. *amazonensis* and *T*. *cruzi*.

**Table 2:** *In vitro* antiproliferative activity in *L. amazonensis*, *T. cruzi* and cytotoxicity in mammalian cells treated with the compounds.

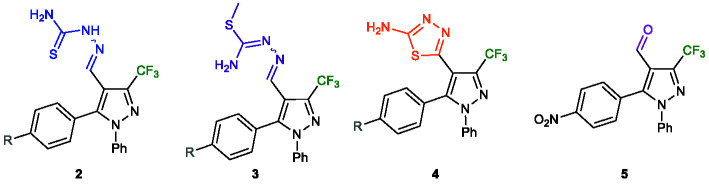									
Comp.	R	*L. amazonensis*IC_50_ (µM)*^[a]^*	*T. cruzi*IC_50_ (µM)*^[b]^*	Epithelial cellsCC_50_ (µM)*^[c]^*	MacrophagesCC_50_ (µM)*^[d]^*	SI_L-E_ *^[e]^*	SI_L-M_ *^[f]^*	SI_T-E_ *^[g]^*	SI_T-M_ *^[h]^*
**2a**	NO_2_	**26.7 ± 1.4**	45.7 ± 5.0	302.6 ± 5.8	243.1 ± 3.6	11.33	**9.10**	6.62	5.32
**2b**	F	49.6 ± 3.8	69.3 ± 2.9	345.7 ± 2.6	202.7 ± 5.9	6.97	4.09	4.99	2.92
**2c**	Cl	44.6 ± 6.2	49.2 ± 3.1	376.8 ± 5.0	402.2 ± 8.3	8.45	9.02	7.66	8.17
**2d**	Br	**18.9 ± 1.1**	30.3 ± 5.2	148.3 ± 6.9	169.7 ± 7.2	7.85	**8.98**	4.89	5.60
**2e**	H	61.7 ± 2.9	83.1 ± 5.3	394.4 ± 7.1	399.5 ± 6.6	6.39	6.47	4.75	4.81
**2f**	MeO	**22.4 ± 3.0**	29.7 ± 2.6	154.8 ± 2.6	180.1 ± 4.7	6.91	8.04	5.21	6.06
**3a**	NO_2_	**18.3 ± 2.4**	24.1 ± 0.8	176.4 ± 5.9	221.4 ± 3.1	9.64	**12.10**	7.32	9.19
**3b**	F	34.3 ± 4.7	39.9 ± 3.2	119.4 ± 3.0	202.4 ± 3.4	3.48	5.90	2.99	5.07
**3c**	Cl	30.7 ± 2.2	37.2 ± 4.8	297.1 ± 6.1	300.8 ± 3.9	9.68	9.80	7.99	8.09
**3d**	Br	**13.9 ± 0.6**	25.7 ± 2.5	114.0 ± 3.8	146.7 ± 3.6	8.20	**10.55**	4.44	5.71
**3e**	H	44.7 ± 5.0	62.4 ± 3.8	202.5 ± 6.2	326.4 ± 4.5	4.53	7.30	3.25	5.23
**3f**	MeO	**14.2 ± 1.1**	19.1 ± 0.9	101.3 ± 2.4	122.9 ± 3.1	7.13	**8.65**	5.30	6.43
**4a**	NO_2_	**19.6 ± 1.5**	29.0 ± 2.7	211.3 ± 3.1	285.3 ± 5.6	10.78	**14.56**	7.29	9.84
**4b**	F	40.4 ± 3.2	41.6 ± 5.3	146.5 ± 5.4	249.3 ± 6.2	3.63	6.17	3.52	5.99
**4c**	Cl	36.1 ± 2.8	40.5 ± 3.1	349.6 ± 6.1	385.2 ± 5.8	9.68	10.67	8.63	9.51
**4d**	Br	**14.7 ± 0.9**	26.7 ± 1.4	120.1 ± 2.5	147.1 ± 3.2	8.17	**10.01**	4.50	5.51
**4e**	H	52.4 ± 3.5	65.6 ± 2.9	264.3 ± 3.2	340.1 ± 5.2	5.04	6.49	4.03	5.18
**4f**	MeO	**17.1 ± 1.6**	22.8 ± 1.2	133.9 ± 5.1	156.3 ± 4.1	7.83	**9.14**	5.87	6.86
**5**	NO_2_	73.1 ± 2.8	80.8 ± 3.7	421.6 ± 12.3	244.9 ± 2.7	5.76	3.35	5.22	3.03

^[a]^IC_50_ values of promastigote form of Leishmania amazonensis expressed as the mean ± the standard deviation. ^[b]^IC_50_ values of epimastigote form of Trypanosoma cruzi expressed as the mean ± the standard deviation. ^[c]^Cytotoxic concentration corresponding to 50% inhibition of epithelial cells (LLCMK_2_) growth. ^[d]^Cytotoxic concentration corresponding to 50% inhibition of macrophages (J774A1) growth. ^[e]^Selectivity index (Epithelial CC_50_/L. amazonensis IC_50_). ^[f]^Selectivity index (Macrophages CC_50_/L. amazonensis IC_50_). ^[g]^Selectivity index (Epithelial CC_50_/T. cruzi IC_50_). ^[h]^Selectivity index (Macrophages CC_50_/T. cruzi IC_50_). The best results are in bold.

As shown in **Table 2**, the series of the compounds **2a–f** exhibited IC_50_ values in the range 18.9 to 61.7 µM against *L. amazonensis*. The unsubstituted phenyl ring led to the compound with the worst activity (**2e**, IC_50_ = 61.7 µM). Among the halogens, the *para*-bromo phenyl ring exhibited the best activity (**2d**, IC_50_ = 18.9 µM), and was also the most active compound among the series **2a–f**. The electron-withdrawing nitro group (**2a**, IC_50_ = 26.7 µM) and the electron-donating methoxy group (**2f**, IC_50_ = 22.4 µM) at the *para* position were also tolerated, presumably for steric reasons. In general, the compounds containing the bulkiest groups at the *para* position exhibited the best results against *L*. *amazonensis*. The selective index against macrophages ranged from 8 to 9.

For the series of compounds **3a–f**, again bulky groups such as bromo, methoxy, and nitro at the *para* position led to the most active compounds with IC_50_ values of 13.9, 14.2, and 18.3 µM, respectively. Furthermore, the methylation of the sulfur atom (**3a–f**) offered an improvement on activity, leading to compounds about 1.5-fold more active compared to **2a–f**. The most active compound, **3d**, exhibited a selectivity index of 10.55 against macrophages.

Among the series of the 2-amino-1,3,4-thiadiazole pyrazole hybrids **4a–f,** it was also found that the presence of bulky groups such as bromo, methoxy, and nitro at the *para* position led to the most active compounds with IC_50_ values of 14.7 µM (**4d**), 17.1 µM (**4f**), and 19.6 µM (**4a**), respectively. These compounds showed SI in the range 9.14 to 14.56. Moreover, the molecular hybridization of 2-amino-1,3,4-thiadiazole and pyrazole moieties offered the most potent compounds (**4a–f**) compared to their thiosemicarbazone analogues (**2a–f**), demonstrating the importance of the heterocyclic ring.

All compounds of the series **2a–f**, **3a–f**, and **4a–f** were less potent against *T*. *cruzi* compared to *L. amazonensis*. The most active compounds for *L*. *amazonensis* (**2d**, **3d,** and **4d**) were about 2-fold less potent for *T*. *cruzi*. Again, the compounds with bulky groups at the *para* position had the best IC_50_ values in each series.

Aiming to evaluate the influence of the thiosemicarbazone group attached at the 3-position of the pyrazole core (**2a**), we choose to assess the antileishmanial and antitrypanosomal activities of the 4-formyl pyrazole derivative **5a**. The insertion of the formyl group at the 4-position of the pyrazole ring led to a 3-fold and 2-fold decrease in activity against *L*. *amazonensis* and *T. cruzi*, respectively. Compound **5a** also exhibited a worse SI compared to **2a**.

It is worth mentioning that comparing the values IC_50_ of the series **2a–f** with **3a–f** and **4a–f** it is observed a low variance between the results obtained. In general, the compounds of the series **3a–f** were 1.4-fold most active compared to **2a–f**, and the compounds **4a–f** were 1.2-fold most active than **2a–f**. Comparing the works of Batista (Batista et al., 2019) and Vandresen (Vandresen et al., 2017), the transformation of the thiosemicarbazone group into 2-amino-1,3,4-thiadiazole derivative led to compounds in the range 1.4 to 6-fold less actives.

## Conclusions

In summary, we synthesized 18 new trifluormethylated pyrazole hybrids by a simple and efficient methodology. Screening of the compounds against *Leishmania amazonensis* and *Trypanosoma cruzi* demonstrated the importance of the substitution pattern of the trifluoromethylated *N-*aryl pyrazole system. The presence of a bulky group such as bromo, methoxy, or nitro at the *para* position of the aryl ring attached at the 5-position of pyrazole was essential for antiparasitic activity. Also, the nature of the substituent attached at the 3-position of pyrazole influenced the activity, as the thiosemicarbazone derivative led to active compounds, whereas the formyl derivative was inactive. Furthermore, the transformation of the thiosemicarbazone into *S-*methyl and 2-amino-1,3,4-thiadiazole derivatives increased the activity against both protozoans. However, the compounds were more active against *L*. *amazonensis*. From the results obtained in this study, the *S-*methyl thiosemicarbazones **3a**, **3d**, and **3f**, and 2-amino-1,3,4-thiadiazole pyrazole hybrids **4a**, **4d**, and **4f** could be considered as lead structures for the optimization of antileishmanial and antitrypanosomal properties.

## Data Availability Statement

All datasets presented in this study are included in the article/**Supplementary Material**.

## Author Contributions

JC and KP contributed to the synthesis, analysis of the results, and writing of the paper. MS contributed to synthesis and formal analysis. DL-B and HV contributed to pharmacological analyses. SM contributed to the HRMS analysis. CN contributed to the supervision of pharmacological analyses. FR contributed to designing the work, synthesis supervision, project administration, funding acquisition, and in the review and editing of the writing. All authors contributed to the article and approved the submitted version.

## Funding

Coordenação de Aperfeiçoamento de Pessoal de Nível Superior–CAPES/Brazil (AUXPE-PROEX-CAPES-Processo n° 23038.000872/2018-83).

## Conflict of Interest

The authors declare that the research was conducted in the absence of any commercial or financial relationships that could be construed as a potential conflict of interest.
