# Supplementary material for: Antiparasitic Behavior of Trifluoromethylated Pyrazole 2-Amino-1,3,4-thiadiazole Hybrids and Their Analogues: Synthesis and Structure-Activity Relationship
Source: Front Pharmacol. 2020 Oct 7;11:591570. doi: 10.3389/fphar.2020.591570 (PMC7576996; doi:10.3389/fphar.2020.591570)
Supplement: Supplementary file 1 [file DataSheet_1.pdf]

Antiparasitic behaviour of trifluoromethylated pyrazole 2-amino-1,3,4-thiadiazole hybrids and their analogues: synthesis and structure-activity relationship

Jeniffer do Nascimento Ascencio Camargo[1], Karlos Eduardo Pianoski[1], Mariellen Guilherme dos Santos[1], Danielle Lazarin-Bidóia[2], Hélito Volpato[2], Sidnei Moura[3], Celso Vataru Nakamura[2], Fernanda Andreia Rosa[1*]

[1]Departamento de Química, Universidade Estadual de Maringá (UEM), Maringá, PR, Brazil.

[2]Laboratório de Inovação Tecnológica no Desenvolvimento de Fármacos e Cosméticos, Departamento de Ciências Básicas da Saúde, Universidade Estadual de Maringá (UEM), 87020-900 Maringá-PR, Brazil.

[3]Instituto de Biotecnologia, Universidade de Caxias do Sul (UCS), Caxias do Sul, RS, Brazil.

*** Correspondence:**
Corresponding Author
farosa@uem.br

Supplementary Material

Table of contents

1. General Information, Synthetic Procedure and Spectra Data ..…………………………….…….S2-S7
2. ^{1}H and ^{13}C spectra for **2a-2f**…..S9-S20
3. ^{1}H and ^{13}C spectra for **3a-3f**...……..............................S21-S32
4. ^{1}H and ^{13}C spectra for **4a-4f**...……………………....................S32-S44
5. ^{1}H and ^{13}C spectra for **5a**...……………………......................S45-S46
6. References……………………………………………..…………………………...…….….….S47

1 General Information

The reagents used were obtain by commercial supplier without previous purification. Solvents were dried and purify according to recommend procedures[(1)]. The reactions were monitored by thin-layer chromatography with Merck TLC silica gel plates and analyzed with UV light. ^{1}H NMR, ^{13}C NMR, HSQC and HMBC experiments were run on Bruker avance III HD apparatus operating at ^{1}H 300 and 500 MHz and ^{13}C 75 and 125 MHz. Chemical shifts are reported in ppm using TMS as the internal standard for $CDCl_3$ in ^{1}H and ^{13}C. All melting points were measured using a MQAPF-307 Microquímica apparatus using benzoic acid as internal standard. ESI(+)-MS and tandem ESI(+)-MS/MS were acquired using a hybrid high-resolution and high accuracy microTof (Q-TOF) mass spectrometer (Bruker). For ESI(+)-MS, the energy for the collision induced dissociations (CDI) was optimised for each component. For data acquisition and processing, the Q-TOF-control data analysis software (Bruker Scientific) was used.

2 General Synthetic Procedure and Spectra Data

2.1 Synthesis of 5-aryl-4-[(2-carbamothioyl-hydrazinylidene)methyl]-3-trifluoromethyl-1-phenyl-*1H*-pyrazole (2a-2f)

General method. The trifluoromethylated β-enamino diketone[(2)] **1** (**1a**: 0.229 g; **1b**: 0.317 g; **1c**: 0.334 g; **1d**: 0.378 g; **1e**: 0.334 g; **1f**: 0.329, 1.0 mmol, 1.0 equiv) was solubilized in MeCN (10.0 mL), then added phenylhydrazine (0.108 g, 1.0 mmol, 1.0 equiv) and boron trifluoride diethyl etherate solution 46.5% (0.400 mL, 1.5 mmol, 1.5 equiv). The mixture was stirred under reflux for 7 h. In sequence, the reaction mixture was cooled to room temperature, added thiosemicarbazide (0.276 g, 3.0 mmol, 3.0 equiv.) and stirred for 30 min. Then, the solvent was evaporated under vacuum and the residue was washed with a solution of 3% of K_2CO_3 (25 mL), extracted with dichloromethane (3x20 mL) and dried with anhydrous sodium sulfate. The solvent was evaporated under reduced pressure and the obtained residue was dissolved in hot ethyl ether (5 mL) and cooled to 0 °C which induced crystallization. The solid was filtered, washed with cold ethyl ether (20 mL) and dried under vacuum.

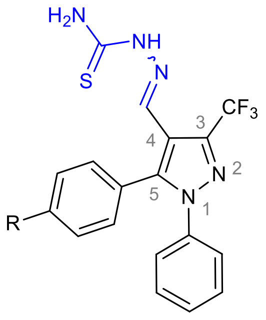

(**2a-2f**)

4-[(2-carbamothioyl-hydrazinylidene)methyl]-3-trifluoromethyl-5-(4-nitrophenyl)-1-phenyl-*1H*-pyrazole (**2a**): Yellow solid; 94% yield (0.408 g); mp 226.5 ºC; ^{1}**H NMR** (300.06 MHz, DMSO-d_6) δ (ppm) 6.49 (*s*, 1H, CSN<u>H</u>$_2$), 7.34-7.42 (*m*, 5H, C_6H_5), 7.69 (*d*, 2H, 4-NO_2-C_6H_4, J = 8.9 Hz), 8.01 (*s*, 1H, C<u>H</u>), 8.25 (*d*, 2H, 4-NO_2-C_6H_4, J = 8.9 Hz), 8.31 (*s*, 1H, CSN<u>H</u>$_2$), 11.50 (*s*, 1H, N<u>H</u>); ^{13}**C NMR** (75.45 MHz, DMSO-d_6) δ (ppm) 115.2 (C^4), 121.5 (*q*, <u>C</u>F_3, $^{1}J_{C\text{-}F}$ = 269.5 Hz), 123.8 (4-NO_2-C_6H_4), 126.0, 129.5, 129.5 (C_6H_5), 132.3 (4-NO_2-C_6H_4), 132.6 (<u>C</u>H), 134.3 (4-NO_2-C_6H_4), 137.8 (C_6H_5), 138.7 (*q*, C^3, $^{2}J_{C\text{-}F}$ = 37.4 Hz), 142.7 (C^5), 148.1 (4-NO_2-C_6H_4), 178.2 (C=S); **HRMS** (ESI+): calcd for $C_{18}H_{14}F_3N_6O_2S^+$, $[M+H]^+$: 435.0846, found 435.0864.

4-[(2-carbamothioyl-hydrazinylidene)methyl]-3-trifluoromethyl-5-(4-fluorophenyl)-1-phenyl-*1H*-pyrazole (**2b**): White solid; 63% yield (0.256 g); mp 221.18 ºC; **^{1}H NMR** (500.13 MHz, $CDCl_3$) δ (ppm) 6.23 (*s*, 1H, CSN$\underline{H}_2$), 6.87 (*ls*, 1H, CSN$\underline{H}_2$), 7.09-7.15 (*m*, 2H, 4-F-C_6H_4), 7.20-7.24 (*m*, 4H, 4-F-C_6H_4 and C_6H_5), 7.33-7.38 (*m*, 3H, C_6H_5), 7.70 (*s*, 1H, C$\underline{H}$), 9.61 (*s*, 1H, N$\underline{H}$); **^{13}C NMR** (125.76 MHz, $CDCl_3$) δ (ppm) 114.1 (C^4), 116.5 (*d*, 4-F-C_6H_4, $^{2}J_{C-F}$ = 22.0 Hz), 121.2 (*q*, $\underline{C}F_3$, $^{1}J_{C-F}$ = 269.9 Hz), 123.7 (*d*, 4-F-C_6H_4, $^{4}J_{C-F}$ = 3.6 Hz), 125.4, 129.0, 129.4 (C_6H_5), 132.3 (*d*, 4-F-C_6H_4, $^{3}J_{C-F}$ = 8.5 Hz), 133.7 ($\underline{C}$H), 138.2 (C_6H_5), 140.3 (*q*, C^3, $^{2}J_{C-F}$ = 38.2 Hz), 144.1 (C^5), 163.5 (*d*, 4-F-C_6H_4, $^{1}J_{C-F}$ = 252.1 Hz), 178.2 (C=S); **HRMS** (ESI+): calcd for $C_{18}H_{14}F_4N_5S^+$, $[M+H]^+$: 408.0901, found 408.0918.

4-[(2-carbamothioyl-hydrazinylidene)methyl]-5-(4-chlorophenyl)-3-trifluoromethyl-1-phenyl-*1H*-pyrazole (**2c**): White solid; 78% yield (0.330 g); mp 225.37 ºC; **^{1}H NMR** (500.13 MHz, DMSO-d_6) δ (ppm) 6.64 (*s*, 1H, CSN$\underline{H}_2$), 7.32-7.44 (*m*, 7H, 4-Cl-C_6H_4 and C_6H_5), 7.52 (*d*, 2H, 4-Cl-C_6H_4, *J* = 8.6 Hz), 7.97 (*s*, 1H, C$\underline{H}$), 8.43 (*s*, 1H, CSN$\underline{H}_2$), 11.51 (*s*, 1H, N$\underline{H}$); **^{13}C NMR** (125.76 MHz, DMSO-d_6) δ (ppm) 114.6 (C^4), 121.4 (*q*, $\underline{C}F_3$, $^{1}J_{C-F}$ = 269.2 Hz), 125.9 (C_6H_5), 126.3, 128.8 (4-Cl-C_6H_4), 129.2, 129.3 (C_6H_5), 132.4, 134.8 (4-Cl-C_6H_4), 133.0 ($\underline{C}$H), 137.9 (C_6H_5), 138.2 (*q*, C^3, $^{2}J_{C-F}$ = 37.3 Hz), 143.9 (C^5), 178.0 (C=S); **HRMS** (ESI+): calcd for $C_{18}H_{14}ClF_3N_5S^+$, $[M+H]^+$: 424.0605, found 424.0625.

5-(4-bromophenyl)-4-[(2-carbamothioyl-hydrazinylidene)methyl]-3-trifluoromethyl-1-phenyl-*1H*-pyrazole (**2d**): White solid; 73% yield (0.342 g); mp 226.12 ºC; **^{1}H NMR** (500.13 MHz, DMSO-d_6) δ (ppm) 6.65 (*s*, 1H, CSN$\underline{H}_2$), 7.32-7.36 (*m*, 4H, C_6H_5 and 4-Br-C_6H_4), 7.42-7.45 (*m*, 3H, C_6H_5), 7.65 (*d*, 2H, 4-Br-C_6H_4, *J* = 8.5 Hz), 7.97 (*s*, 1H, C$\underline{H}$), 8.43 (*s*, 1H, CSN$\underline{H}_2$), 11.50 (*s*, 1H, N$\underline{H}$); **^{13}C NMR** (125.76 MHz, DMSO-d_6) δ (ppm) 114.6 (C^4), 121.4 (*q*, $\underline{C}F_3$, $^{1}J_{C-F}$ = 269.3 Hz), 125.8, 126.6 (4-Br-C_6H_4), 129.2, 129.3 (C_6H_5), 129.3, 131.7 (4-Br-C_6H_4), 132.6 (C_6H_5), 131.9 (4-Br-C_6H_4), 133.0 ($\underline{C}$H), 137.9 (C_6H_5), 138.2 (*q*, C^3, $^{2}J_{C-F}$ = 37.3 Hz), 143.9 (C^5), 178.0 (C=S); **HRMS** (ESI+): calcd for $C_{18}H_{14}BrF_3N_5S^+$, $[M+H]^+$: 468.0100, found 468.0102.

4-[(2-carbamothioyl-hydrazinylidene)methyl]-3-trifluoromethyl-1,5-diphenyl-*1H*-pyrazole (**2e**): White solid; 70% yield (0.272 g); mp 211.27 ºC; **^{1}H NMR** (500.13 MHz, DMSO-d_6) δ (ppm) 6.44 (*s*, 1H, CSN$\underline{H}_2$), 6.84 (*s*, 1H, CSN$\underline{H}_2$), *7*.19-7.41 (*m*, 10H, C_6H_5 A and B), 7.79 (*s*, 1H, C$\underline{H}$), 10.24 (*s*, 1H, N$\underline{H}$); **^{13}C NMR** (125.76 MHz, DMSO-d_6) δ (ppm) 114.0 (C^4), 121.3 (*q*, $\underline{C}F_3$, $^{1}J_{C-F}$ = 270.0 Hz), 127.8, 128.8, 129.1, 129.2, 130.1, 130.2, 138.4 (C_6H_5 – A and B), 133.9 ($\underline{C}$H), 140.3 (*q*, C^3, $^{2}J_{C-F}$ = 38.4 Hz), 145.1 (C^5), 178.0 (C=S); **HRMS** (ESI+): calcd for $C_{18}H_{15}F_3N_5S^+$, $[M+H]^+$: 390.0995, found 390.1002.

4-[(2-carbamothioyl-hydrazinylidene)methyl]-3-trifluoromethyl-5-(4-methoxyphenyl)-1-phenyl-*1H*-pyrazole (**2f**): White solid; 63% yield (0.272 g); mp 212.65 ºC; **^{1}H NMR** (500.13 MHz, $CDCl_3$) δ (ppm) 3.83 (*s*, 3H, 4-OC$\underline{H}_3$-C_6H_4), 6.39 (*s*, 1H, CSN$\underline{H}_2$), 6.91 (*d*, 2H, 4-OCH$_3$-C_6H_4, *J* = 8.8 Hz), 6.95 (*s*, 1H, CSN$\underline{H}_2$), 7.12 (*d*, 2H, 4-OCH$_3$-C_6H_4, *J* = 8.8 Hz), 7.23-7.34 (*m*, 5H, C_6H_5), 7.76 (*s*, 1H, C$\underline{H}$), 9.97 (*s*, 1H, N$\underline{H}$); **^{13}C NMR** (125.76 MHz, $CDCl_3$) δ (ppm) 55.5 (4-O$\underline{C}$H$_3$-C_6H_4), 113.7 (C^4), 114.6, 119.6 (4-OCH$_3$-C_6H_4), 121.4 (*q*, $\underline{C}F_3$, $^{1}J_{C-F}$ = 269.7 Hz), 125.3, 128.7, 129.3 (C_6H_5), 131.7 (4-O$\underline{C}$H$_3$-C_6H_4), 134.0 ($\underline{C}$H), 138.5 (C_6H_5), 140.2 (*q*, C^3, $^{2}J_{C-F}$ = 38.2 Hz), 145.3 (C^5), 160.8 (4-OCH$_3$-C_6H_4), 178.0 (C=S); **HRMS** (ESI+): calcd for $C_{19}H_{17}F_3N_5OS^+$, $[M+H]^+$: 420.1100, found 420.1121.

2.2 Synthesis of 5-aryl-4-[(2-(*S*-methyl-carbonimidothioyl-hydrazinylidene)methyl]-3-trifluoromethyl-1-phenyl-1H-pyrazole (3a-f)

General method. In a solution of thiosemicarbazone derivatives **2** (**2a**: 0.434 g; **2b**: 0.407 g; **2c**: 0.423 g; **2d**: 0.468 g; **2e**: 0.389 g; **2f**: 0.419, 1.0 mmol, 1.0 equiv) in DMSO (5.0 mL) was added sodium carbonate (0.127 g, 1.2 mmol, 1.2 equiv) and iodomethane solution 99% (0.063 mL, 1.0 mmol, 1.0 equiv). The mixture was stirred under room temperature for 5 min. Then, the product was washed with distilled water (5x15.0 mL) and filtered under vacuum. The obtained residue was dissolved in a mixture of hexane/ethyl acetate (4:1) and cooled to 0 °C, which induced crystallization. The solid was filtered, washed with cold hexane (20 mL) and dried under vacuum.

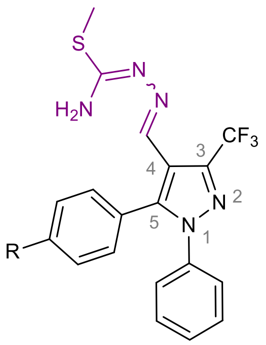

(**3a-3f**)

4-[(2-(*S*-methyl-carbonimidothioyl-hydrazinylidene)methyl]-3-trifluoromethyl-5-(4-nitrophenyl)-1-phenyl-*1H*-pyrazole (**3a**): Yellow solid; 85% yield (0.367 g); mp 190.7 ºC; **^{1}H NMR** (300.06 MHz, $CDCl_3$) δ (ppm) 2.45 (*s*, 3H, $SC\underline{H}_3$), 5.22 (*s*, 2H, $N\underline{H}_2$), 7.21-7.24 (*m*, 2H, C_6H_5), 7.34-7.37 (*m*, 3H, C_6H_5), 7.48 (*d*, 2H, 4-NO_2-C_6H_4, J = 8.9 Hz), 8.21-8.24 (*m*, 3H, 4-NO_2-C_6H_4 and $C\underline{H}$); **^{13}C NMR** (75.45 MHz, $CDCl_3$) δ (ppm) 12.8 ($S\underline{C}H_3$), 116.8 (C^4), 121.3 (*q*, $\underline{C}F_3$, $^1J_{C\text{-}F}$ = 269.8 Hz), 123.9 (4-NO_2-C_6H_4), 125.5, 129.2, 129.5 (C_6H_5), 131.6, 135.2 (4-NO_2-C_6H_4), 138.1 (C_6H_5), 141.2 (*q*, C^3, $^2J_{C\text{-}F}$ = 38.1 Hz), 141.8 (C^5), 142.7 ($\underline{C}H$), 148.2 (4-NO_2-C_6H_4), 163.7 ($\underline{C}$-S); **HRMS** (ESI+): calcd for $C_{19}H_{16}F_3N_6O_2S^+$, $[M+H]^+$: 449.1002, found 449.0994.

4-[(2-(*S*-methyl-carbonimidothioyl-hydrazinylidene)methyl]-3-trifluoromethyl-5-(4-fluorophenyl)-1-phenyl-*1H*-pyrazole (**3b**): Beige solid; 78% yield (0.316 g); mp 145.5 °C; **^{1}H NMR** (300.06 MHz, $CDCl_3$) δ (ppm) 2.45 (*s*, 3H, $SC\underline{H}_3$), 5.28 (*s*, 2H, $N\underline{H}_2$), 7.04-7.10 (*m*, 2H, 4-F-C_6H_4), 7.22-7.28 (*m*, 5H, 4-F-C_6H_4 and C_6H_5), 7.31-7.34 (*m*, 3H, C_6H_5), 8.22 (*s*, 1H, $C\underline{H}$); **^{13}C NMR** (75.45 MHz, $CDCl_3$) δ (ppm) 12.8 ($S\underline{C}H_3$), 116.2 (C^4), 116.2 (*d*, 4-F-C_6H_4, $^2J_{C\text{-}F}$ = 22.0 Hz), 121.6 (*q*, $\underline{C}F_3$, $^1J_{C\text{-}F}$ = 269.7 Hz), 124.7 (*d*, 4-F-C_6H_4, $^4J_{C\text{-}F}$ = 3.6 Hz), 125.4, 128.7, 129.3 (C_6H_5), 132.5 (*d*, 4-F-C_6H_4, $^3J_{C\text{-}F}$ = 8.5 Hz), 138.5 (C_6H_5), 140.8 (*q*, C^3, $^2J_{C\text{-}F}$ = 38.0 Hz), 143.5 ($\underline{C}H$), 143.7 (C^5), 163.2 (C-S), 163.3 (*d*, 4-F-C_6H_4, $^1J_{C\text{-}F}$ = 251.0 Hz); **HRMS** (ESI+): calcd for $C_{19}H_{16}F_4N_5S^+$, $[M+H]^+$: 422.1057, found 422.1053.

5-(4-chlorophenyl)-4-[(2-(*S*-methyl-carbonimidothioyl-hydrazinylidene)methyl]-3-trifluoromethyl-1-phenyl-*1H*-pyrazole (**3c**): Yellow solid; 61% yield (0.257 g); mp 188.5 ºC; **^{1}H NMR** (300.06 MHz, $CDCl_3$) δ (ppm) 2.45 (*s*, 3H, $SC\underline{H}_3$), 5.22 (*s*, 2H, $N\underline{H}_2$), 7.21-7.24 (*m*, 2H, C_6H_5), 7.34-7.37 (*m*, 3H, C_6H_5), 7.48 (*d*, 2H, 4-Cl-C_6H_4, J = 8.9 Hz), 8.23 (*d*, 2H, 4-Cl-C_6H_4, J = 8.9 Hz), 8.25 (*s*, 1H, $C\underline{H}$); **^{13}C NMR** (75.45 MHz, DMSO-d_6) δ (ppm) 12.8 ($S\underline{C}H_3$), 116.8 (C^4), 121.4 (*q*, $\underline{C}F_3$, $^1J_{C\text{-}F}$ = 269.8 Hz), 125.9 (C_6H_5), 126.3, 128.8 (4-Cl-C_6H_4), 129.2, 129.3 (C_6H_5), 132.4, 134.8 (4-Cl-C_6H_4), 133.0 ($\underline{C}H$), 137.9 (C_6H_5), 138.2 (*q*, C^3, $^2J_{C\text{-}F}$ = 37.3 Hz), 143.9 (C^5), 178.0 (C-S); **HRMS** (ESI+): calcd for $C_{19}H_{16}ClF_3N_5S^+$, $[M+H]^+$: 438.0762, found 438.0752.

5-(4-Bromophenyl)-4-[(2-(*S*-methyl-carbonimidothioyl-hydrazinylidene)methyl]-3-trifluoromethyl-1-phenyl-*1H*-pyrazole (**3d**): White solid; 80% yield (0.373 g); mp 137.4 ºC; **^{1}H NMR** (300.06 MHz, $CDCl_3$) δ (ppm) 2.45 (*s*, 3H, $SC\underline{H}_3$), 5.26 (*s*, 2H, $N\underline{H}_2$), 7.14 (*d*, 2H, 4-Br-C_6H_4, J = 8.6 Hz), 7.22-7.25 (*m*, 2H, C_6H_5), 7.32-7.36 (*m*, 3H, C_6H_5), 7.51 (*d*, 2H, 4-Br-C_6H_4, J = 8.6 Hz), 8.22 (*s*, 1H, $C\underline{H}$); **^{13}C NMR** (75.45 MHz, $CDCl_3$) δ (ppm) 12.8 ($S\underline{C}H_3$), 116.2 (C^4), 121.5 (*q*, $\underline{C}F_3$, $^1J_{C\text{-}F}$ = 269.6 Hz), 124.1, 127.6 (4-Br-C_6H_4), 125.4, 128.7, 129.3 (C_6H_5), 132.0, 132.1 (4-Br-C_6H_4), 138.4 (C_6H_5), 140.8 (*q*, C^3, $^2J_{C\text{-}F}$ = 37.9 Hz), 143.3 ($\underline{C}H$), 143.4 (C^5), 163.3 (C-S); **HRMS** (ESI+): calcd for $C_{19}H_{16}BrF_3N_5S^+$, $[M+H]^+$: 482.0256, found 482.0242.

4-[(2-(*S*-methyl-carbonimidothioyl-hydrazinylidene)methyl]-3-trifluoromethyl-1,5-diphenyl-*1H*-pyrazole (**3e**): Beige solid; 73% yield (0.283 g); mp 165.65 ºC; **^{1}H NMR** (300.06 MHz, $CDCl_3$) δ (ppm) 2.44 (*s*, 3H, $SC\underline{H}_3$), 5.25 (*s*, 2H, $N\underline{H}_2$), 7.23-7.39 (*m*, 10H, C_6H_5 A and B), 8.24 (*s*, 1H, $C\underline{H}$); **^{13}C NMR** (75.45 MHz, $CDCl_3$) δ (ppm) 12.8 ($S\underline{C}H_3$), 116.0 (C^4), 121.6 (*q*, $\underline{C}F_3$, $^1J_{C\text{-}F}$ = 269.6 Hz), 125.3, 128.5, 128.7, 128.8, 129.1, 129.5, 130.5, 138.7 (C_6H_5 – A and B), 140.6 (*q*, C^3, $^2J_{C\text{-}F}$ = 37.9 Hz), 143.7 ($\underline{C}H$), 144.8 (C^5), 163.1 (C-S); **HRMS** (ESI+): calcd for $C_{19}H_{17}F_3N_5S^+$, $[M+H]^+$: 404.1151, found 404.1140.

4-[(2-(*S*-methyl-carbonimidothioyl-hydrazinylidene)methyl]-3-trifluoromethyl-5-(4-methoxyphenyl)-1-phenyl-*1H*-pyrazole (**3f**): White solid; 68% yield (0.284 g); mp 126.2 ºC; **^{1}H NMR** (300.06 MHz, $CDCl_3$) δ (ppm) 2.45 (*s*, 3H, SC$\underline{H}_3$), 3.82 (*s*, 3H, 4-OC$\underline{H}_3$-C_6H_4), 5.31 (*s*, 2H, N$\underline{H}_2$), 6.88 (*d*, 2H, 4-OCH_3-C_6H_4, *J* = 8.2 Hz), 7.17 (*d*, 2H, 4-OCH_3-C_6H_4, *J* = 8.8 Hz), 7.24-7.33 (*m*, 5H, C_6H_5), 8.22 (*s*, 1H, C$\underline{H}$); **^{13}C NMR** (75.45 MHz, $CDCl_3$) δ (ppm) 12.8 (S$\underline{C}H_3$), 55.4 (4-O$\underline{C}H_3$-C_6H_4), 114.3 (4-OCH_3-C_6H_4), 115.8 (C^4), 120.5 (4-OCH_3-C_6H_4), 121.6 (*q*, $\underline{C}F_3$, $^1J_{C\text{-}F}$ = 269.6 Hz), 125.4, 128.4, 129.1 (C_6H_5), 131.8 (4-O$\underline{C}H_3$-C_6H_4), 138.8 (C_6H_5), 140.4 (*q*, C^3, $^2J_{C\text{-}F}$ = 37.8 Hz), 144.0 ($\underline{C}$H), 144.9 (C^5), 160.5 (4-OCH_3-C_6H_4), 162.9 (C-S); **HRMS** (ESI+): calcd for $C_{20}H_{19}F_3N_5OS^+$, $[M+H]^+$: 434.1257, found 434.1254.

2.3 Synthesis of 4-[(2-amino)-1,3,4-thiadiazo-5-yl]-5-aryl--3-trifluoromethyl-1-phenyl-*1H*-pyrazole (4a-4f)

General method. In a solution of thiosemicarbazone derivatives **2** (**2a**: 0.434 g; **2b**: 0.407 g; **2c**: 0.423 g; **2d**: 0.468 g; **2e**: 0.389 g; **2f**: 0.419, 1.0 mmol, 1.0 equiv) in dioxane (5.0 mL) was added sodium carbonate (0.318 g, 3.0 mmol, 3.0 equiv) and iodine (0.304 g, 1.2 mmol, 1.2 equiv). The mixture was stirred under reflux for 4 hours, according to the methodology described by Niu *et. al.*[(3)]. Then, the solvent was evaporated under vacuum and the residue was washed with a solution of 6% of $Na_2S_2O_3$ (25 mL), extracted with ethyl acetate (3x20 mL) and dried with anhydrous sodium sulfate. The obtained residue was dissolved in a mixture of hexane/ethyl acetate (4:1) and cooled to 0 °C, which induced crystallization. The solid was filtered, washed with cold hexane (20 mL) and dried under vacuum.

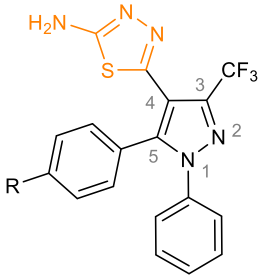

(4a-4f)

4-[(2-amino)-1,3,4-thiadiazo-5-yl]-3-trifluoromethyl-5-(4-nitrophenyl)-1-phenyl-*1H*-pyrazole (**4a**): White solid; 56% yield (0.242 g); mp 236.7 ºC; **^{1}H NMR** (500.13 MHz, DMSO-*d*$_6$) δ (ppm) 7.34 (*s*, 2H, NH_2), 7.42-7.45 (*m*, 5H, C_6H_5), 7.67 (*d*, 2H, 4-NO_2-C_6H_4, *J* = 8.8 Hz), 8.24 (*d*, 2H, 4-NO_2-C_6H_4, *J* = 8.8 Hz); **^{13}C NMR** (125.76 MHz, DMSO-*d*$_6$) δ (ppm) 111.7 (C^4), 121.0 (*q*, $\underline{C}F_3$, $^1J_{C\text{-}F}$ = 269.7 Hz), 123.6 (4-NO_2-C_6H_4), 126.1, 129.3, 129.4 (C_6H_5), 132.4,133.8 (4-NO_2-C_6H_4), 137.9 (C_6H_5), 139.1 (*q*, C^3, $^2J_{C\text{-}F}$ = 37.0 Hz), 142.5 (C^5), 143.9 ($\underline{C}$=N), 148.2 (4-NO_2-C_6H_4), 169.8 ($\underline{C}$-NH_2); **HRMS** (ESI+): calcd for $C_{18}H_{12}F_3N_6O_2S^+$, $[M+H]^+$: 433.0689, found 433.0704.

4-[(2-amino)-1,3,4-thiadiazo-5-yl]-3-trifluoromethyl-5-(4-fluorophenyl)-1-phenyl-*1H*-pyrazole (**4b**): White solid; 53% yield (0.245 g); mp 243.3 ºC; **^{1}H NMR** (300.06 MHz, DMSO-*d*$_6$) δ (ppm) 7.23-7.29 (*m*, 2H, 4-F-C_6H_4), 7.36-7.46 (*m*, 9H, 4-F-C_6H_4, C_6H_5 and N$\underline{H}_2$); **^{13}C NMR** (75.45 MHz, DMSO-*d*$_6$) δ (ppm) 112.2 (C^4), 116.6 (*d*, 4-F-C_6H_4, $^2J_{C\text{-}F}$ = 21.9 Hz), 121.7 (*q*, $\underline{C}F_3$, $^1J_{C\text{-}F}$ = 269.1 Hz), 124.3 (*d*, 4-F-C_6H_4, $^4J_{C\text{-}F}$ = 3.1 Hz), 126.6, 129.8, 129.8 (C_6H_5), 133.9 (*d*, 4-F-C_6H_4, $^3J_{C\text{-}F}$ = 8.5 Hz), 138.7 (C_6H_5), 137.4 (*q*, C^3, $^2J_{C\text{-}F}$ = 38.4 Hz), 144.3 (C^5), 163.6 (*d*, 4-F-C_6H_4, $^1J_{C\text{-}F}$ = 248.2 Hz); **HRMS** (ESI+): calcd for $C_{18}H_{12}F_4N_5S^+$, $[M+H]^+$: 406.0744, found 406.0736.

4-[(2-amino)-1,3,4-thiadiazo-5-yl]-5-(4-chlorophenyl)-3-trifluoromethyl-1-phenyl-*1H*-pyrazole (**4c**): White solid; 54% yield (0.229 g); mp 224.9 ºC; **^{1}H NMR** (300.06 MHz, DMSO-*d*$_6$) δ (ppm) 7.33 (*s*, 2H, N$\underline{H}_2$), 7.39-7.44 (*m*, 7H, C_6H_5 and 4-Cl-C_6H_4), 7.49 (*d*, 2H, 4-Cl-C_6H_4, *J* = 8.5 Hz); **^{13}C NMR** (75.45 MHz, DMSO-*d*$_6$) δ (ppm) 111.4 (C^4), 121.1 (*q*, $\underline{C}F_3$, $^1J_{C\text{-}F}$ = 269.6 Hz), 126.0 (C_6H_5), 126.1 (4-Cl-C_6H_4), 128.9, 129.2 (C_6H_5), 129.2,

132.6, 135.1 (4-Cl-C_6H_4), 138.0 (C_6H_5), 138.7 (*q*, C^3, $^2J_{C-F}$ = 36.8 Hz), 143.3 (C^5), 169.6 ($\underline{C}$-NH_2); **HRMS** (ESI+): calcd for $C_{18}H_{12}ClF_3N_5S^+$, $[M+H]^+$: 422.0449, found 422.0471.

4-[(2-amino)-1,3,4-thiadiazo-5-yl]-5-(4-Bromophenyl)-3-trifluoromethyl-1-phenyl-*1H*-pyrazole (**4d**): White solid; 54% yield (0.251 g); mp 239.2 ºC; **^{1}H NMR** (300.06 MHz, DMSO-d_6) δ (ppm) 7.31-7.33 (*m*, 4H, 4-Br-C_6H_4 and $N\underline{H}_2$), 7.37-7.45 (*m*, 5H, C_6H_5), 7.62 (*d*, 2H, 4-Br-C_6H_4, *J* = 8.4 Hz); **^{13}C NMR** (75.45 MHz, DMSO-d_6) δ (ppm) 111.4 (C^4), 121.1 (*q*, $\underline{C}F_3$, $^1J_{C-F}$ = 269.7 Hz), 124.0, 126.4 (4-Br-C_6H_4), 126.0, 126.4, 129.2 (C_6H_5), 131.8, 132.8 (4-Br-C_6H_4), 138.0 (C_6H_5), 138.8 (*q*, C^3, $^2J_{C-F}$ = 37.0 Hz), 143.3 (C^5); **HRMS** (ESI+): calcd for $C_{18}H_{12}BrF_3N_5S^+$, $[M+H]^+$: 465.9943, found 465.9974.

4-[(2-amino)-1,3,4-thiadiazo-5-yl]-3-trifluoromethyl-1,5-diphenyl-*1H*-pyrazole (**4e**): White solid; 52% yield (0.201 g); mp 275.5; **^{1}H NMR** (300.06 MHz, DMSO-d_6) δ (ppm) 7.27 (*s*, 2H, $N\underline{H}_2$), 7.34-7.44 (*m*, 10H, C_6H_5 A and B); **^{13}C NMR** (75.45 MHz, DMSO-d_6) δ (ppm) 111.3 (C^4), 121.1 (*q*, $\underline{C}F_3$, $^1J_{C-F}$ = 269.8 Hz), 125.9, 127.1, 128.7, 129.1, 129.1, 130.1, 130.7, 138.2 (C_6H_5 – A and B), 138.7 (*q*, C^3, $^2J_{C-F}$ = 36.9 Hz), 144.5 (C^5); **HRMS** (ESI+): calcd for $C_{18}H_{13}F_3N_5S^+$, $[M+H]^+$: 388.0838, found 388.0860.

4-[(2-amino)-1,3,4-thiadiazo-5-yl]-3-trifluoromethyl-5-(4-methoxyphenyl)-1-phenyl-*1H*-pyrazole (**4f**): White solid; 50% yield (0.208 g); mp 239.1 ºC; **^{1}H NMR** (300.06 MHz, DMSO-d_6) δ (ppm) 2.45 (*s*, 3H, $SC\underline{H}_3$), 3.75 (*s*, 3H, 4-$OC\underline{H}_3$-C_6H_4), 6.95 (*d*, 2H, 4-OCH_3-C_6H_4, *J* = 8.8 Hz), 7.25-7.28 (*m*, 4H, 4-OCH_3-C_6H_4 and $N\underline{H}_2$), 7.35-7.43 (*m*, 5H, C_6H_5); **^{13}C NMR** (75.45 MHz, DMSO-d_6) δ (ppm) 55.2 (4-$O\underline{C}H_3$-C_6H_4), 111.2 (C^4), 114.3 (4-OCH_3-C_6H_4), 119.0 (4-OCH_3-C_6H_4), 121.2 (*q*, $\underline{C}F_3$, $^1J_{C-F}$ = 269.6 Hz), 125.9, 129.0, 129.2 (C_6H_5), 132.2 (4-$O\underline{C}H_3$-C_6H_4), 138.4 (C_6H_5), 139.1 (*q*, C^3, $^2J_{C-F}$ = 36.5 Hz), 144.5 (C^5), 160.4 (4-OCH_3-C_6H_4); **HRMS** (ESI+): calcd for $C_{19}H_{15}F_3N_5OS^+$, $[M+H]^+$: 418.0944, found 418.0934.

2.4 Synthesis of 5-aryl--3-trifluoromethyl-4-formyl-1-phenyl-*1H*-pyrazole (5a)

General method. The TBED **1a** (0.344 g, 1.0 mmol, 1.0 equiv) was solubilized in MeCN (8.0 mL), then added phenylhydrazine (0.108 g, 1.0 mmol, 1.0 equiv) and boron trifluoride diethyl etherate solution 46.5% (0.400 mL, 1.5 mmol, 1.5 equiv). The mixture was stirred under reflux for 7 h. Next, reaction mixture was cooled to room temperature and the solvent was evaporated under vacuum. Then, the residue was washed with distilled water (25 mL) extracted with dichloromethane (3x20 mL) and dried with anhydrous sodium sulfate. The solvent was evaporated under reduced pressure and the product was isolated on a silica gel chromatography column using a 95:5 mixture of hexane: ethyl acetate as the eluent.

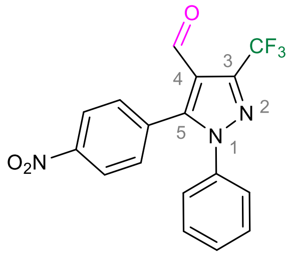

(**5a**)

3-trifluoromethyl-4-formyl-5-(4-nitrophenyl)-1-phenyl-*1H*-pyrazole (**5a**): Orange solid; 84% yield (0.311 g); mp 155.2-156.1 ºC; **^{1}H NMR** (300.06 MHz, $CDCl_3$) δ (ppm) 7.21-7.23 (*m*, 2H, C_6H_5) 7.38-7.44 (*m*, 3H, C_6H_5), 7.51 (*d*, 2H, 4-NO_2-C_6H_4, *J* = 8.8 Hz), 8.25 (*d*, 2H, 4-NO_2-C_6H_4, *J* = 8.8 Hz), 10.03 (*s*, 1H, $C\underline{H}O$); **^{13}C NMR** (75.45 MHz, $CDCl_3$) δ (ppm) 119.0 (C^4), 120.6 (*q*, $\underline{C}F_3$, $^1J_{C-F}$ = 270.6 Hz), 123.9 (4-NO_2-C_6H_4), 125.6, 129.7, 129.9, (C_6H_5), 131.8, 133.2 (4-NO_2-C_6H_4), 137.4 (C_6H_5), 143.7 (*q*, C^3, $^2J_{C-F}$ = 39.4 Hz), 145.2 (C^5), 148.8 (4-NO_2-C_6H_4), 182.7 ($\underline{C}HO$); **HRMS** (ESI+): calcd for $C_{17}H_{11}F_3N_3O_3^+$, $[M+H]^+$: 362.0747, found 362.0755.

2.4. Antiproliferative activity

2.4.1. Cell culture

Promastigotes forms of *Leishmania amazonensis* (strain WHOM/ BR/75/JOSEFA) were cultured in Warren medium (brain heart infusion, hemin, and folic acid; pH 7.4) supplemented with 10% fetal bovine serum (FBS) at 25 °C. Epimastigote forms of *Trypanosoma cruzi* (Y strain) were cultured in LIT medium (liver infusion tryptose; hemim, and folic acid; pH 7.4) supplemented with 10% FBS at 28 ºC.

Cytotoxicity activity was determined in LLCMK2 (epithelial cells of kidney of Macaca mulatta) cells and J774A1 macrophages. $LLCMK_2$ (epithelial cells of kidney of *Macaca mulatta*) cells were cultured in Dulbecco's modified Eagle's medium (DMEM, pH 7.2) supplemented with 10% FBS at 37 °C in a 5% CO_2 atmosphere. J774A1 macrophages were cultured in RPMI-1640 (pH 7.2) medium supplemented with 10% FBS at 37 °C in a 5% CO_2 atmosphere.

2.4.2. Dilution of compounds

Stock solutions of the compounds were prepared in DMSO and then diluted in the respective medium. The groups (controls and treated) were tested with DMSO concentrations below 1%, with concentrations that do not affect viability of the protozoa and mammalian cells.

2.4.3. Antiproliferative assay

Promastigote forms (1×10^6 parasites/mL) were cultured in 96-well plates in the presence and absence of different concentrations of compounds diluted in Warren medium supplemented with 10% FBS and incubated for 72h. Epimastigote forms (1×10^6 parasites/mL) were cultured in 96-well plates in the presence and absence of different concentrations of compounds diluted in LIT medium supplemented with 10% FBS and incubated for 96h. After treatment, the parasites were incubated with a solution of 2,3-bis (2-methoxy-4-nitro-5-sulfophenyl)-2H-tetrazolium-5-carboxanilide (XTT; 0.5 mg/mL) and phenazine methanesulfonate activator (PMS; 0.06 mg/mL) in PBS for 4h. Then, the absorbance was read in a microplate reader (Bio Tek - Power Wave XS) at 450 nm. The percentage of viable parasites was calculated in relation to the control in order to determine the concentration that inhibits 50% of the protozoa (IC_{50}).

2.4.4. Cytotoxicity assay

$LLCMK_2$ cells (2.5×10^5 cells/mL) suspension was prepared in DMEM medium supplemented with 10% FBS and added to 96-well plates. Then, the plates were incubated at 37 °C in a CO_2 atmosphere for 24h to obtain confluent cell growth. After incubation, cells were treated or not with different concentrations of compounds diluted in DMEM for 72 h. Macrophage (5×10^5 cells/mL) suspension was prepared in RPMI-1640 medium supplemented with 10% FBS and added to 96-well plates. Then, the plates were incubated at 37 °C in a CO_2 atmosphere for 24h to obtain confluent cell growth. After incubation, cells were treated or not with different concentrations of compounds diluted in RPMI-1640 for 48h. After treatment, medium was removed and cells were incubated with MTT (2 mg/mL) for 4h. Then, DMSO was added for solubilization of the formazan and analyzed with a reading

microplate reader (BIO-TEK PowerWave XS spectrophotometer) at 392 nm. The percentage of viable cells was calculated in relation to the control to determine the cytotoxic concentration to 50% of the cells (CC_{50}).

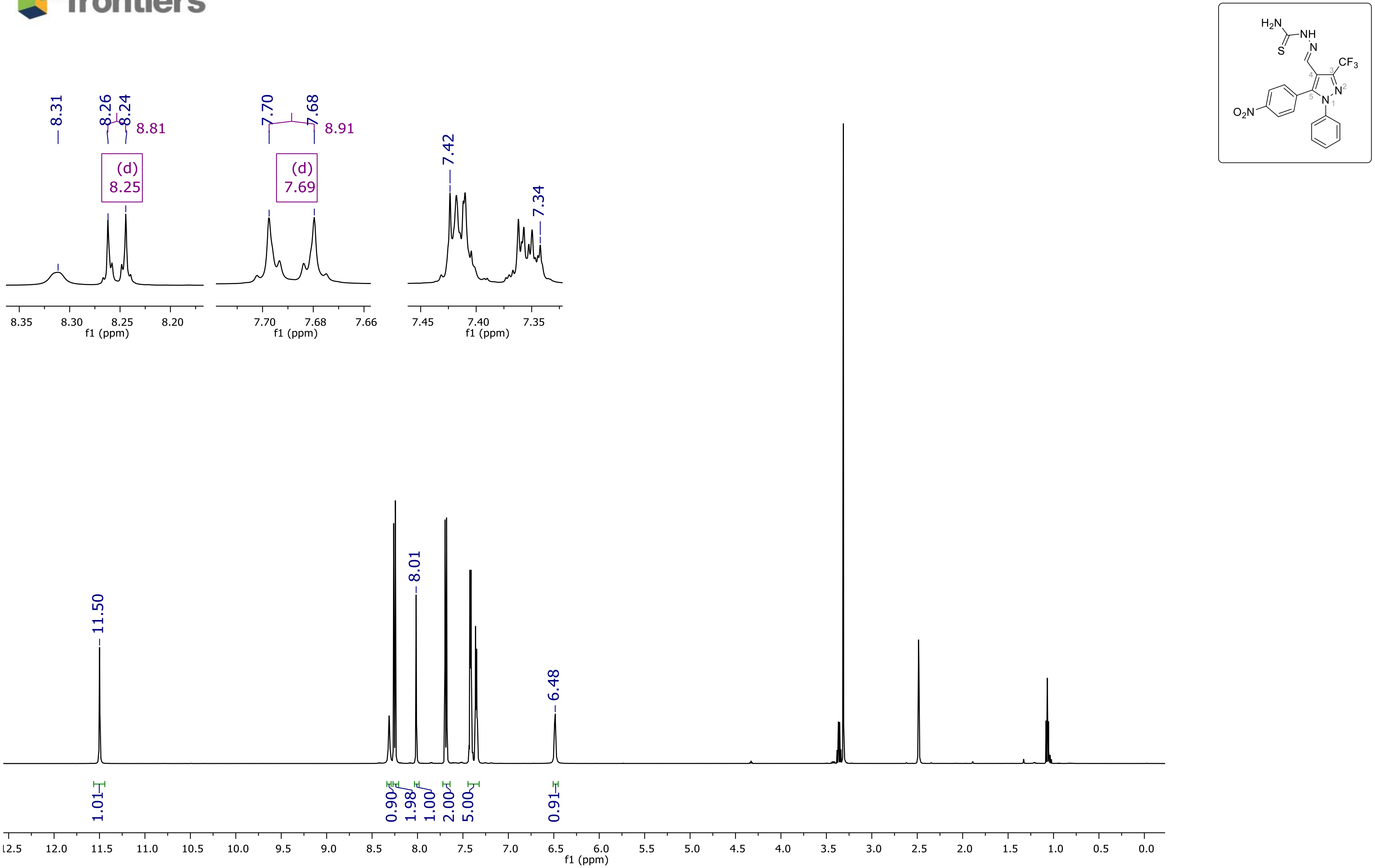

Figure S1 – ^{1}H NMR spectrum of compound **2a** in DMSO-d_6 at 500.13 MHz.

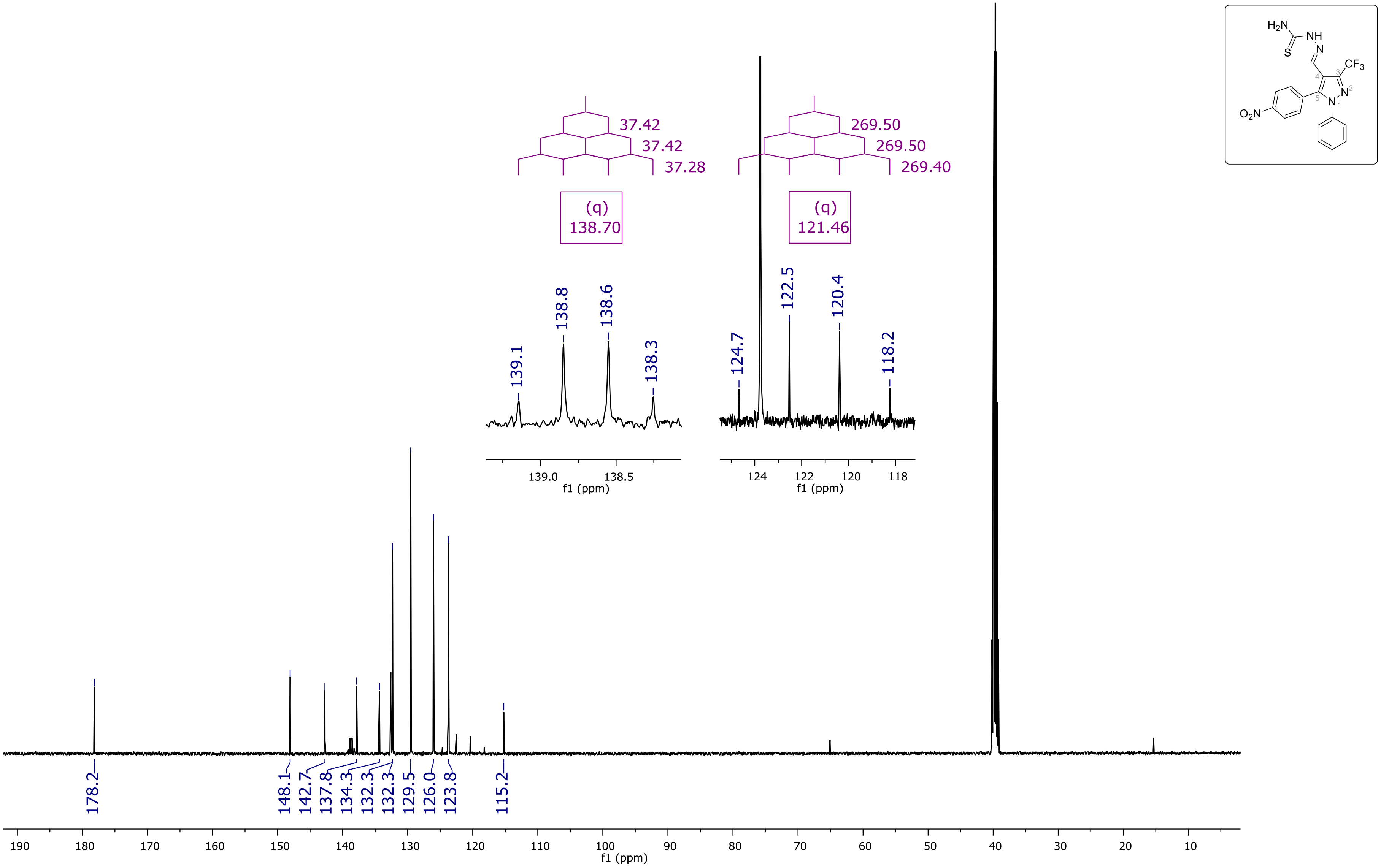

Figure S2 – ^{13}C NMR spectrum of compound **2a** in DMSO-d_6 at 125.76 MHz.

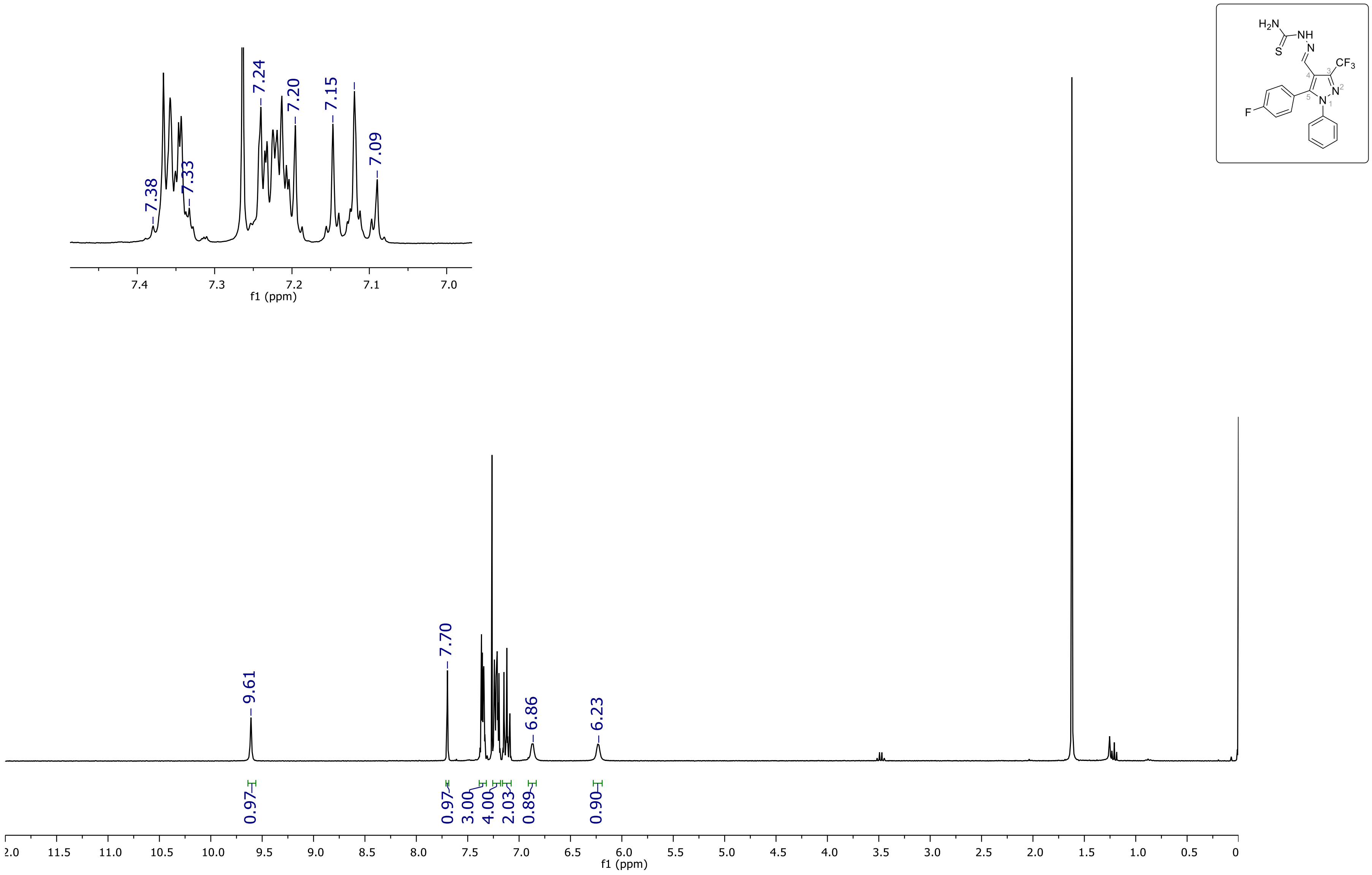

Figure S3 – ^{1}H NMR spectrum of compound **2b** in $CDCl_3$ at 300.06 MHz.

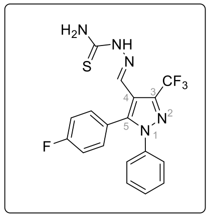

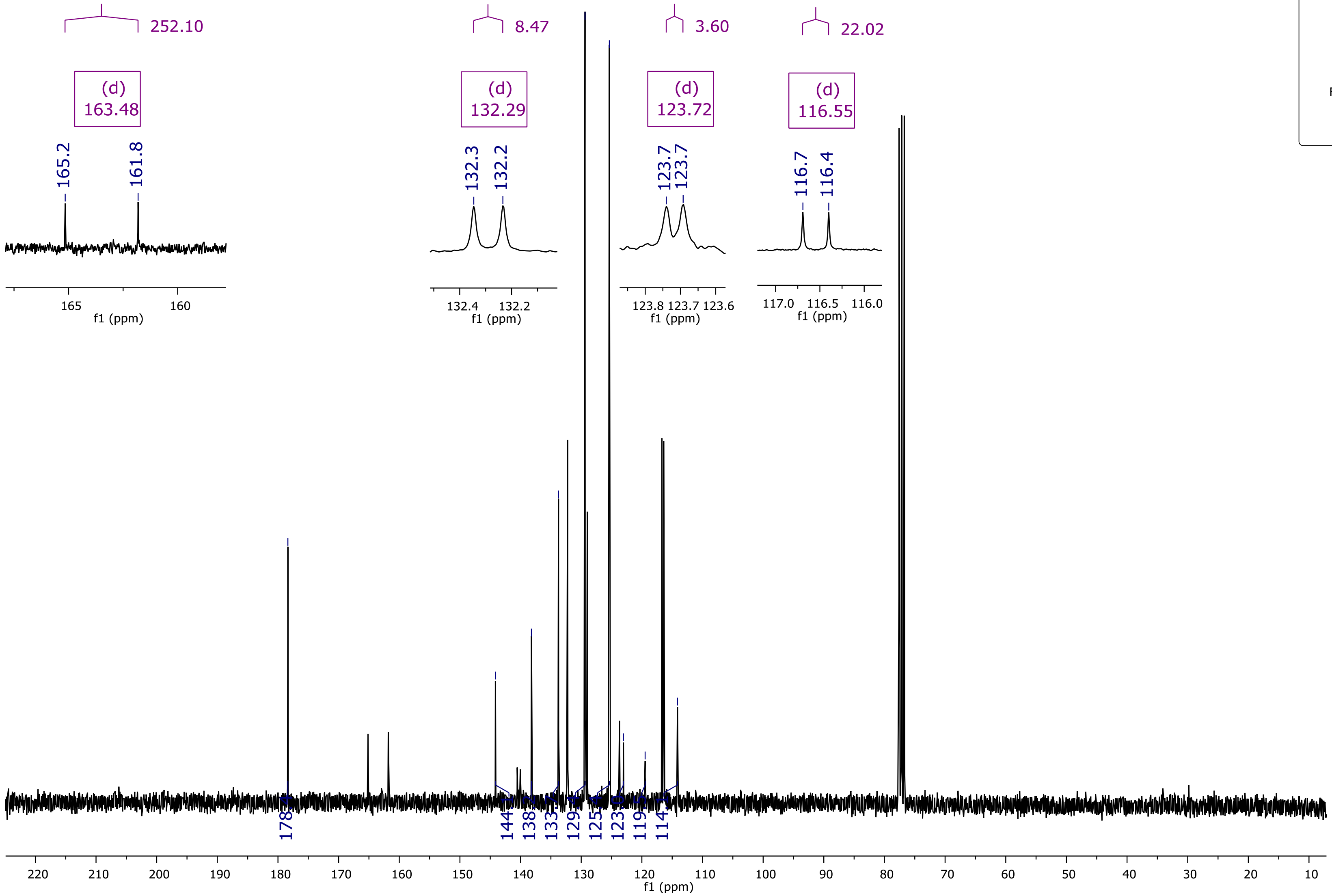

Figure S4 – ^{13}C NMR spectrum of compound **2b** in $CDCl_3$ at 75.46 MHz.

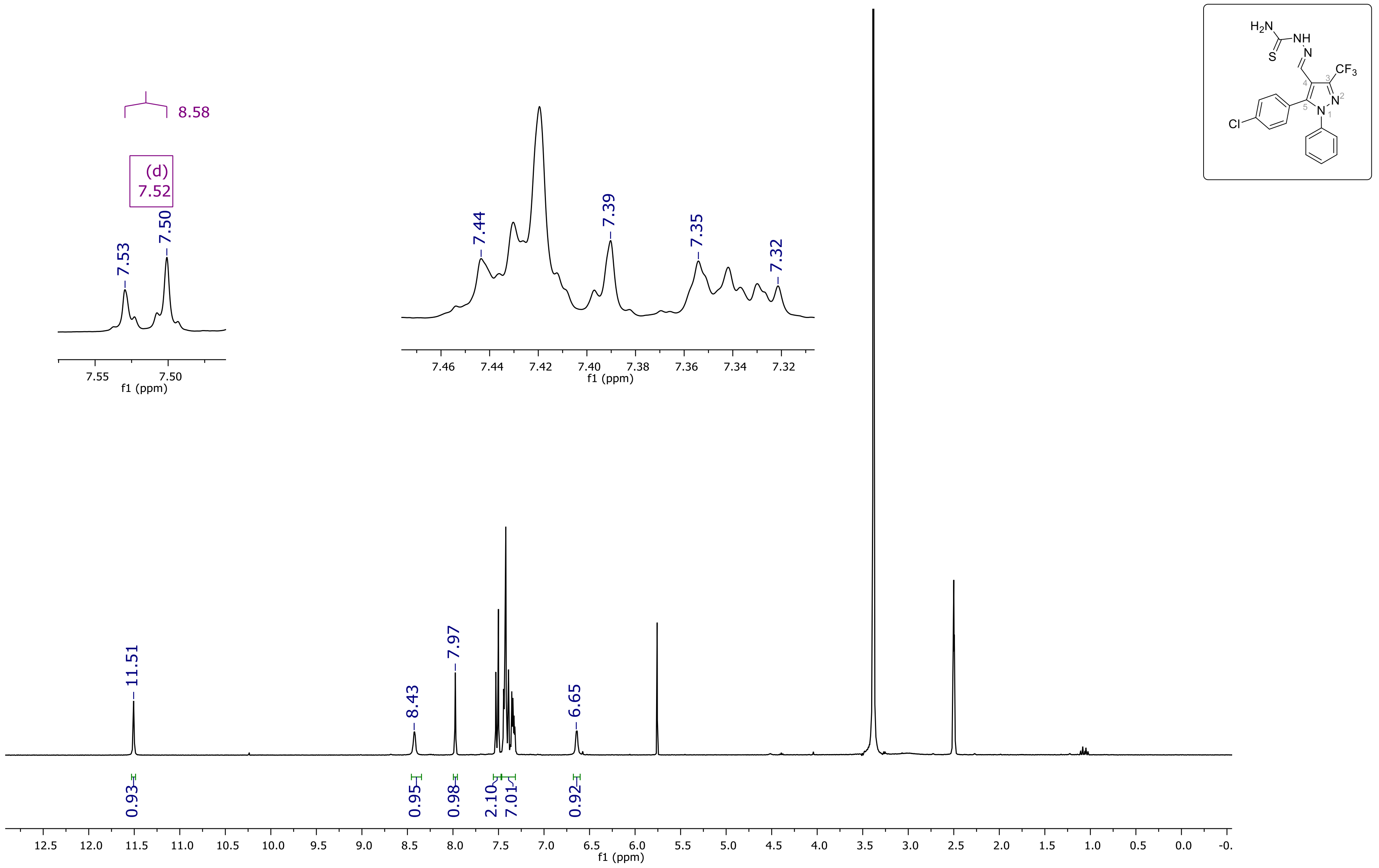

Figure S5 – ^{1}H NMR spectrum of compound **2c** in DMSO-d_6 at 300.06 MHz.

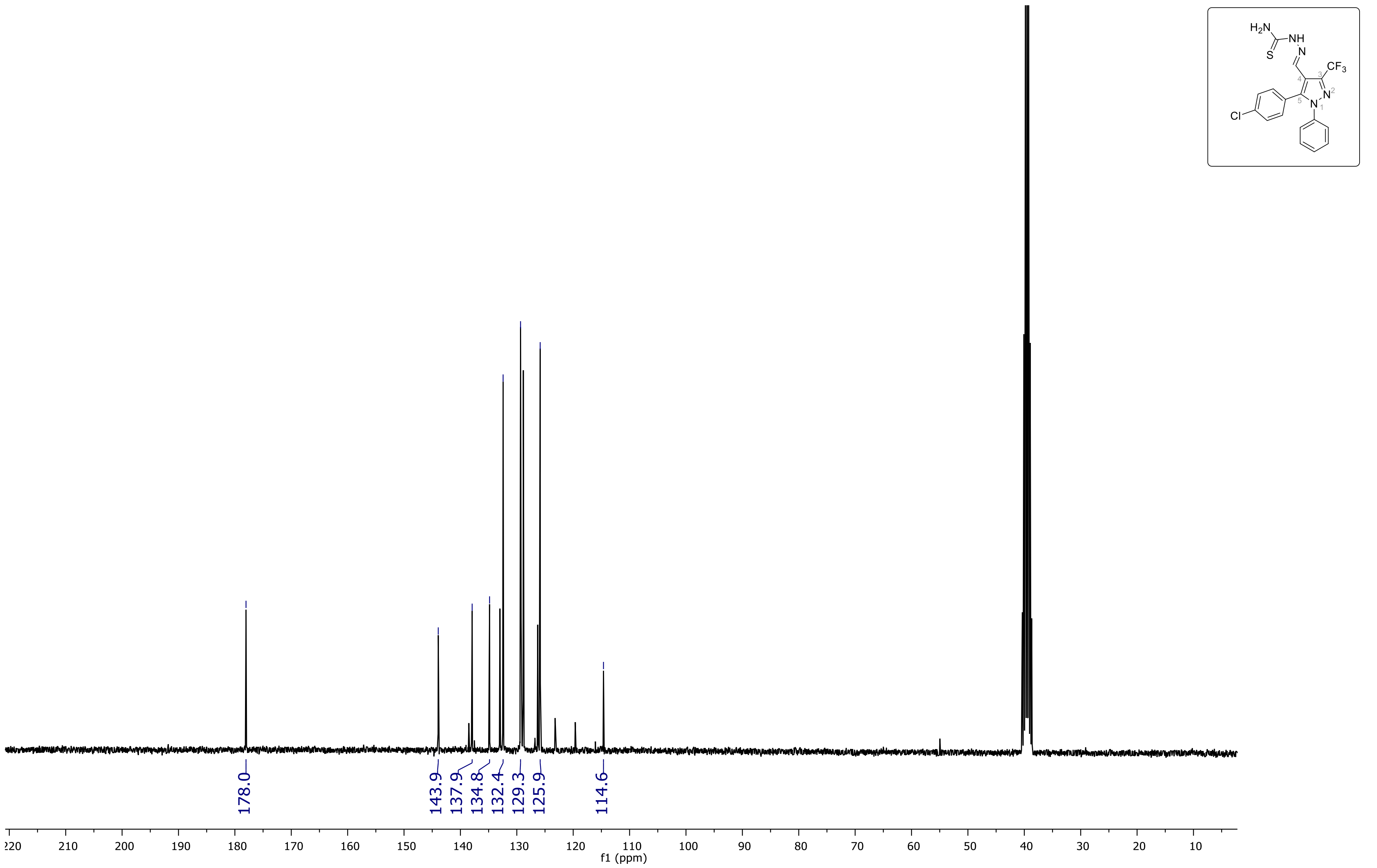

Figure S6 – ^{13}C NMR spectrum of compound **2c** in DMSO-d_6 at 75.46 MHz.

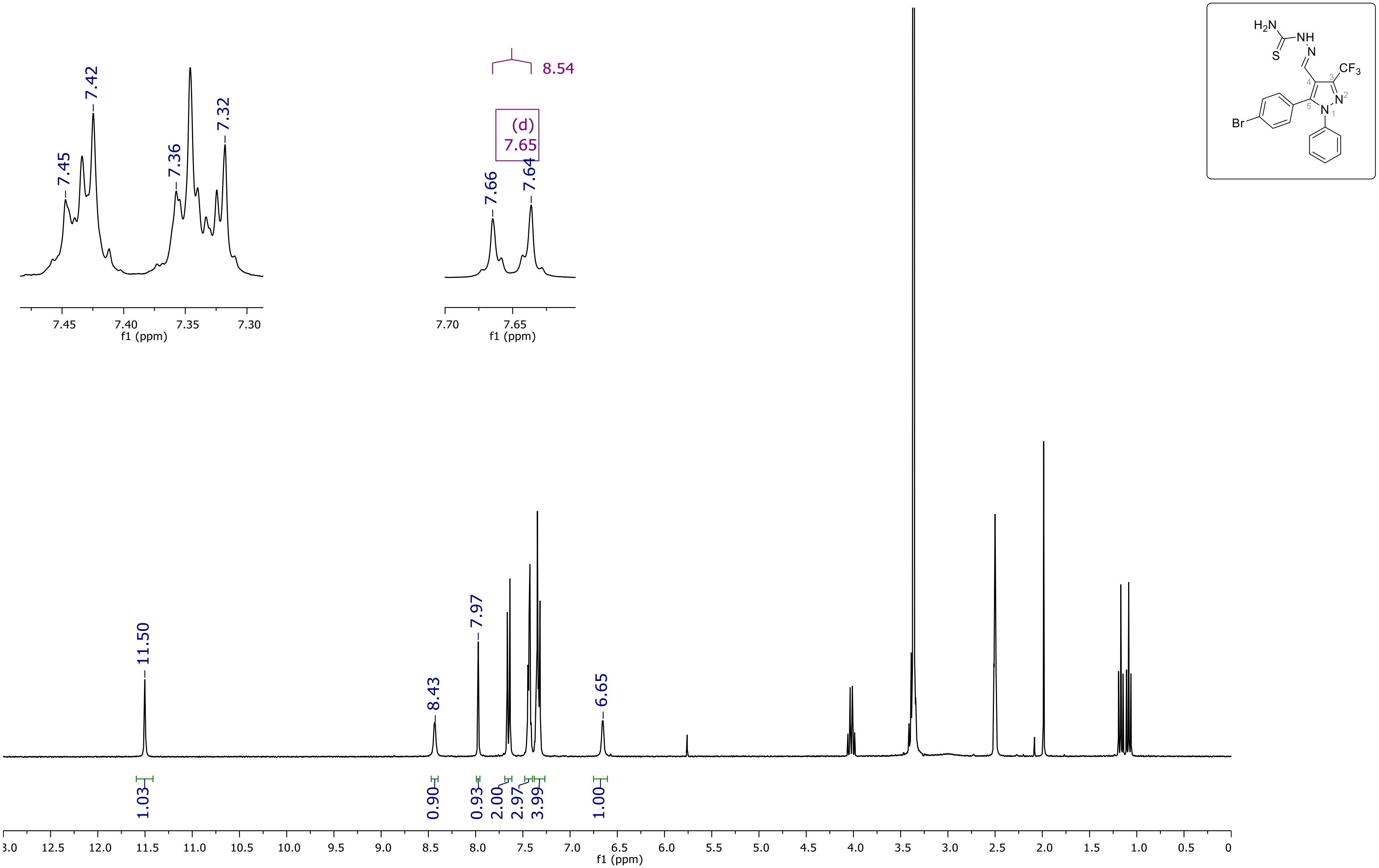

Figure S7 – ^{1}H NMR spectrum of compound **2d** in DMSO-d_6 at 300.06 MHz.

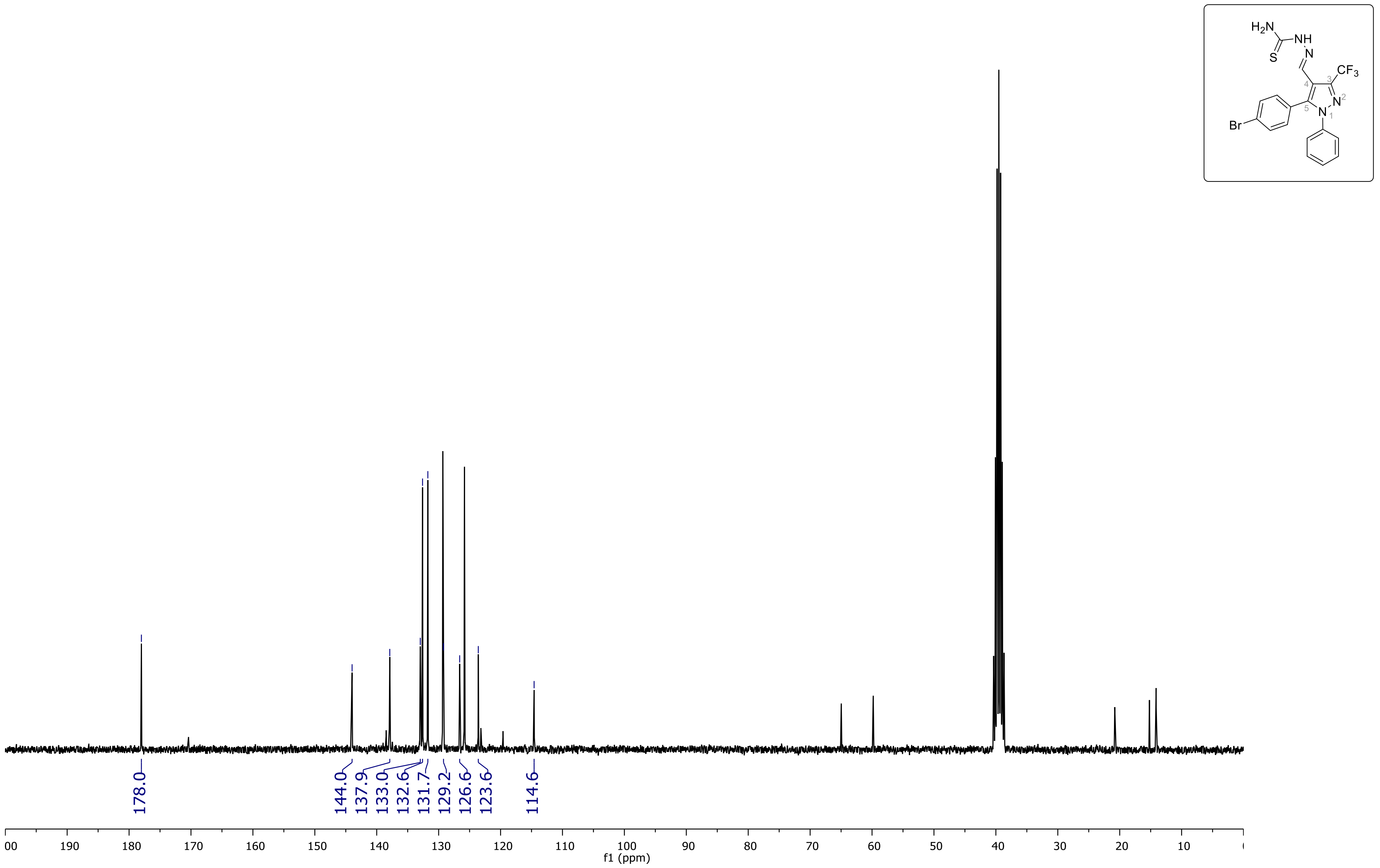

Figure S8 – ^{13}C NMR spectrum of compound **2d** in DMSO-d_6 at 75.46 MHz.

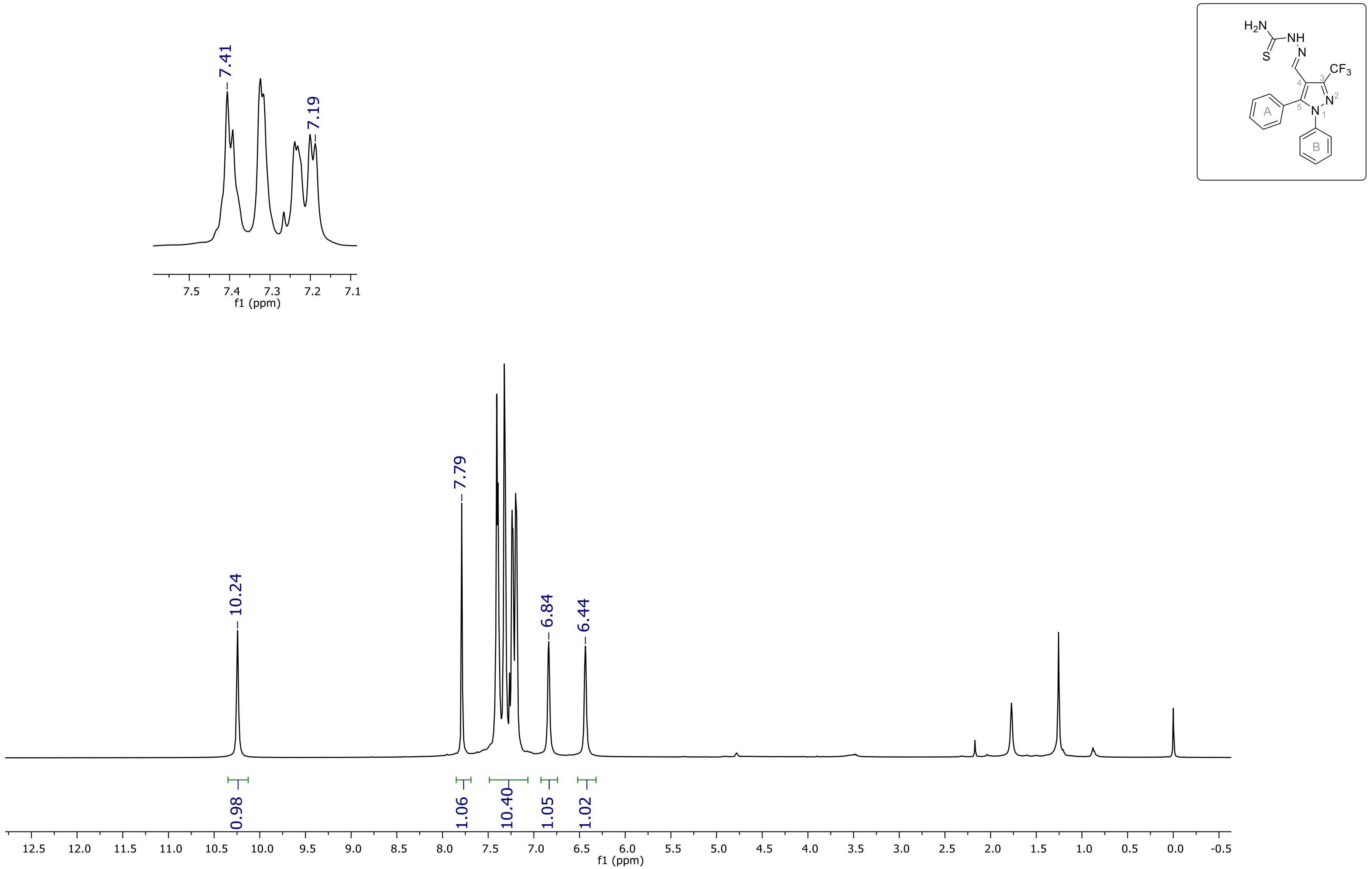

Figure S9 – ^{1}H NMR spectrum of compound **2e** in $CDCl_3$ at 300.06 MHz.

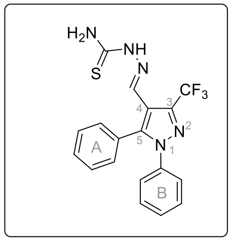

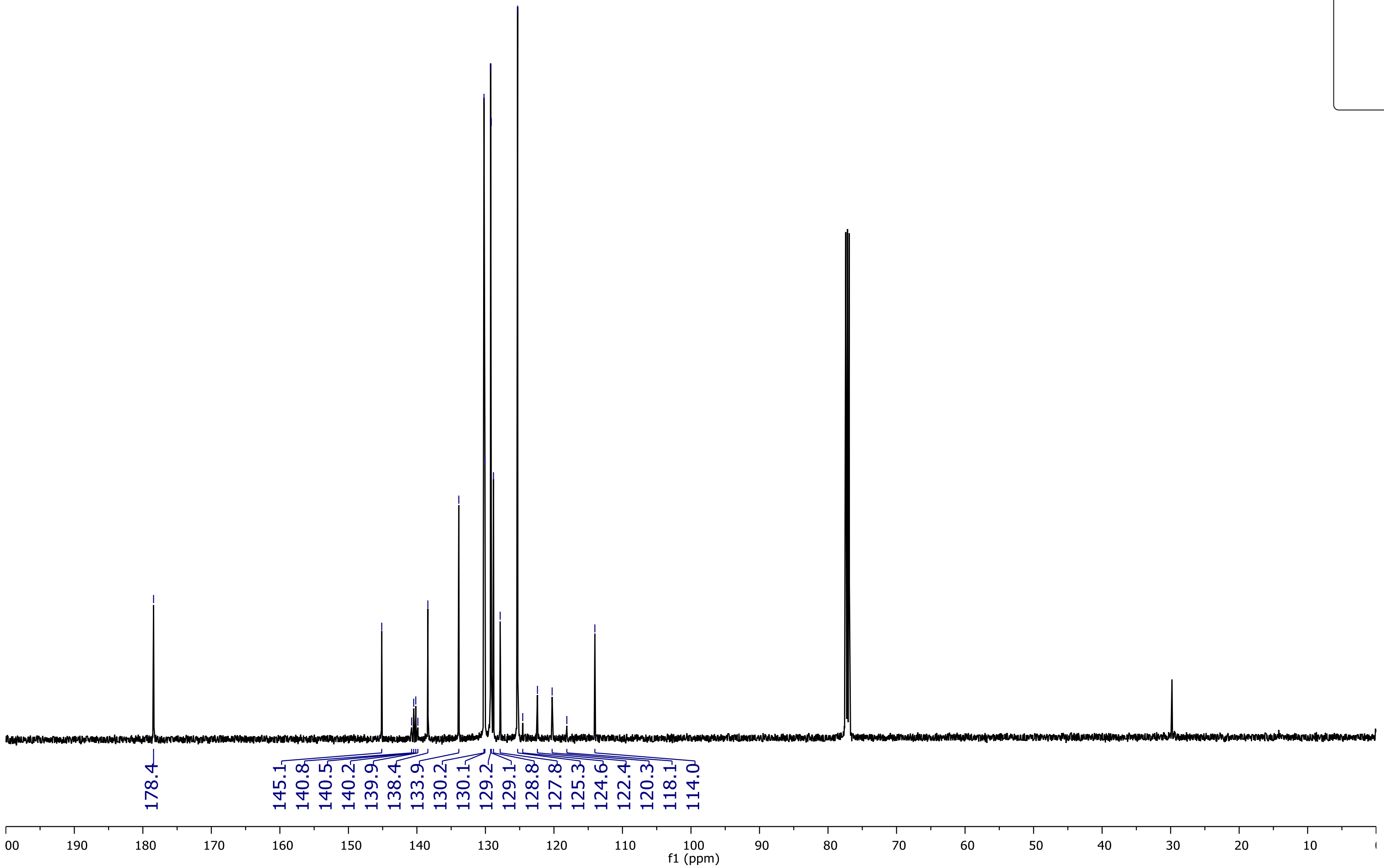

Figure S10 – ^{13}C NMR spectrum of compound **2e** in $CDCl_3$ at 75.46 MHz.

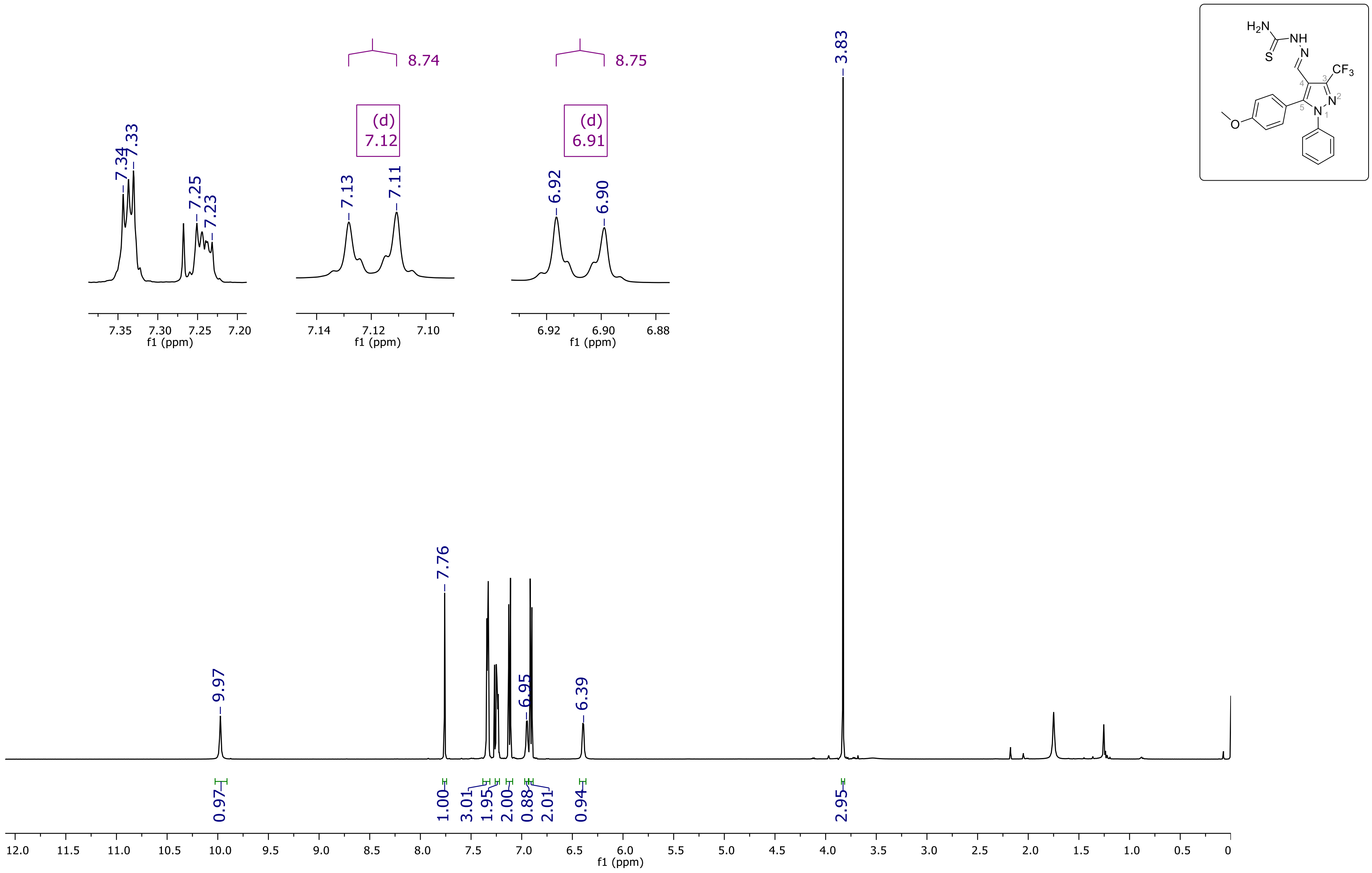

Figure S11 – ^{1}H NMR spectrum of compound **2f** in $CDCl_3$ at 300.06 MHz.

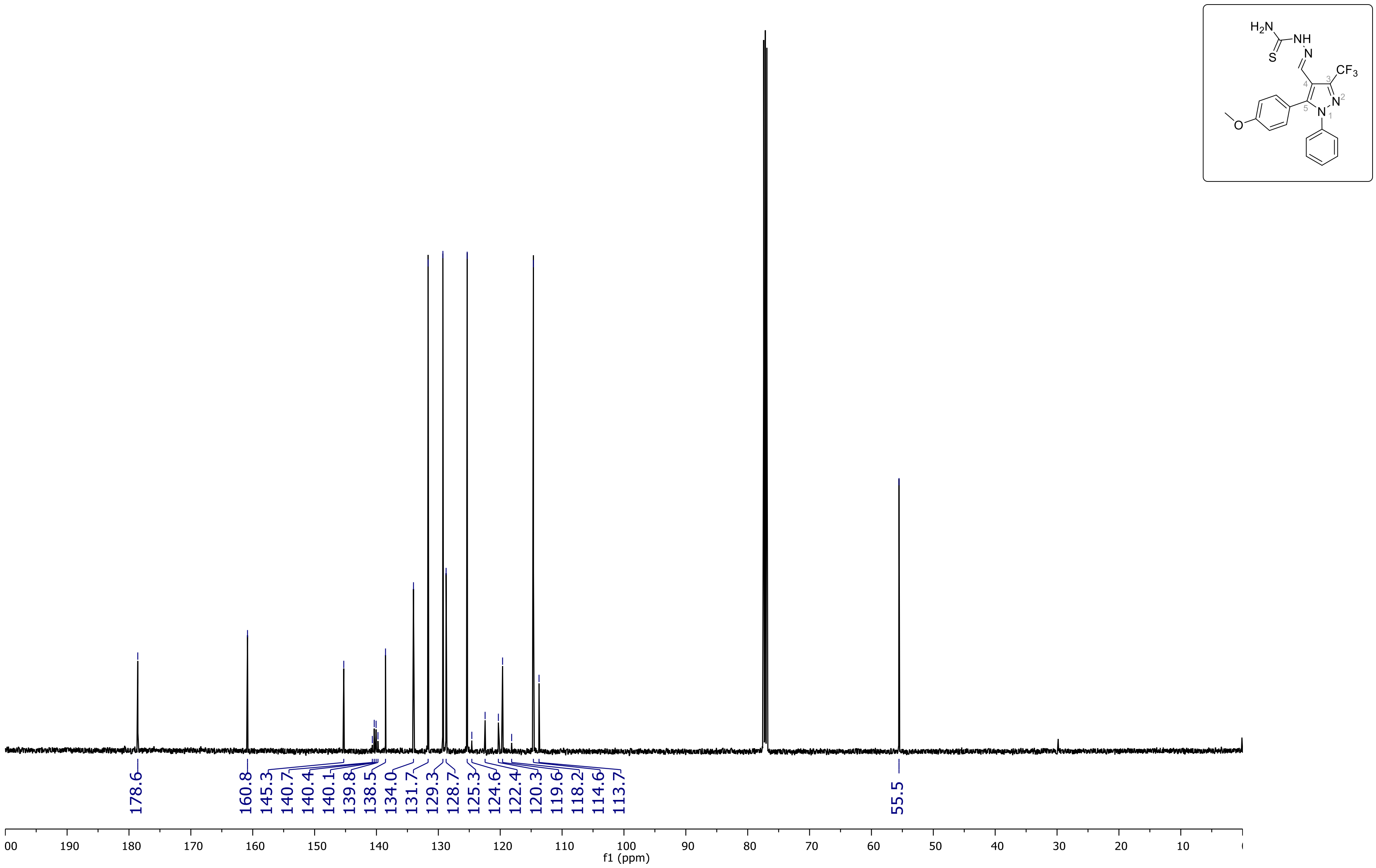

Figure S12 – ^{13}C NMR spectrum of compound **2f** in $CDCl_3$ at 75.46 MHz.

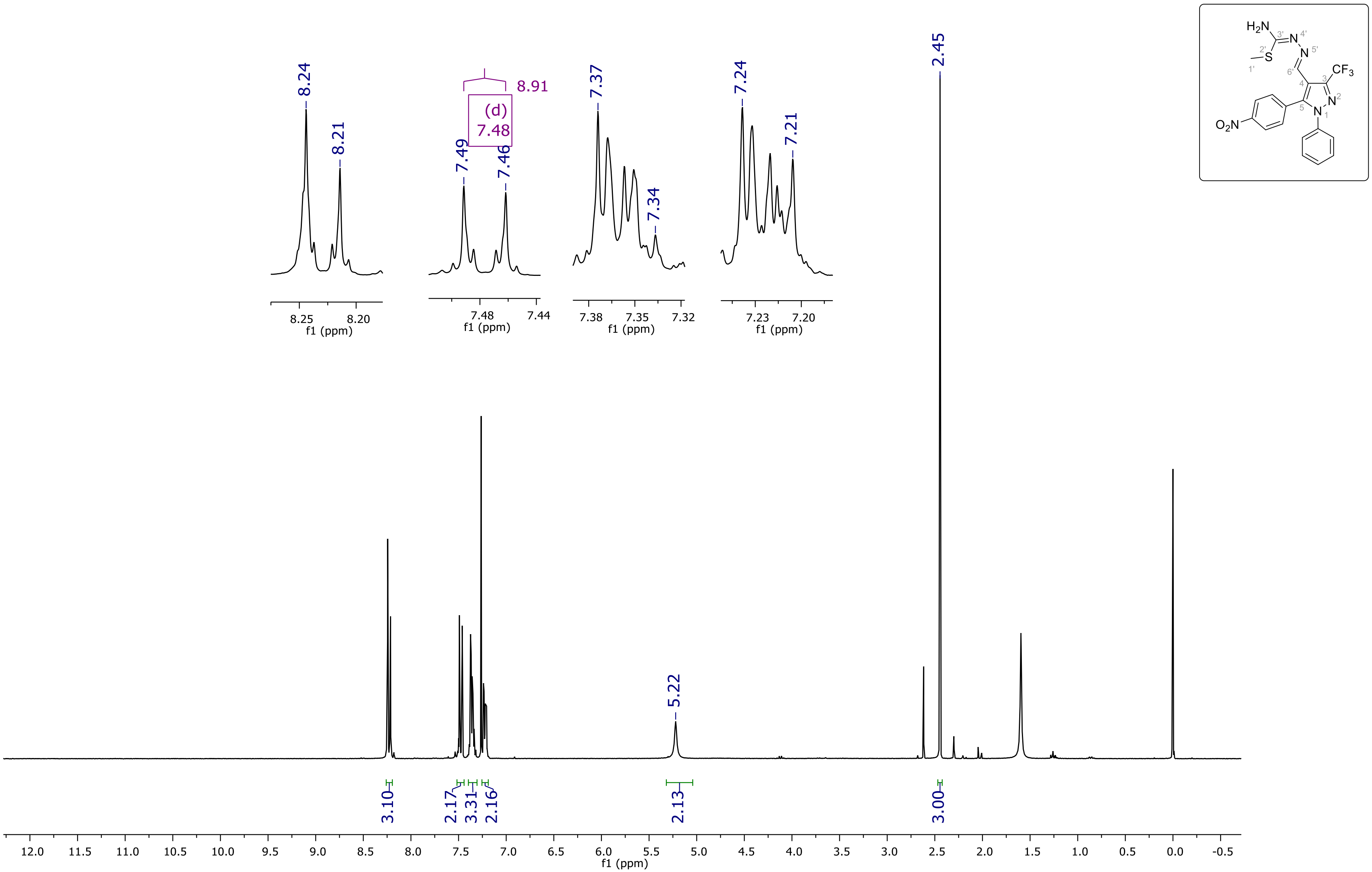

Figure S13 – ^{1}H NMR spectrum of compound **3a** in $CDCl_3$ at 300.06 MHz.

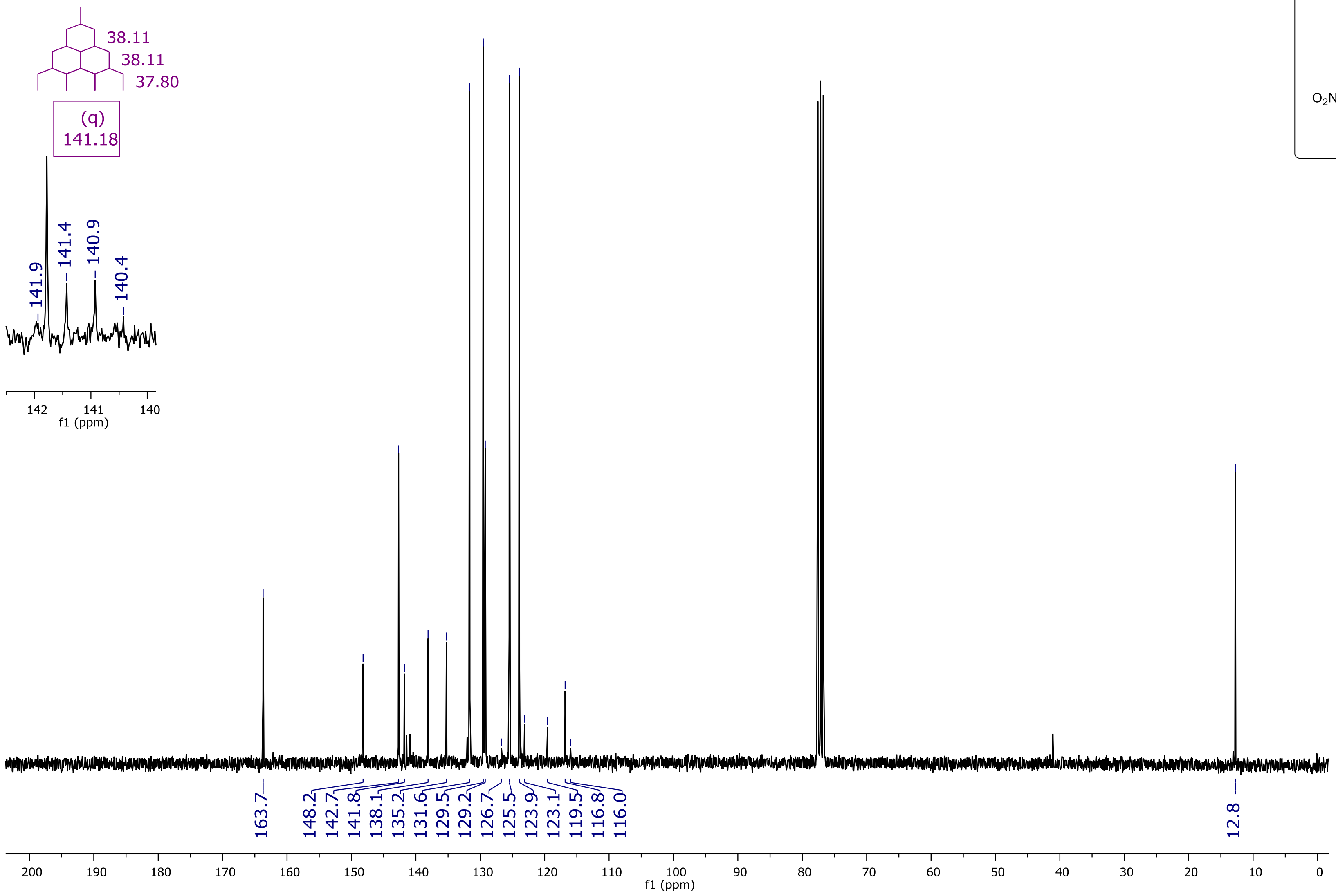

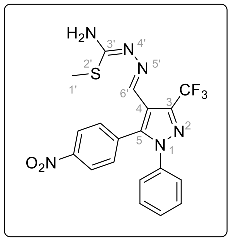

Figure S14 – ^{13}C NMR spectrum of compound **3a** in $CDCl_3$ at 75.46 MHz.

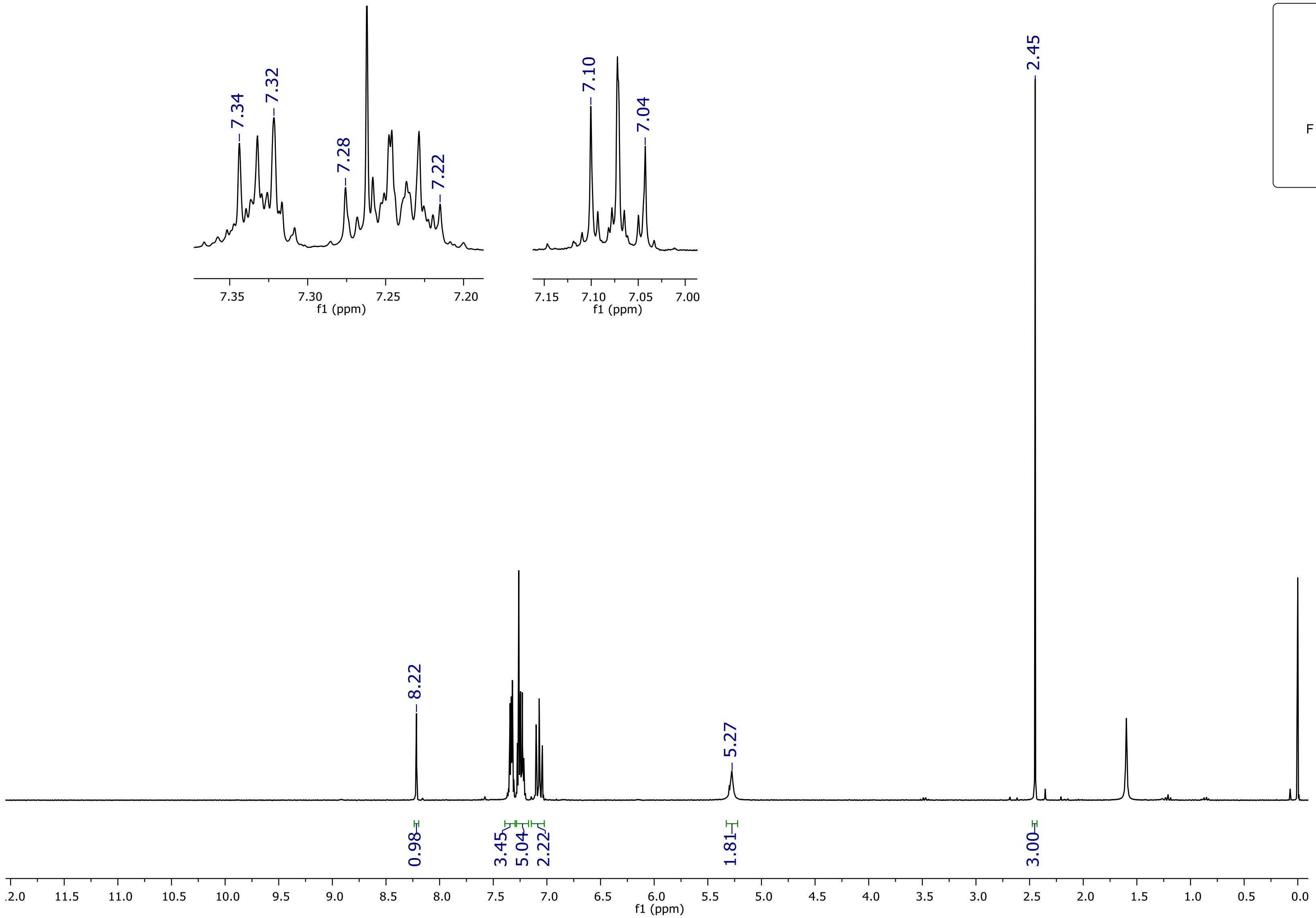

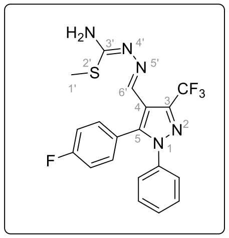

Figure S15 – ^{1}H NMR spectrum of compound **3b** in $CDCl_3$ at 300.06 MHz.

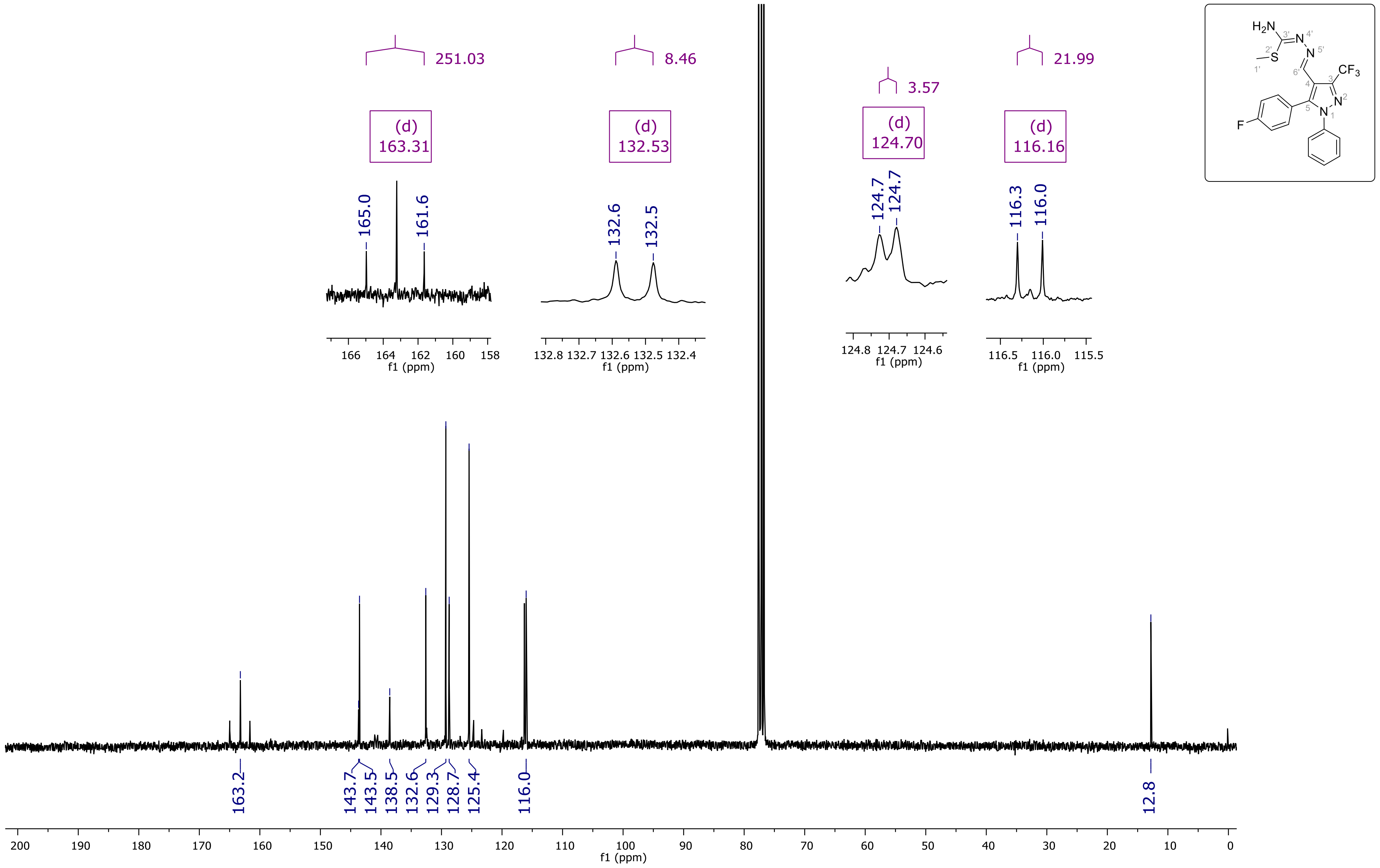

Figure S16 – ^{13}C NMR spectrum of compound **3b** in $CDCl_3$ at 75.46 MHz.

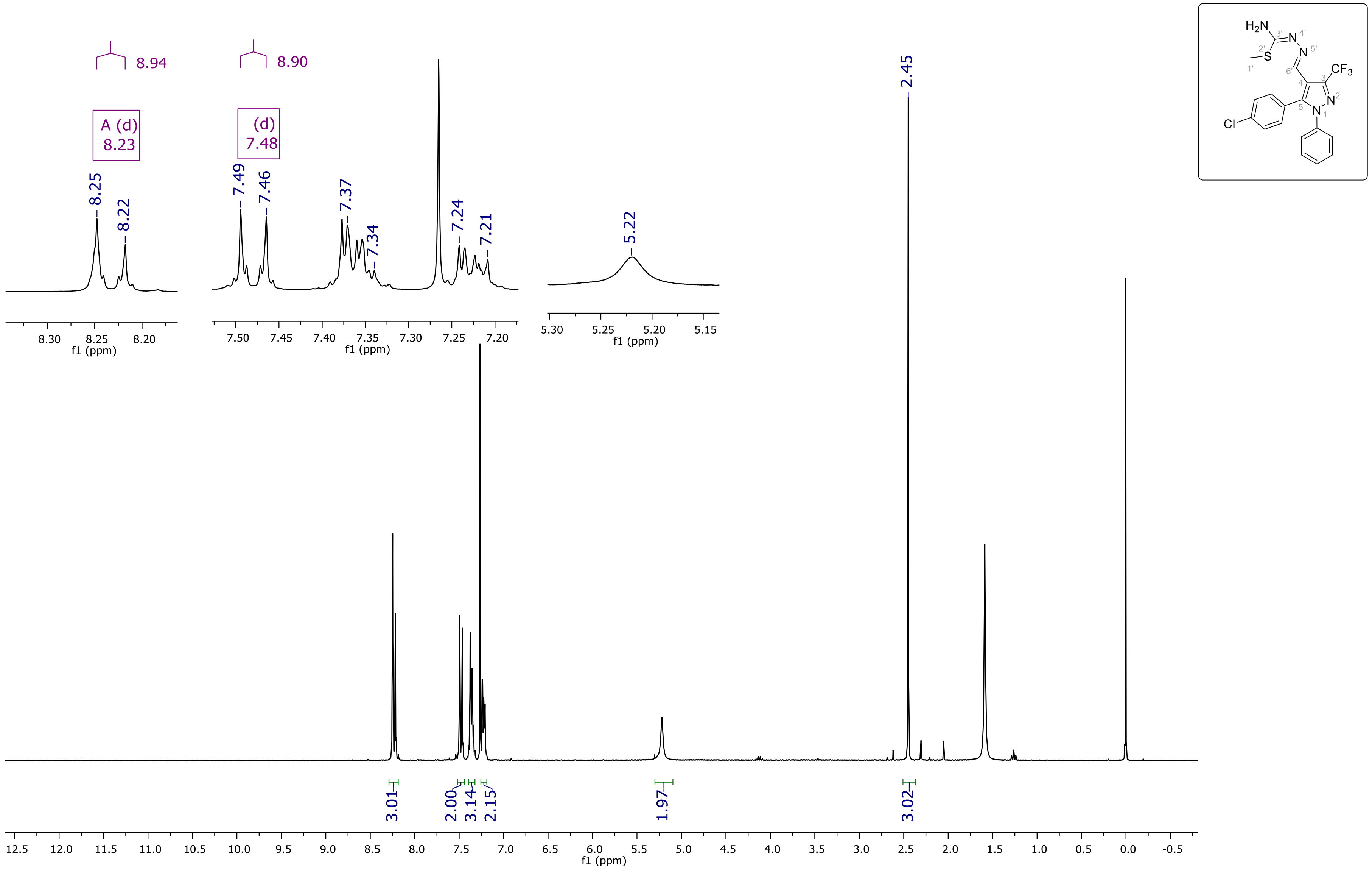

Figure S17 – ^{1}H NMR spectrum of compound **3c** in $CDCl_3$ at 300.06 MHz.

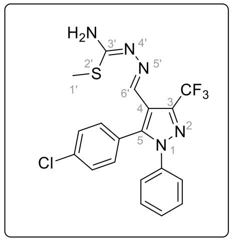

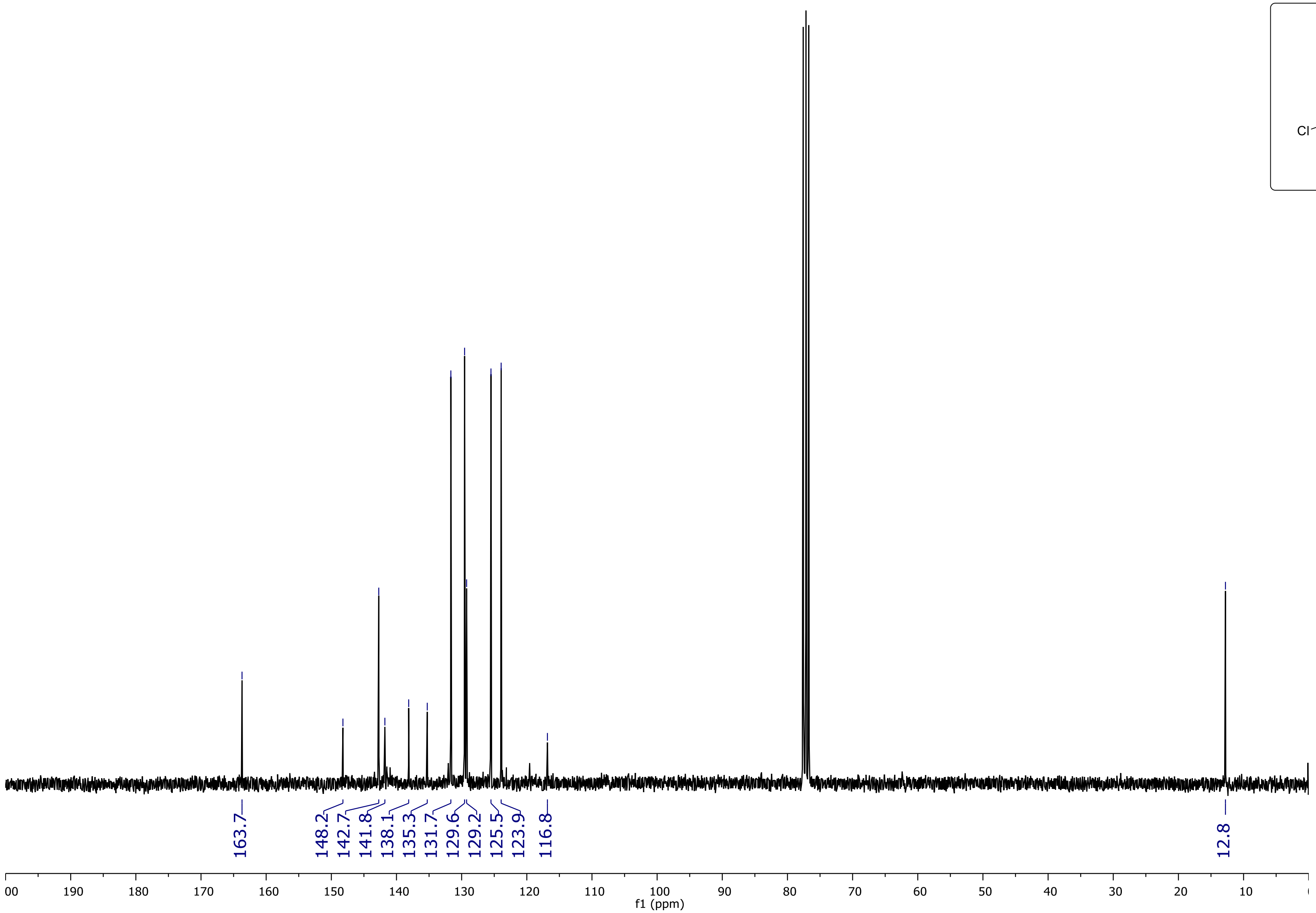

Figure S18 – ^{13}C NMR spectrum of compound **3c** in $CDCl_3$ at 75.46 MHz.

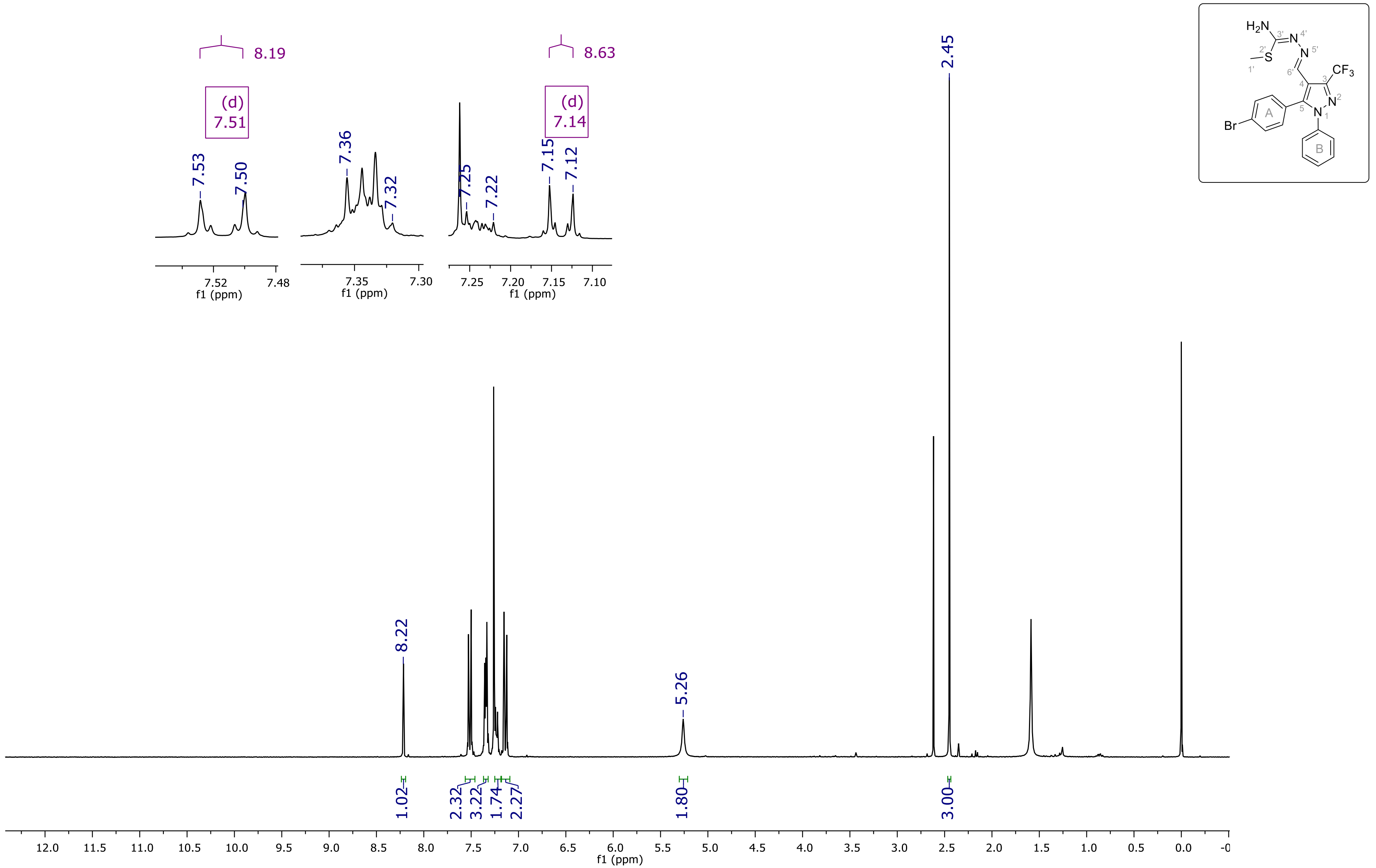

Figure S19 – ^{1}H NMR spectrum of compound **3d** in $CDCl_3$ at 300.06 MHz.

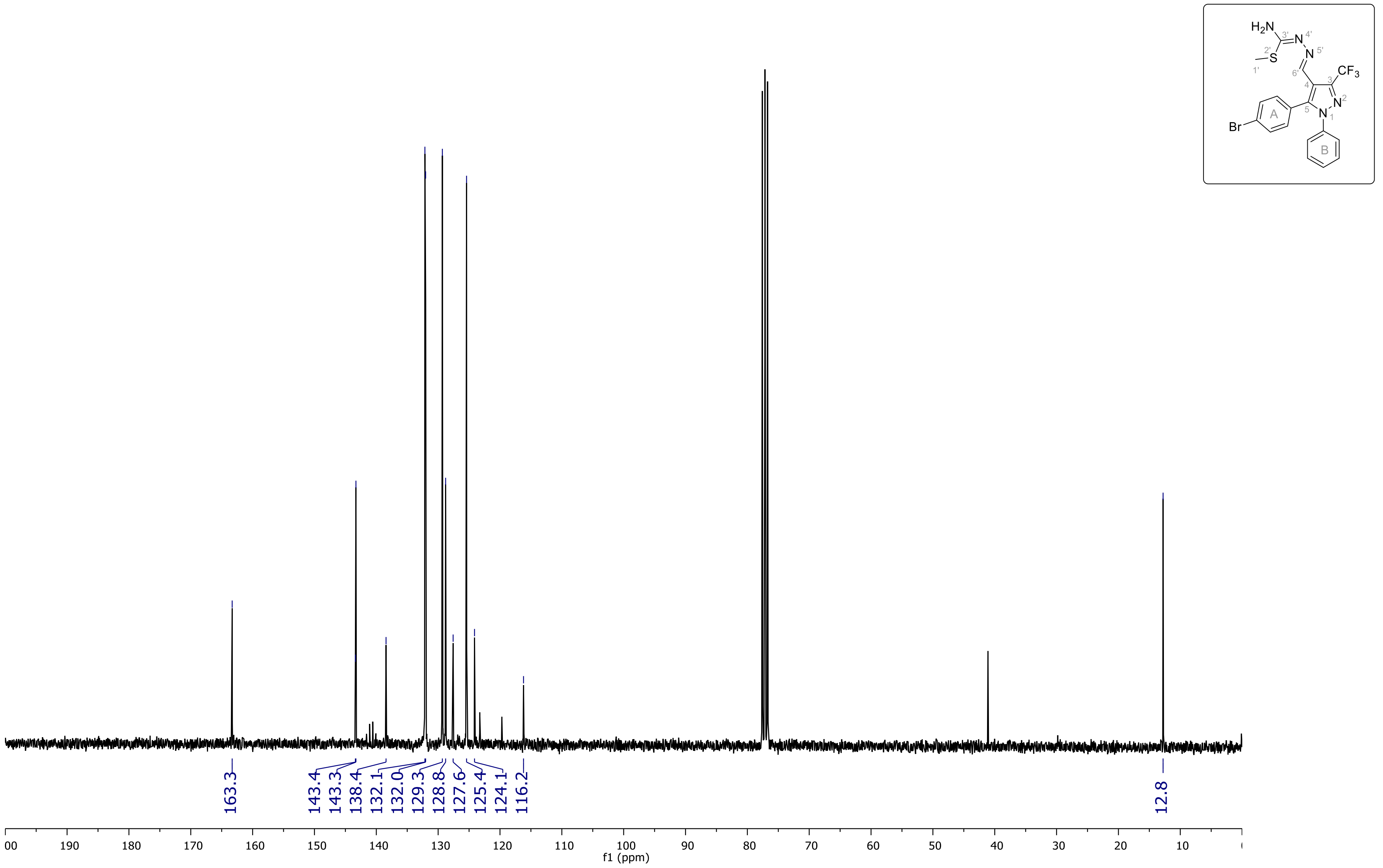

Figure S20 – ^{13}C NMR spectrum of compound **3d** in $CDCl_3$ at 75.46 MHz.

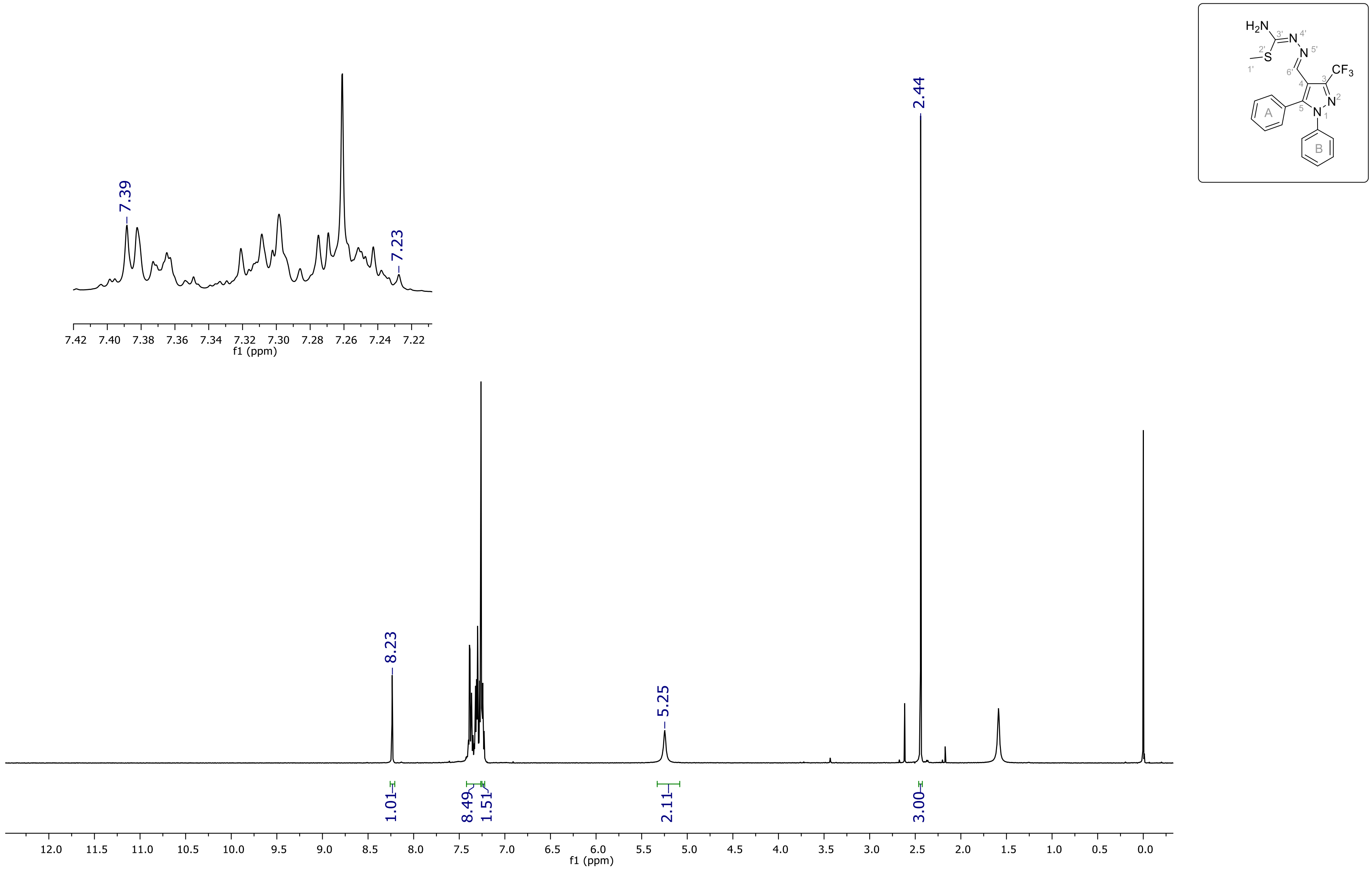

Figure S21 – ^{1}H NMR spectrum of compound **3e** in $CDCl_3$ at 300.06 MHz.

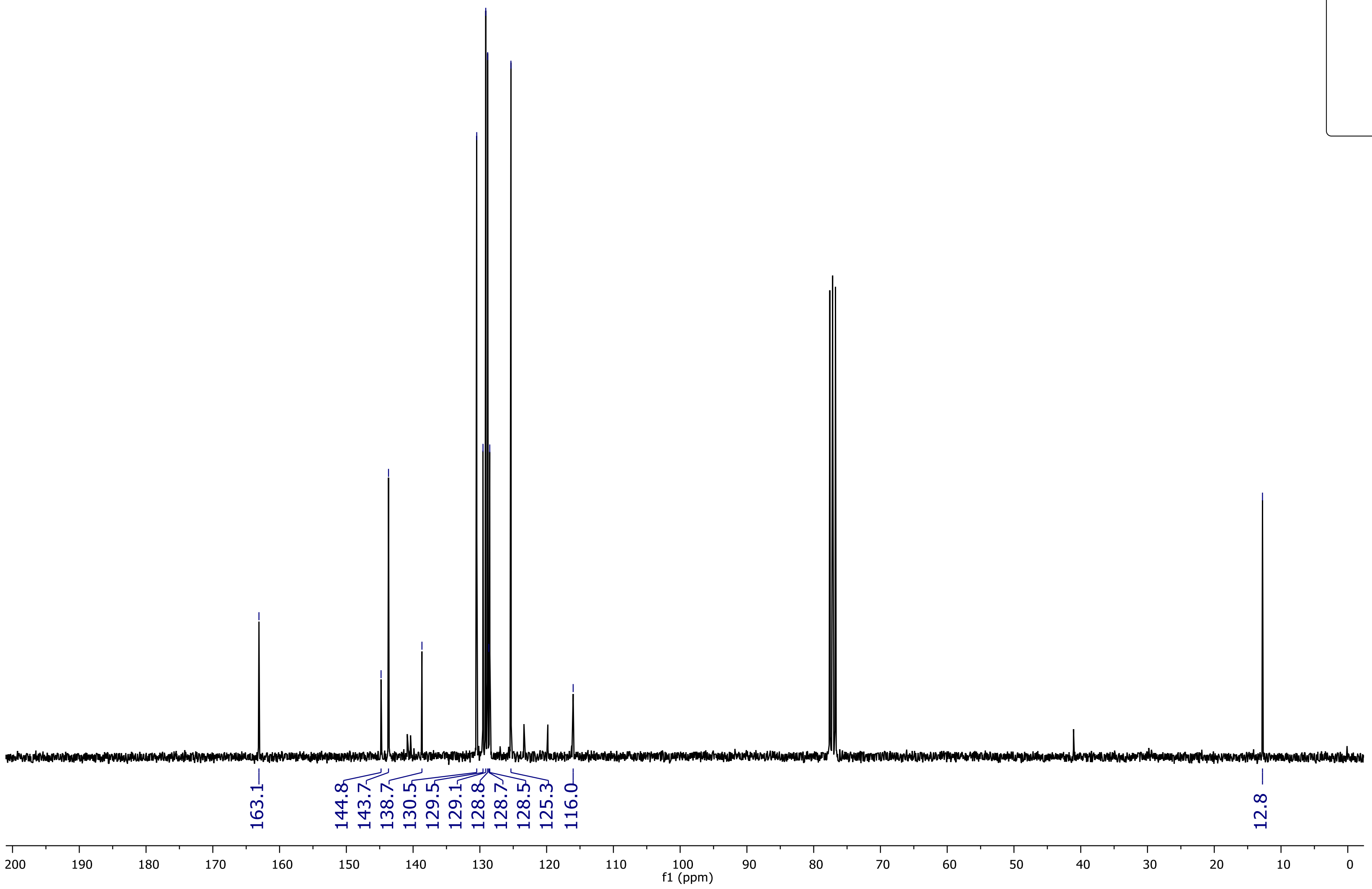

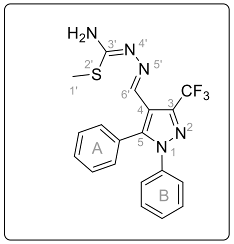

Figure S22 – ^{13}C NMR spectrum of compound **3e** in $CDCl_3$ at 75.46 MHz.

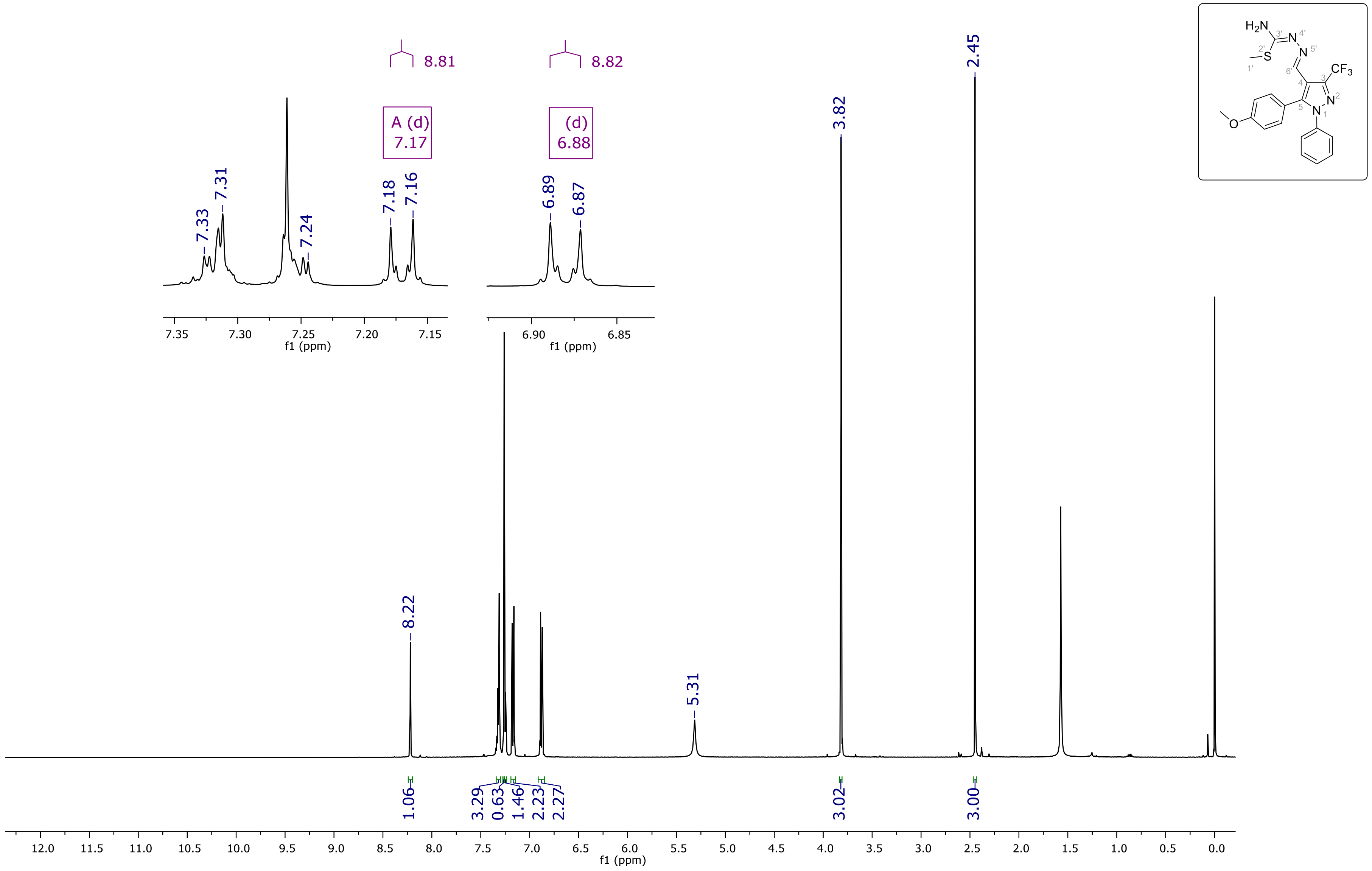

Figure S23 – ^{1}H NMR spectrum of compound **3f** in $CDCl_3$ at 300.06 MHz.

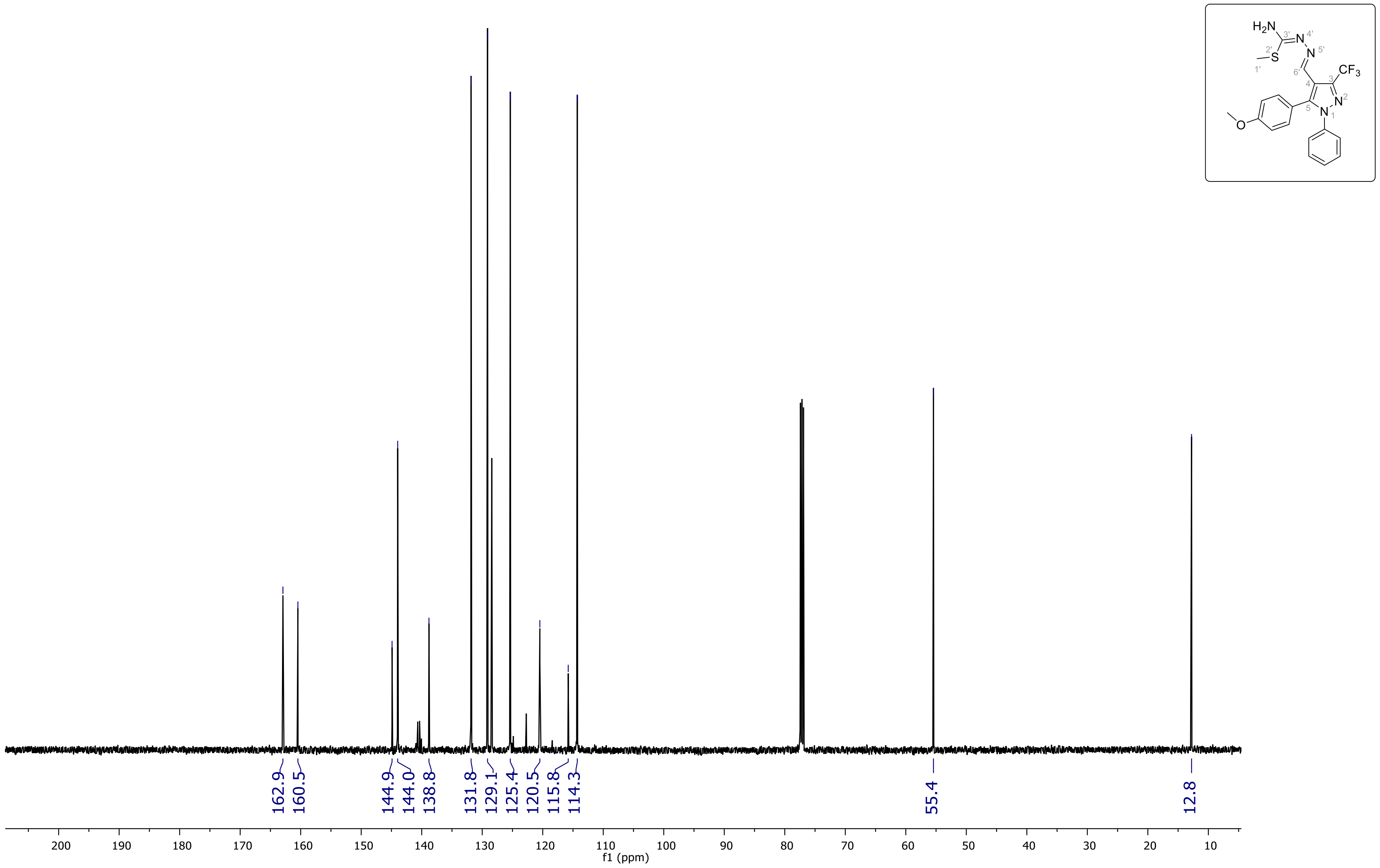

Figure S24 – ^{13}C NMR spectrum of compound **3f** in $CDCl_3$ at 75.46 MHz.

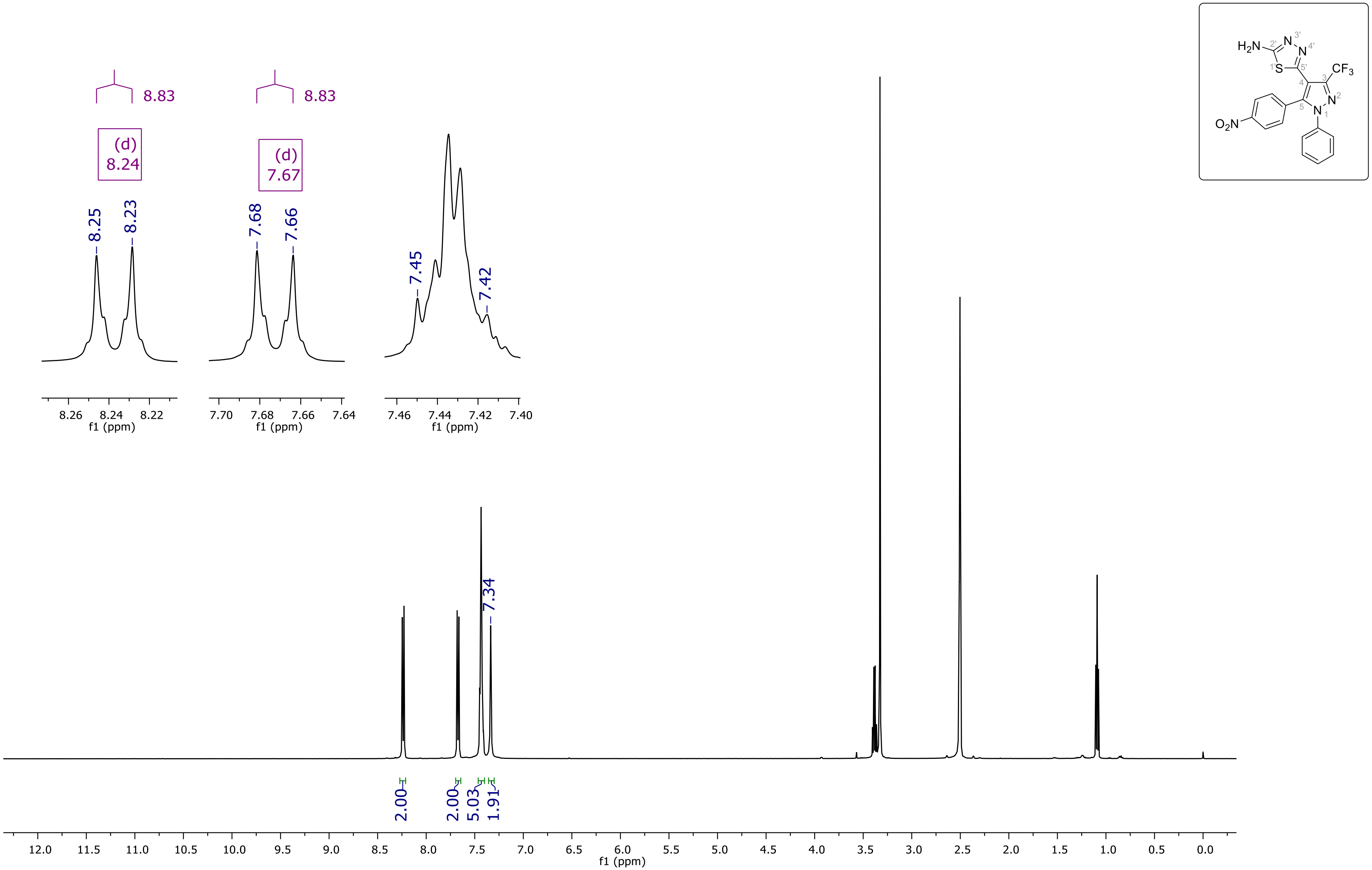

Figure S25 – ^{1}H NMR spectrum of compound **4a** in DMSO-d_6 at 300.06 MHz.

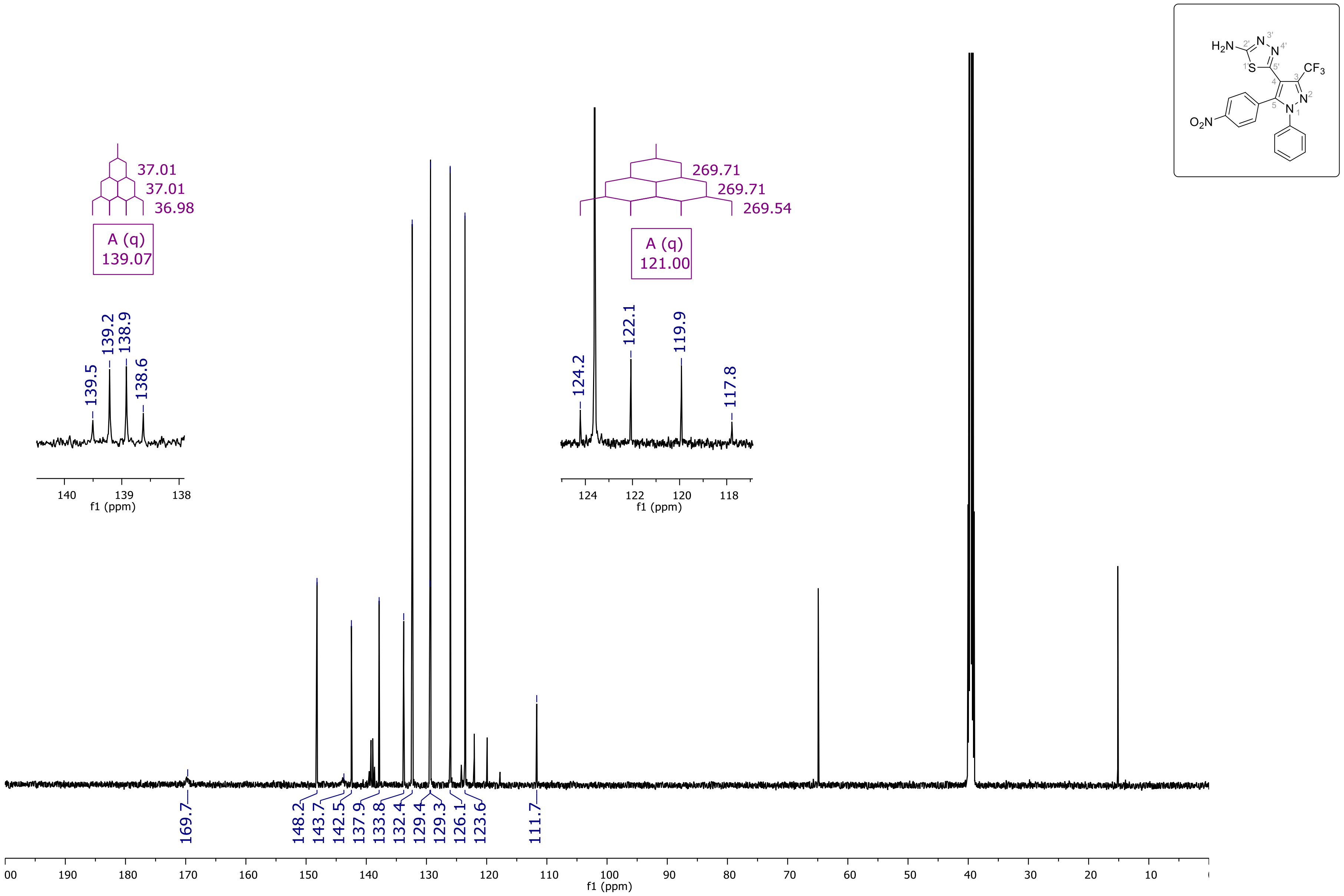

Figure S26 – ^{13}C NMR spectrum of compound **4a** in DMSO-d_6 at 75.46 MHz.

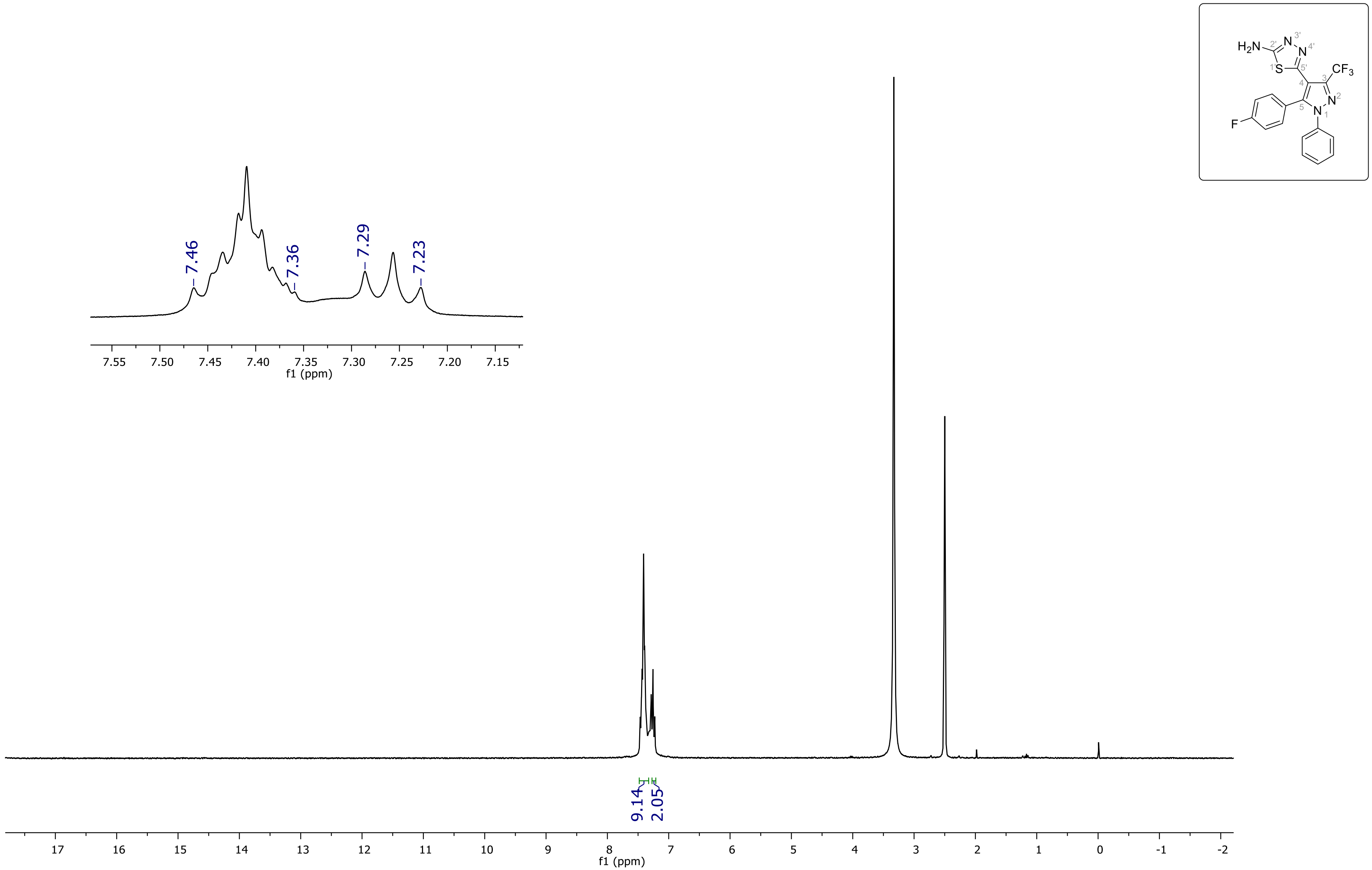

Figure S27 – ^{1}H NMR spectrum of compound **4b** in DMSO-d_6 at 300.06 MHz.

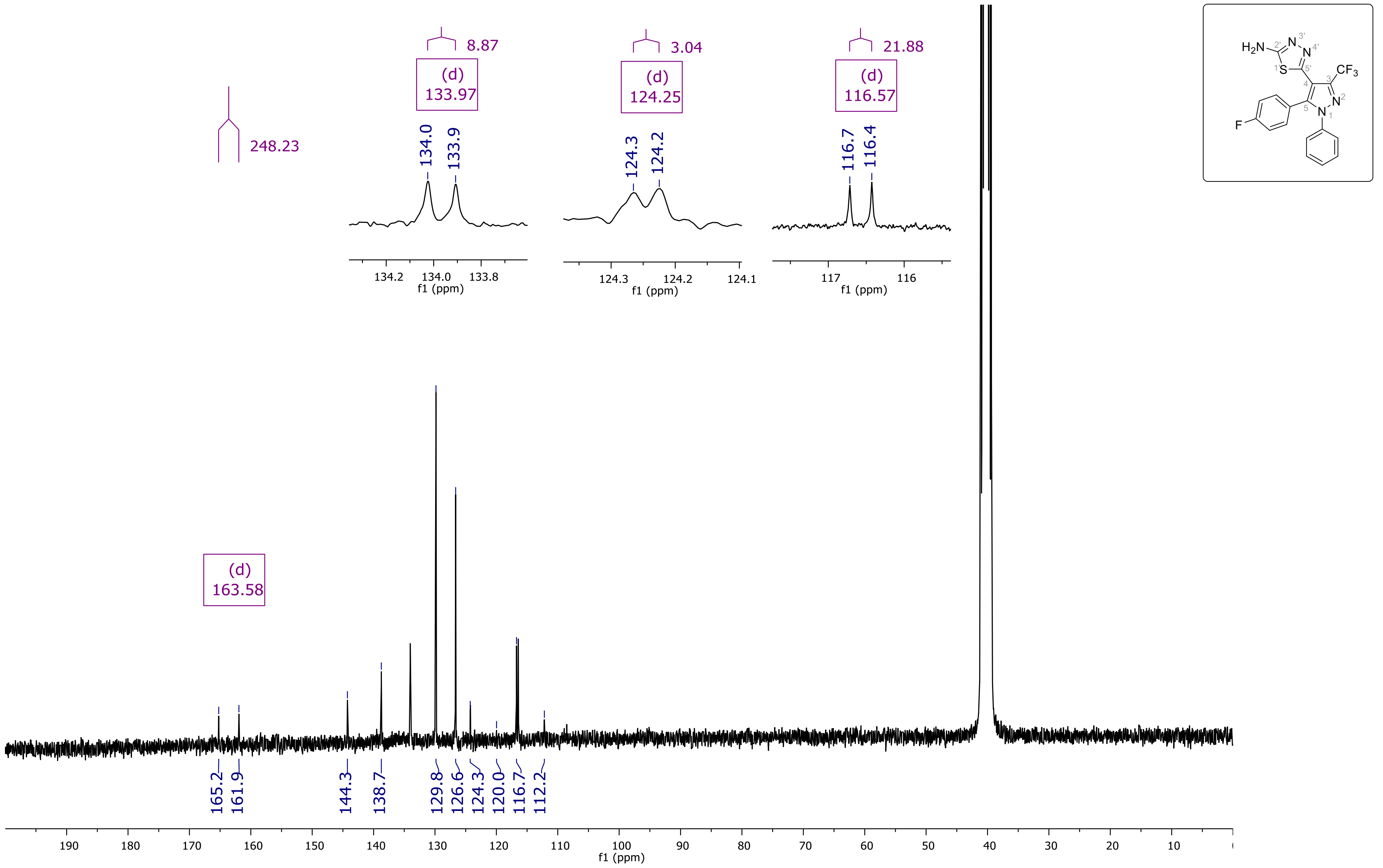

Figure S28 – ^{13}C NMR spectrum of compound **4b** in DMSO-d_6 at 75.46 MHz.

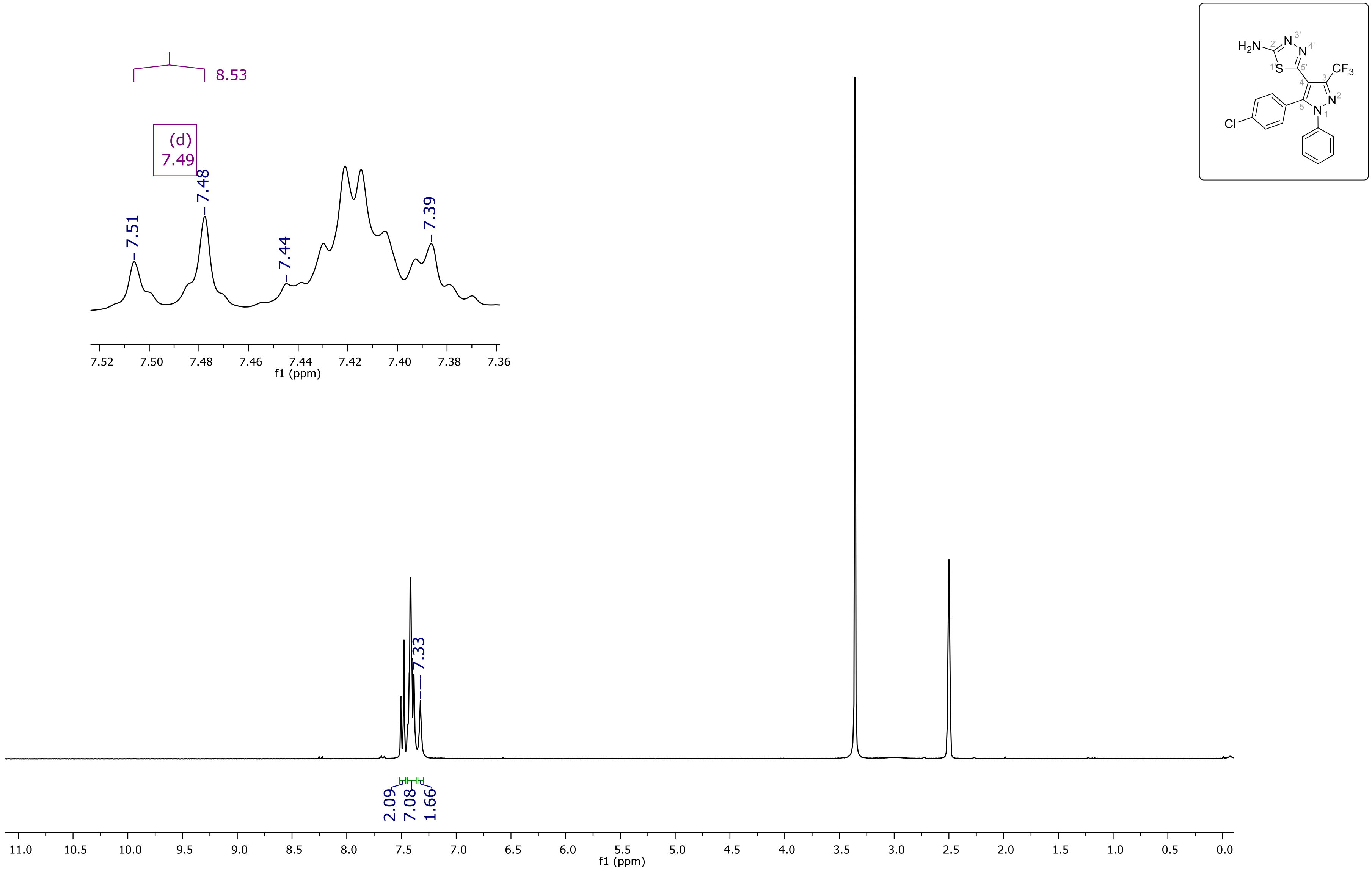

Figure S29 – ^{1}H NMR spectrum of compound **4c** in DMSO-d_6 at 300.06 MHz.

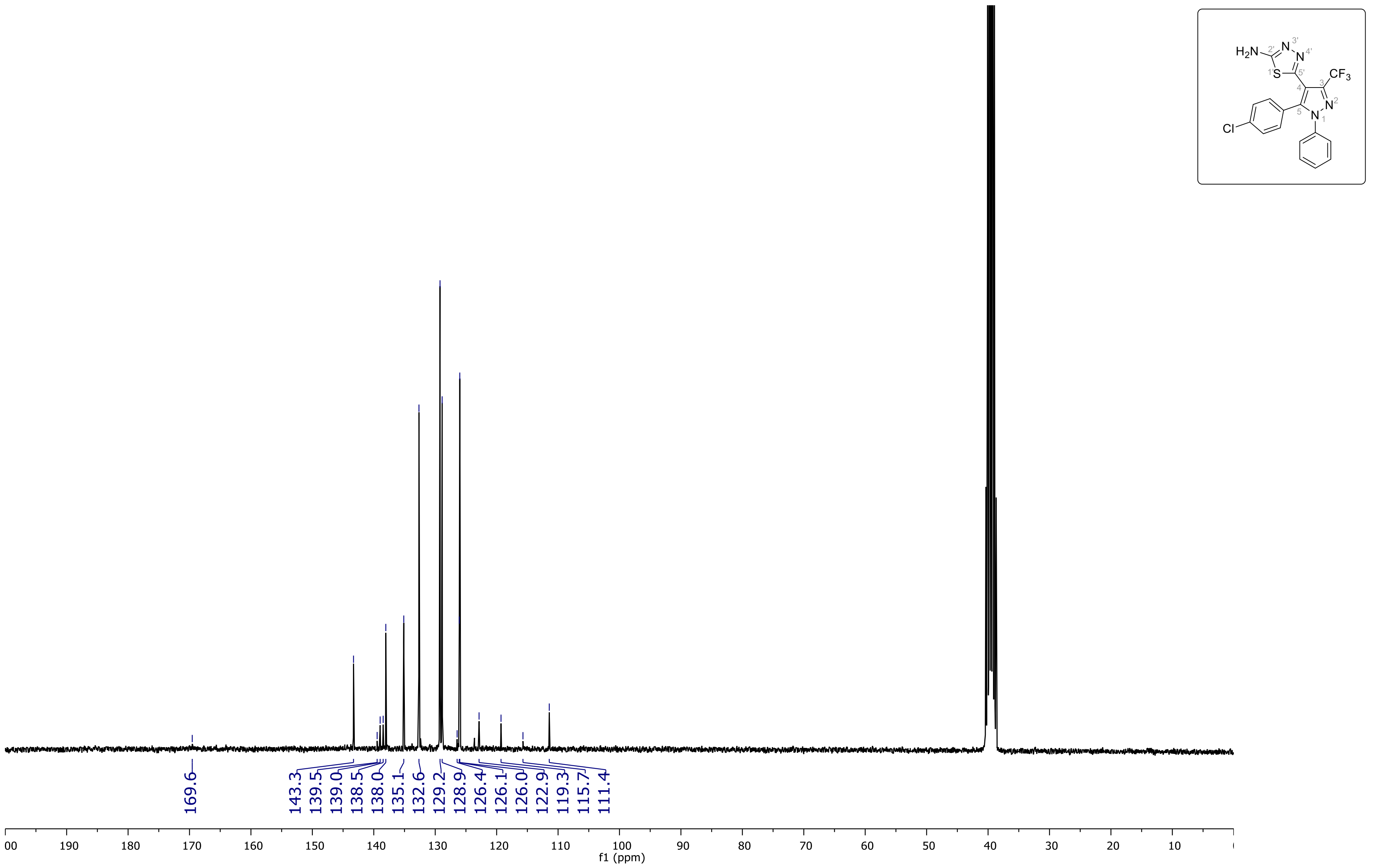

Figure S30 – ^{13}C NMR spectrum of compound **4c** in DMSO-d_6 at 75.46 MHz.

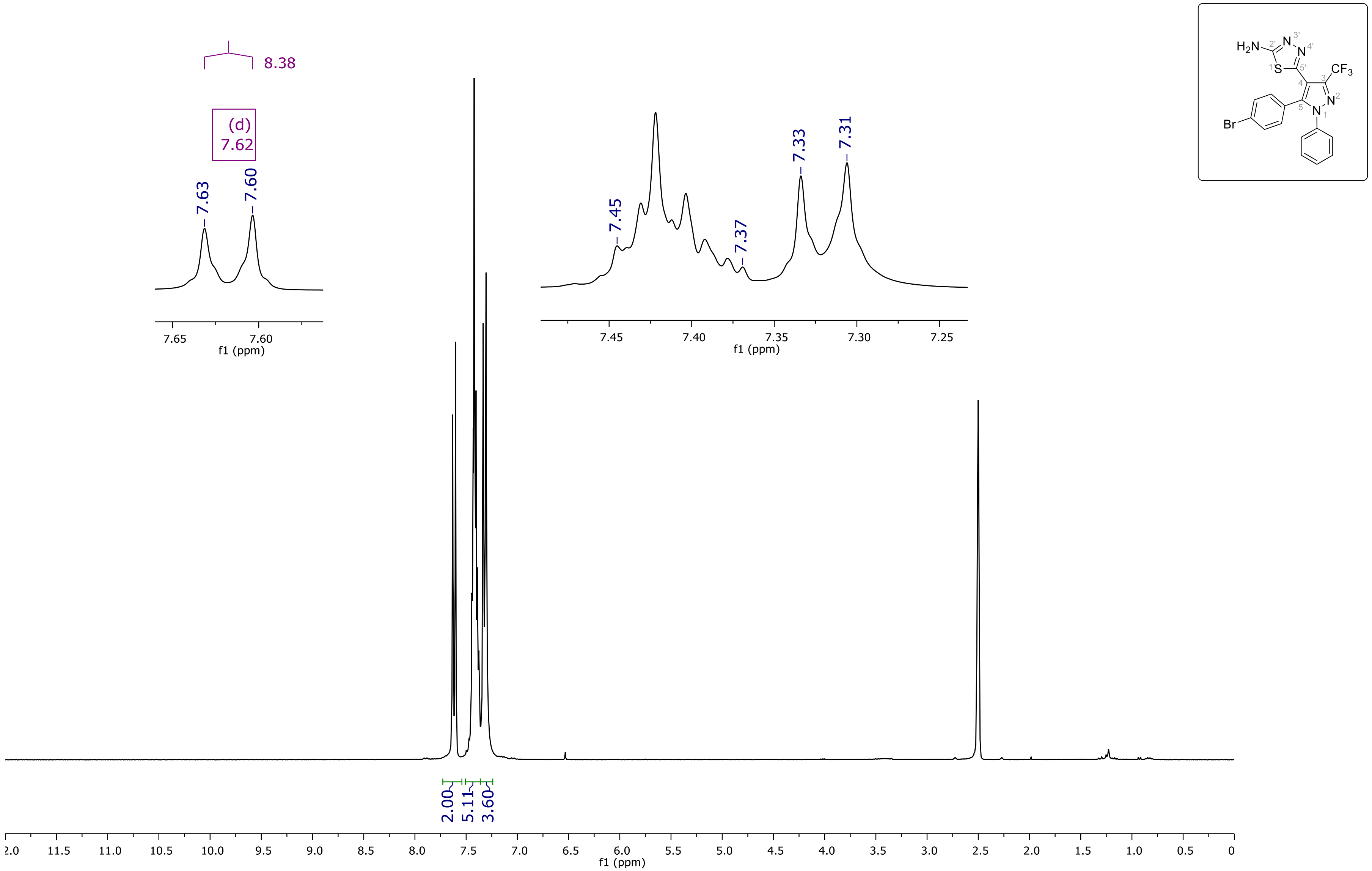

Figure S31 – ^{1}H NMR spectrum of compound **4d** in DMSO-d_6 at 300.06 MHz.

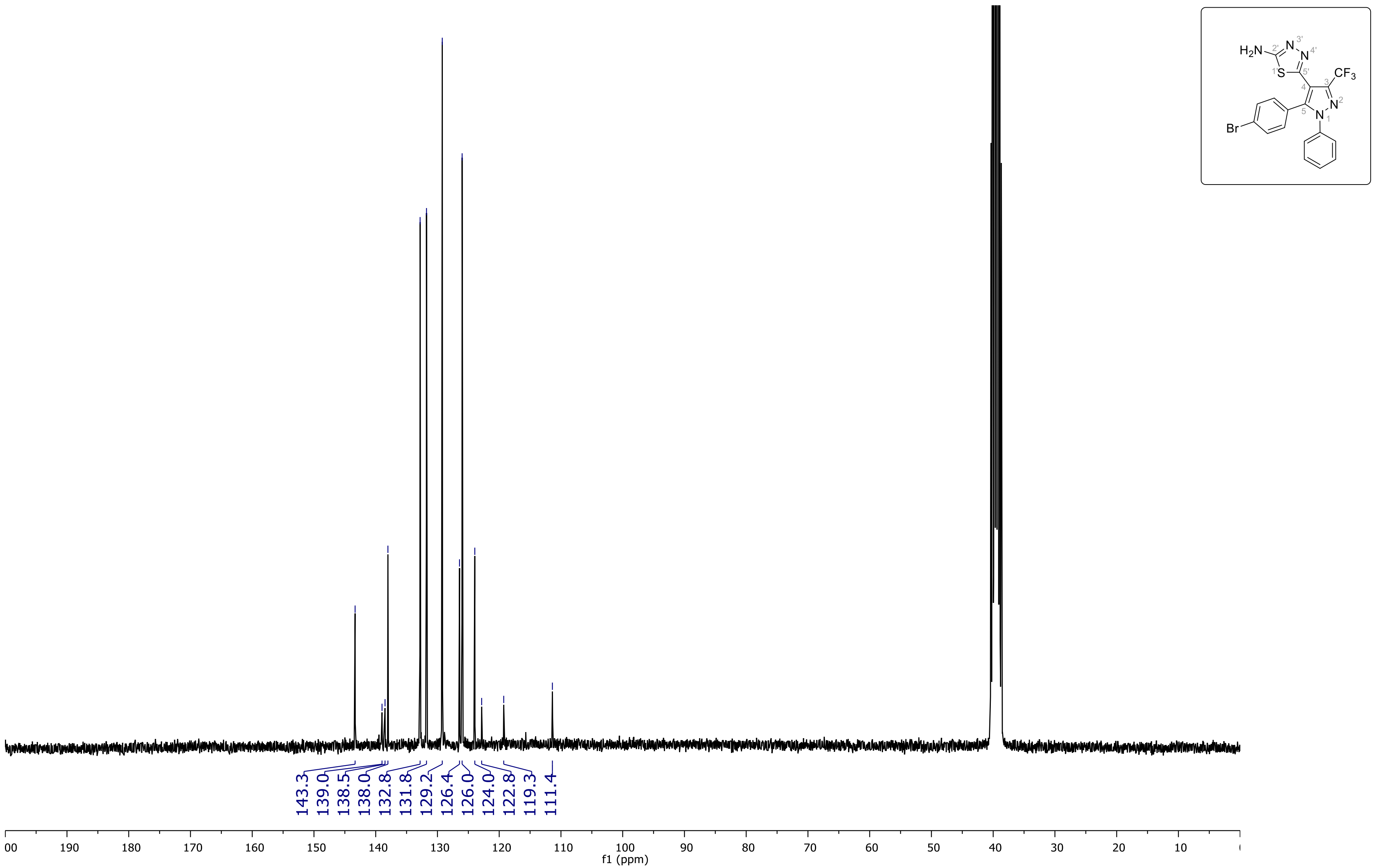

Figure S32 – ^{13}C NMR spectrum of compound **4d** in DMSO-d_6 at 75.46 MHz.

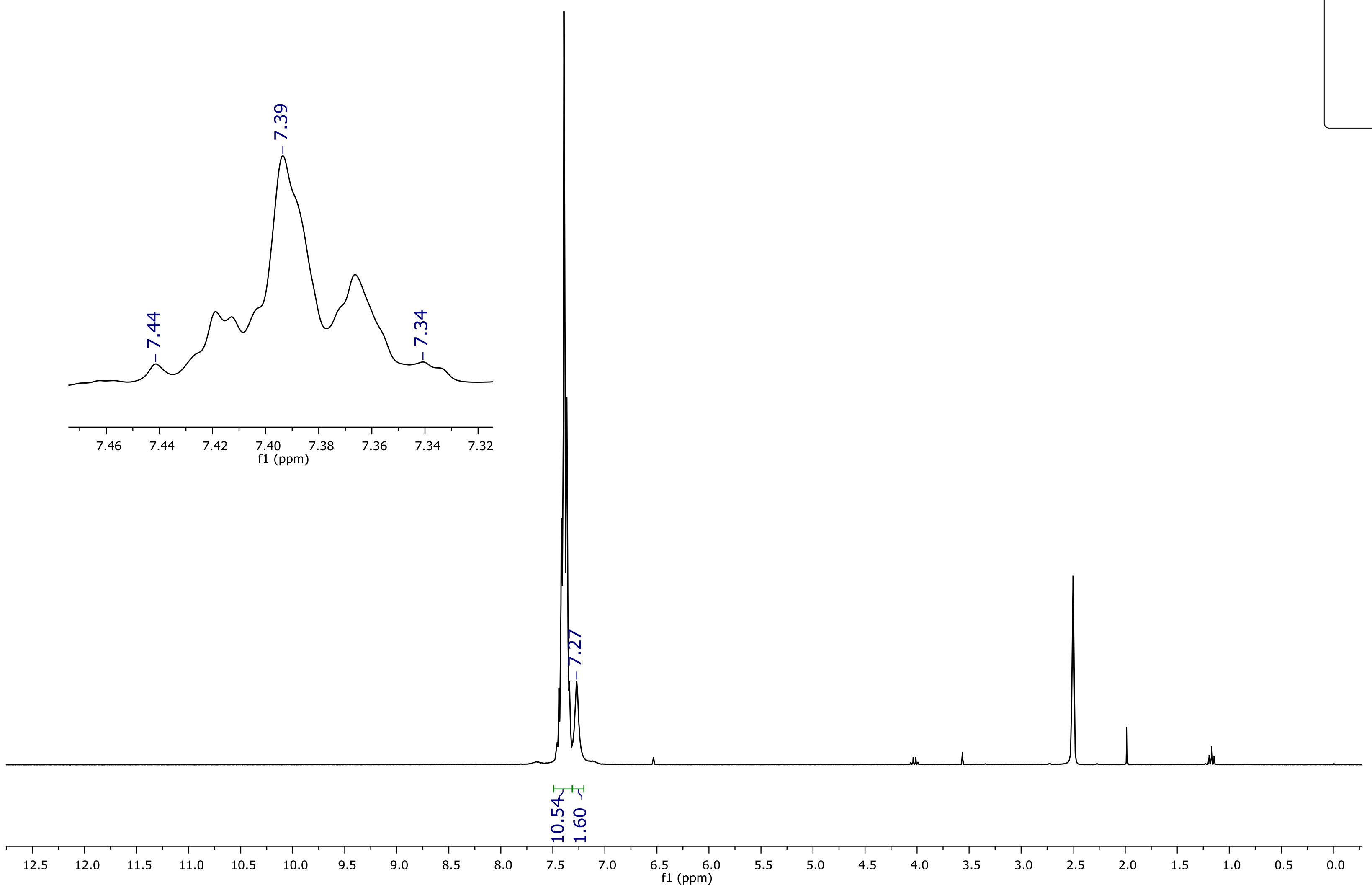

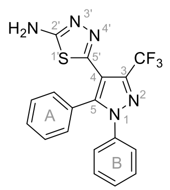

Figure S33 – ^{1}H NMR spectrum of compound **4e** in DMSO-d_6 at 300.06 MHz.

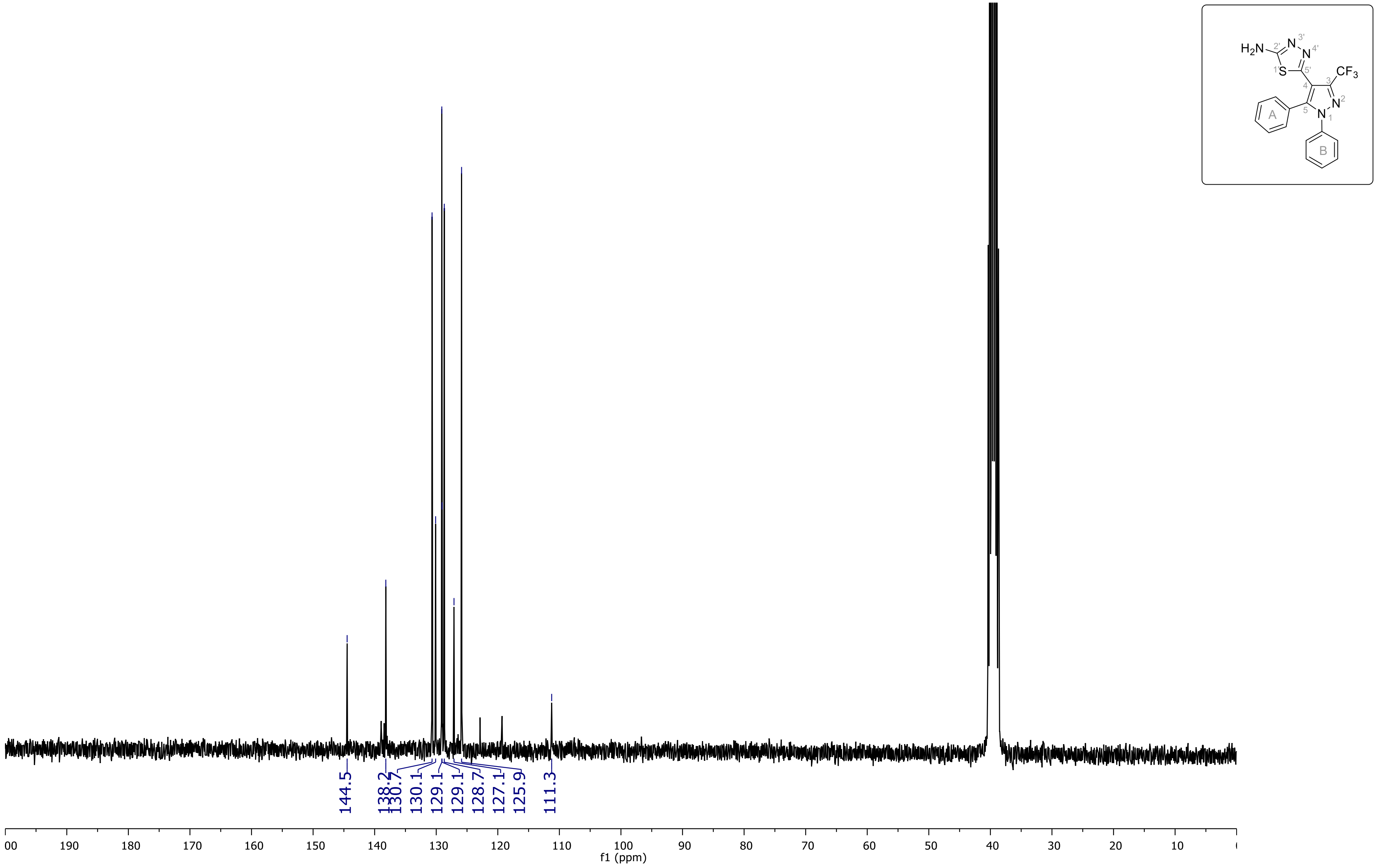

Figure S34 – ^{13}C NMR spectrum of compound **4e** in DMSO-d_6 at 75.46 MHz.

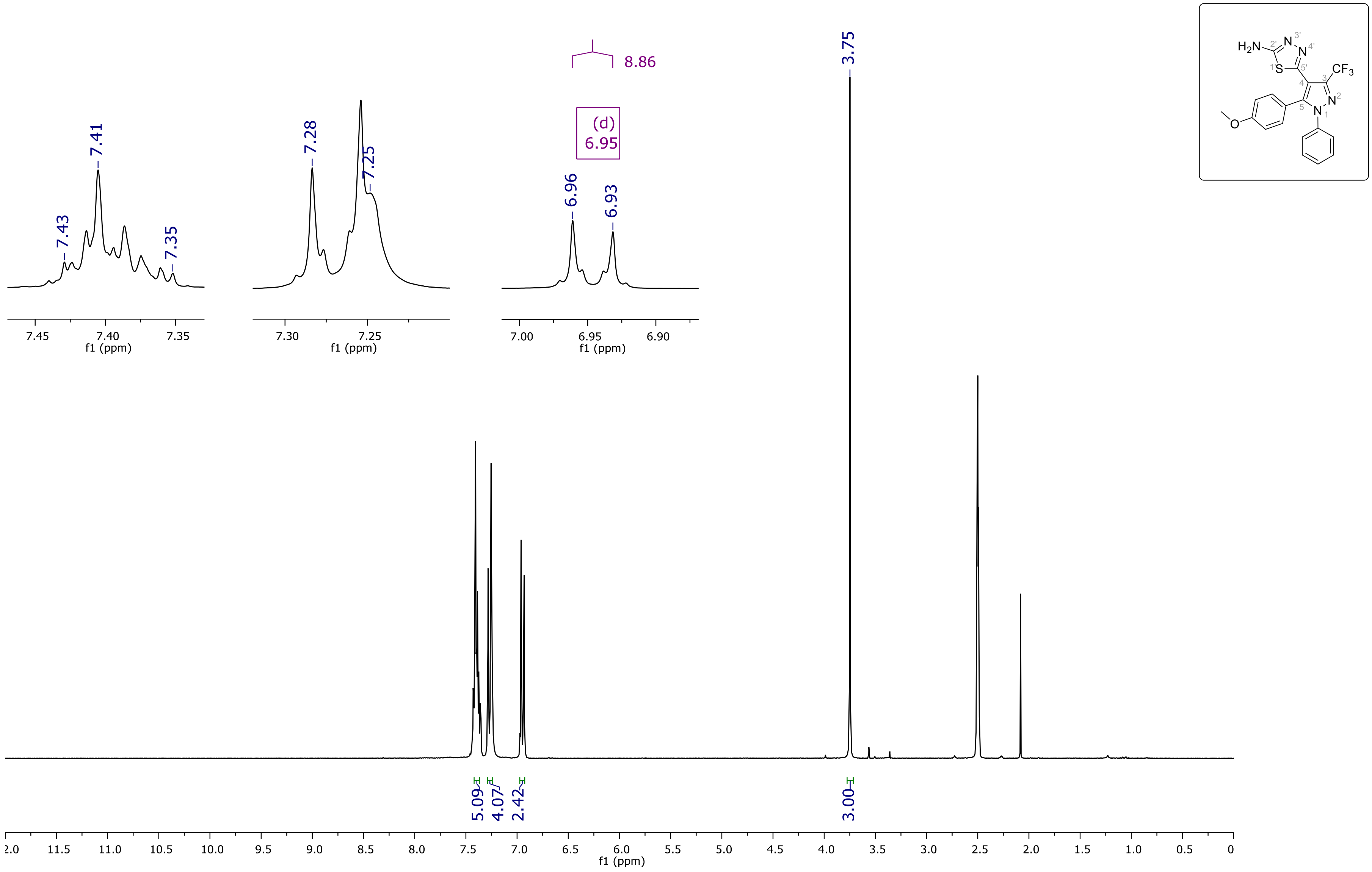

Figure S35 – ^{1}H NMR spectrum of compound **4f** in DMSO-d_6 at 300.06 MHz.

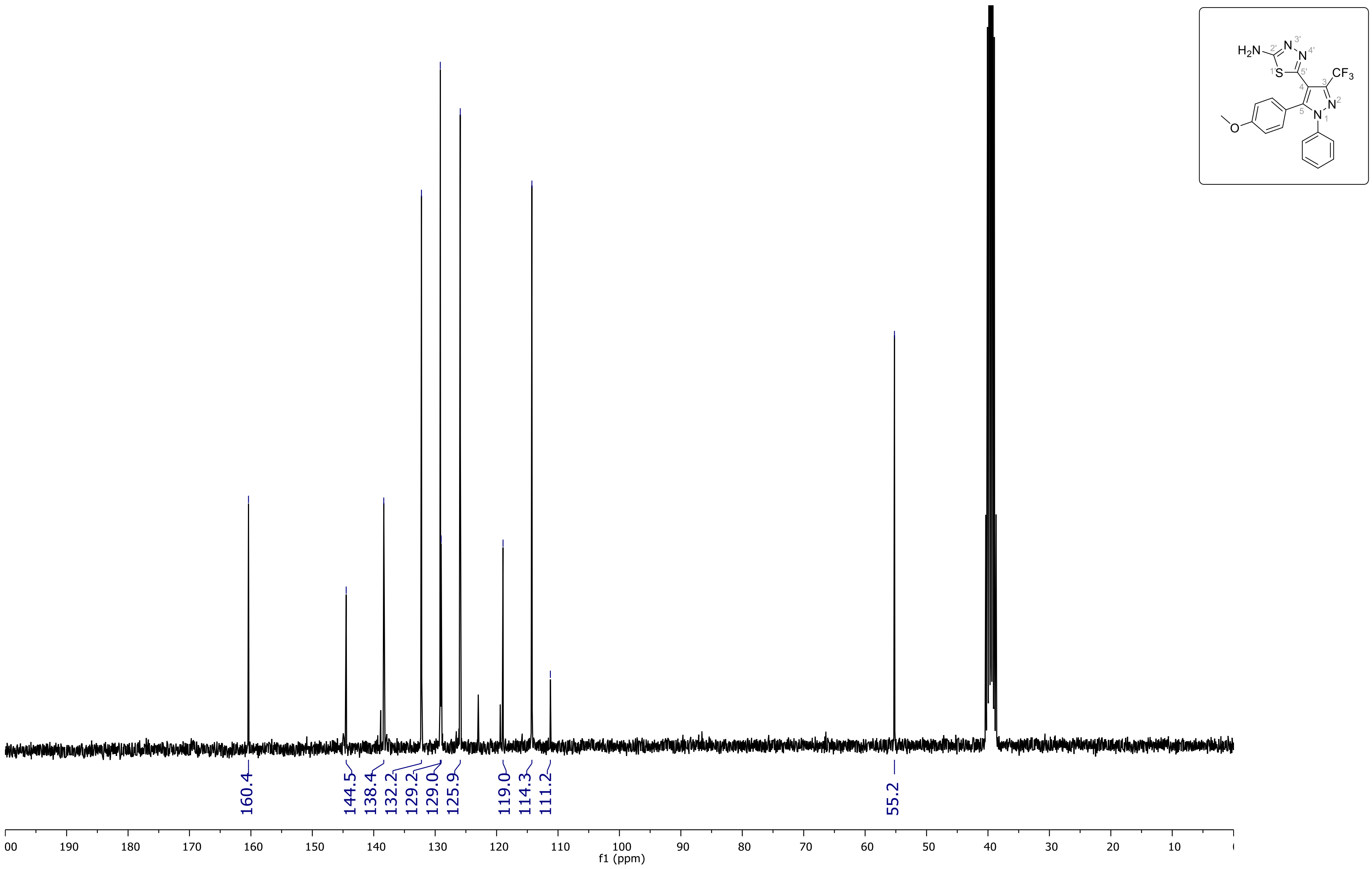

Figure S36 – ^{13}C NMR spectrum of compound **4f** in DMSO-d_6 at 75.46 MHz.

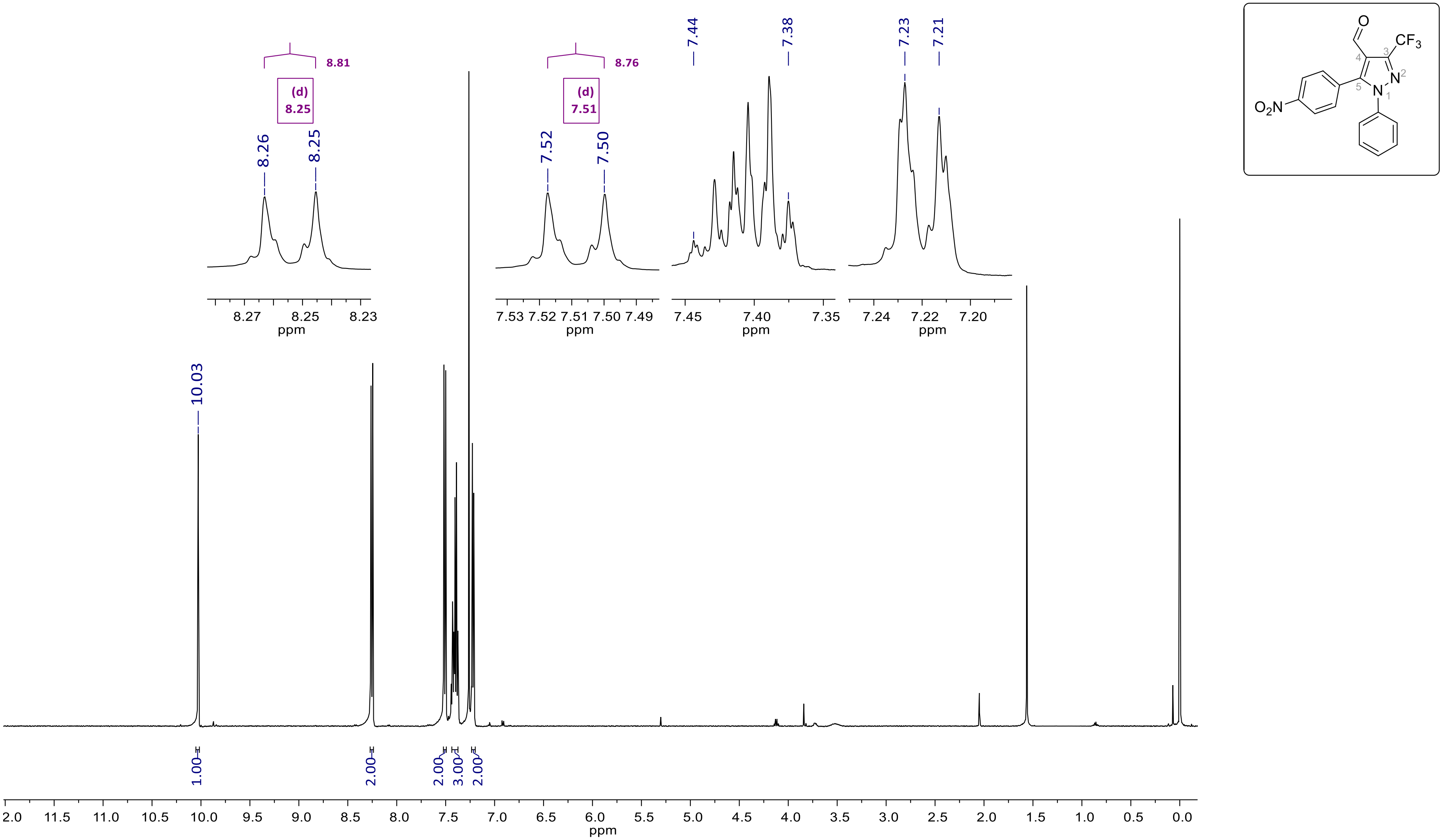

Figure S37 – ^{1}H NMR spectrum of compound **5a** in $CDCl_3$ at 300.06 MHz.

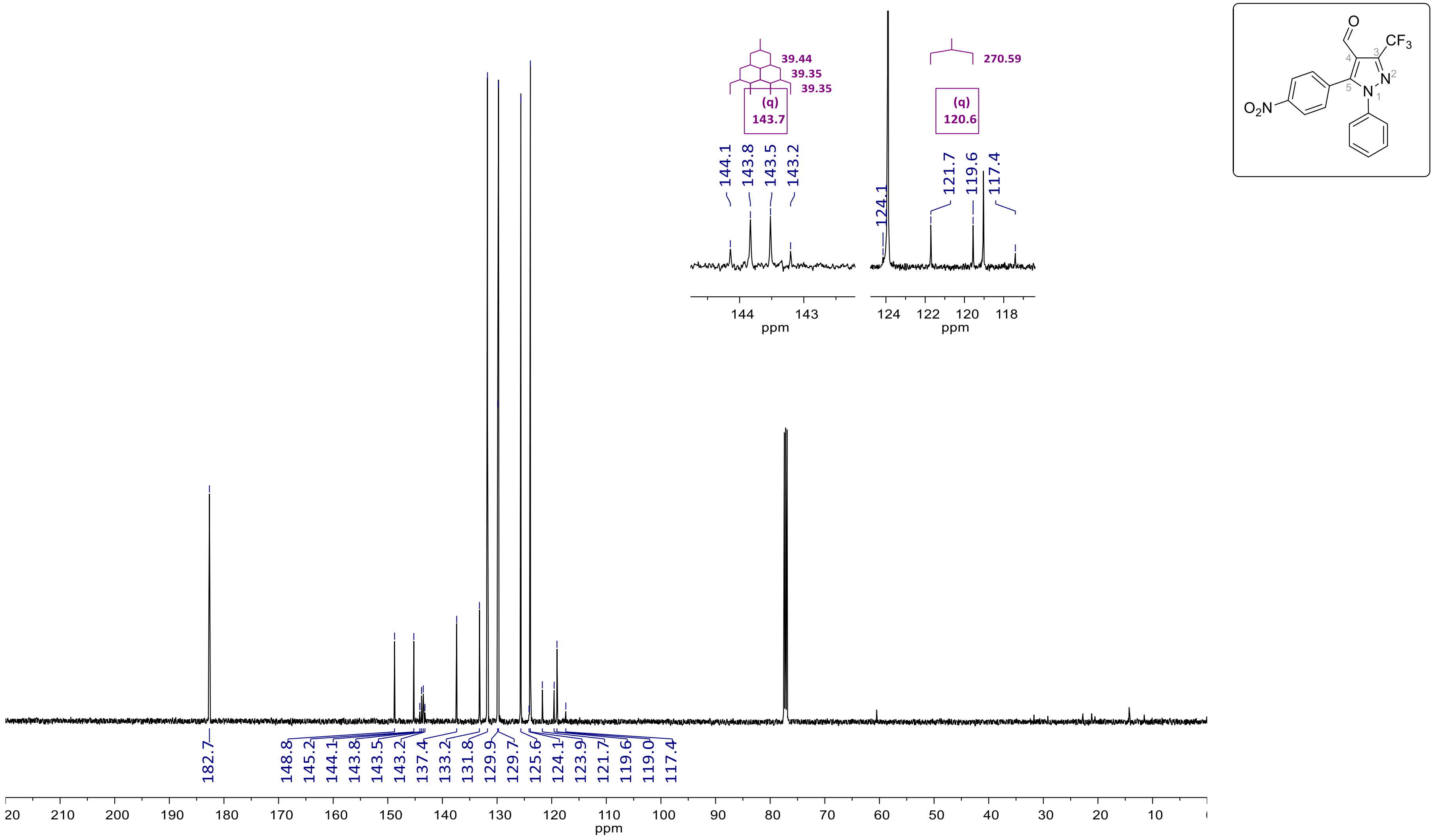

Figure S38 – ^{13}C NMR spectrum of compound **5a** in $CDCl_3$ at 75.46 MHz.

References

1. Armarego, W. L. F., Perrin, D. D. (1996). Purification of laboratory chemicals. Woburn: Butterworth Heinemann.

2. Pianoski, K. E., Poletto, J., Da Silva, M. J. V., Camargo, J. N. A., Jacomini, A. P., Gonçalves, D. S., Back, D. F., Moura, S., Rosa, F. A. R. (2020). 1,2-Addition to trifluoromethylated β-enamino diketones: Regioselective synthesis of trifluoromethyl-containing azomethine pyrazoles and isoxazoles. *Org. Biomol. Chem.*, 18, 2524–2537. doi: 10.1039/d0ob00319k

3. Niu, P., Kang, J., Tian, X., Song, L., Liu, H., Wu, J., Yu, W., Chang, J. (2015). Synthesis of 2-amino-1,3,4-oxadiazoles and 2-Amino-1,3,4-thiadiazoles via sequential condensation and I2-mediated oxidative C-O/C-S bond formation, *J. Org. Chem.*, 80 (2), 1018–1024.
